# Engineering the paclitaxel biosynthetic pathway in *Physcomitrium patens* for de novo and stable biosynthesis

**DOI:** 10.1038/s41467-026-76625-x

**Published:** 2026-10-21

**Authors:** Mengkai Zhao, Wenchang Lu, Renhai Peng, Liyan Wang, Ali Raza, Bin Jia, Zhangli Hu

**Affiliations:** 1 Guangdong Technology Research Center for Marine Algal Biotechnology, Shenzhen Engineering Laboratory for Marine Algal Biotechnology, Longhua Innovation Institute for Biotechnology, College of Life Sciences and Oceanography, Shenzhen University, Shenzhen, China; 2 Shenzhen Collaborative Innovation Public Service Platform for Marine Algae Industry, Guangdong Provincial Key Laboratory for Plant Epigenetics, College of Life Sciences and Oceanography, Shenzhen University, Shenzhen, China; 3 Guangdong Provincial Key Laboratory of Functional Substances in Medicinal Resources and Healthcare Products, School of Life Sciences and Food Engineering, Hanshan Normal University, Chaozhou, China; 4 Hunan Innovation Center for Synthetic Biomanufacturing Industry, Hunan University of Arts and Science, Changde, China

**Keywords:** Metabolomics, Synthetic biology, Synthetic biology

## Abstract

Paclitaxel is a highly valuable diterpenoid anticancer drug originally extracted from the yew tree. Due to the low efficiency of plant extraction and the high cost of semi-synthesis, efforts have been made to produce paclitaxel through biosynthesis. However, there have been no reports to date on engineered strains capable of paclitaxel biosynthesis. Here, we engineer a paclitaxel-producing line using the moss (*Physcomitrium patens*) chassis cells by combining transient expression verification, cytoplasmic localization strategies, self-cleaving 2 A peptide-mediated multi-gene co-expression systems and a modular iterative transformation strategy for stable genetic inheritance. Even after continuous subculturing multiple times, the engineered paclitaxel-producing lines obtained through both the initial reconstructed pathway and the optimized pathway are able to stably produce paclitaxel. This study proposes that *Physcomitrium patens* can serve as a plant chassis for paclitaxel biosynthesis.

## Abstract (web summary)

Paclitaxel is a highly valuable diterpenoid anticancer drug originally extracted from the yew tree. Here the authors genetically engineer the moss, *Physcomitrium patens*, for de novo paclitaxel biosynthesis.

## Introduction

Paclitaxel (Taxol), recognized as the most outstanding natural broad-spectrum anticancer drug discovered to date, is widely used to treat various cancers. Originally extracted primarily from the bark of the rare *Taxus* tree, its yield is extremely low, and *Taxus* grows slowly^1^. Paclitaxel’s chemical structure is highly complex, with a central core and side chain containing 11 stereocenters, making chemical synthesis challenging, with low yields and high costs^2^. Both production methods result in expensive paclitaxel. With the elucidation of the *Taxus* genome^3–5^ and advances in synthetic biology, the biosynthesis of paclitaxel represents a highly promising solution.

The paclitaxel biosynthetic pathway involves at least 18 enzymatic steps^6^, divided into three stages: formation of the taxane skeleton baccatin III; synthesis of the phenylalanine-CoA side chain; and coupling of the baccatin III skeleton’s C13 acyl group with the side chain, followed by subsequent modifications^7^. The detailed process is as follows: first, the methylerythritol phosphate (MEP) pathway synthesizes the common diterpenoid precursor geranylgeranyl pyrophosphate (GGPP), which is then catalyzed by taxadiene synthase (TXS) to form the taxane skeleton taxa-4(5),11(12)-diene. Taxadiene undergoes hydroxylation or isomerization by multiple P450 enzymes (at C1, C2, C5, C7, C9, C10, and C13 positions) to yield baccatin III. Next, α-phenylalanine aminomutase (PAM) and β-phenylalanine-CoA ligase (PCL) isomerize α-phenylalanine to β-phenylalanine and conjugate it with acetyl-CoA to form β-phenylalanyl-CoA. Finally, baccatin III: 3-amino-3-phenylpropanoyl-transferase (BAPT) catalyzes the C13 acylation of baccatin III (using β-phenylalanyl-CoA as the acyl donor) to produce β-phenylalanyl baccatin III, which is then hydroxylated at the C2′ position and benzoylated at the C3′ position to ultimately form paclitaxel^7^.

Researchers have spent decades deciphering paclitaxel’s biosynthetic pathway. In recent years, multiple teams have made breakthrough progress in elucidating this pathway^6,8–12^. In 2023, the Fernie team achieved a critical breakthrough in the heterologous biosynthesis of paclitaxel. The team identified a novel C4β–C20 epoxidase, successfully overcoming the first major bottleneck in metabolic engineering. By integrating both previously known and newly discovered enzymes, including oxomutases/epoxidases, taxane 1β-hydroxylase, taxane 9α-hydroxylase, taxane 9α-dioxygenase, and phenylalanine-CoA ligase, they successfully achieved the biosynthesis of the key intermediate baccatin III and further converted it into paclitaxel in *Nicotiana benthamiana* (*N. benthamiana*) using a transient expression system^8^.

In 2024, the Yan team identified a bifunctional cytochrome P450 enzyme, taxane oxetanase 1 (TOT1), which catalyzes the key oxidative rearrangement reaction for oxetane ring formation in paclitaxel biosynthesis. The team also discovered the taxane 9a hydroxylase 1 (T9αH1) enzyme responsible for the oxidation at the C9 position of the taxane skeleton. Based on these key findings, they successfully achieved the biosynthesis of baccatin III in tobacco using the *N. benthamiana* transient expression system^9^.

The Zhou lab soon achieved the total synthesis of 1β-dehydroxybaccatin VI from taxa-4(20),11(12)-diene-5α-ol in engineered yeast. By resolving the sequence of key hydroxylation steps, identifying the taxane 9α-hydroxylase and the CYP725A55 enzyme responsible for oxetane ester formation, together with the newly discovered C7‑acetyltransferase^10^.

In 2025, the Sattely team identified the nuclear transport factor 2-like protein-facilitator of taxane oxidation (FoTO1), leading to a effectively enhancement in the production yield of taxadien-5α-ol. In combination with a newly discovered β-phenylalanine-CoA ligase, this study revealed a total of eight key genes, which collectively enabled the biosynthesis of 3′-N-debenzoyl-2′-deoxypaclitaxel^11^.

Recent genomic analysis of *Pseudotaxus chienii* revealed that its taxane biosynthetic pathway terminates prior to the key enzyme TBT (taxane 2α-O-benzoyl transferase), preventing the synthesis of 10-deacetylbaccatin III. The study also found that the metal ion catalytic site structure of taxadiene synthase (TS) is highly conserved between the two genera of *Pseudotaxus* and *Taxus*, providing a potential target for enhancing TS activity through enzyme engineering^12^.

Different research teams have successfully biosynthesized paclitaxel or its precursors in tobacco or yeast using distinct gene combinations, which suggests that the paclitaxel biosynthetic pathway is not strictly linear or unique but rather constitutes a dynamic synthetic network^7,10,13^. Due to the extreme complexity of the biosynthetic pathway of paclitaxel, its complete mechanism—including the involved enzyme genes, cofactors, regulatory factors, as well as the catalytic order and network structure—has not yet been fully elucidated and awaits gradual revelation in the future. Nevertheless, this has not hindered researchers from advancing the biosynthesis of paclitaxel and its precursors.

Currently, the biosynthetic pathways of paclitaxel intermediates have been reconstituted in various chassis cells, including *Escherichia coli* (*E. coli*), *Saccharomyces cerevisiae* (*S. cerevisiae*), *N. benthamiana*, *Nicotiana tobacum* (*N. tobacum*) and *Arabidopsis thaliana* (*A. thaliana*). In *E. coli*, Ajikumar’s team achieved high-yield production (570 ± 45 mg/L) of oxygenated taxanes through N-terminal modification of P450 enzymes^14^, while Walker's team overexpressed 10β-acetyltransferase to synthesize baccatin III from exogenous 10-DAB^15^. However, prokaryotic systems face limitations, such as insufficient supply flux of natural precursors, protein insolubility, inadequate NADPH supply, and lack of subcellular compartmentalization^16^.

Eukaryotic systems offer some key advantages. For instance, *A. thaliana* produced taxadiene (600 ng/g DW) via glucocorticoid-induced TXS expression^17^. Wang Yong’s team achieved 56.6 µg/g (taxadiene) and 1.3 µg/g (T5α-ol) fresh weight in *N. benthamiana* using chloroplast compartmentalization^18^. Zhang’s group improved taxadiene yield to 87.8 μg/g (DW) via chloroplast-targeted expression in transgenic *N. tobacum*^19^. Later pathway advances include Kampranis’ heterologous paclitaxel production (0.97 ± 0.05 µg/L) in yeast with exogenous precursors^6^, and Sattely’s team elevated baccatin III production in *N. benthamiana* to natural *Taxus* needles levels via transient expression^11^. However, current production systems still predominantly rely on transient transformation techniques or semi-synthetic engineered lines. Consequently, the stable integration of the paclitaxel biosynthetic pathway into chassis genomes remains a critical and core objective for achieving sustainable and scalable production.

In this study, we select *Physcomitrium patens* (*P. patens*) as the chassis cell, which is often referred to as the green yeast^20^, exhibits typical advantages of eukaryotic expression systems, such as sufficient supply of key precursor substances like GGPP and ample NADPH^21^, the correct folding and subcellular compartmentalization of heterologous eukaryotic proteins. Furthermore, this system is operationally convenient: it allows easy preparation of protoplasts and is suitable for rapid gene function validation via transient expression^22^. Its nuclear genome exhibits high homologous recombination efficiency, facilitating precise integration of large foreign DNA fragments into specific loci^23^. In addition, the plant demonstrates strong regenerative capacity and can efficiently regenerate into intact plants from protoplasts^23^, making it an ideal plant platform for synthetic biology and metabolic engineering research.

Given that the Fernie team reported earlier the minimal gene set required for paclitaxel biosynthesis^8^, and considering that the yield of baccatin III in their system was higher than that achieved in the Yan team’s approach^9^, indicating superior application potential. Through the integrated application of previously characterized enzymes and those newly identified by the Fernie team^3,8,24^, we consequently select this pathway as the initial reconstructed paclitaxel biosynthetic pathway and successfully perform its validation using a transient expression system in *P. patens*. As FoTO1, founded by McClune et al., significantly improved the yield of taxadien-5α-ol^11^, and T2′OGD/T3′NBT discovered by Liang et al. can increase the yield of paclitaxel^6^, we incorporate FoTO1, T2′OGD and T3′NBT into the subsequent optimization of the initial reconstructed paclitaxel biosynthetic pathway. We will continue to further optimize the constructed engineered paclitaxel-producing line in follow-up studies. The goal of this study is not to elucidate the biosynthetic pathway of paclitaxel with full mechanistic resolution, but rather to biosynthesize paclitaxel using *P. patens* based on a known set of genes.

**Fig. 1:**
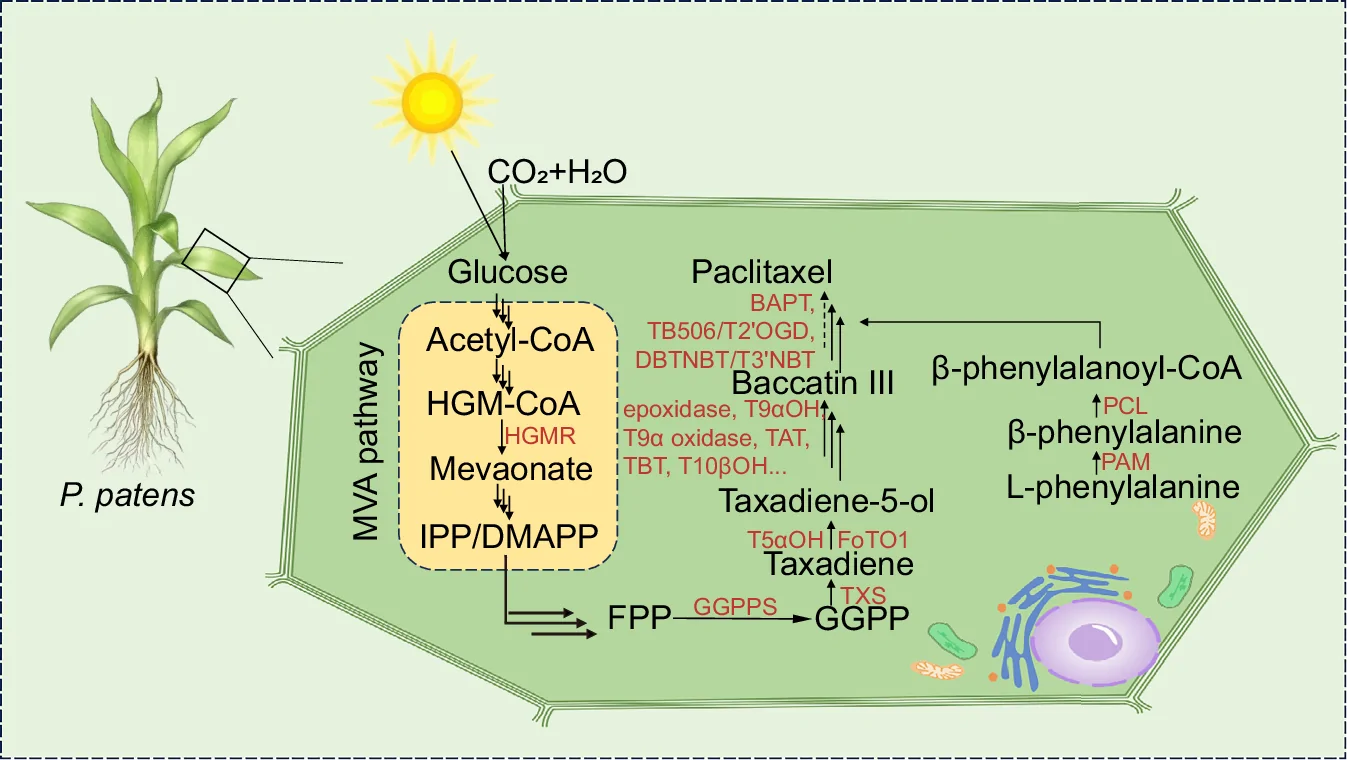
Engineered biosynthetic pathway for paclitaxel production in *P. patens.* The heterologous synthesis pathway utilizes universal terpenoid precursors IPP and DMAPP supplied by the endogenous MVA pathway. These precursors are condensed to form GGPP, which is subsequently converted to paclitaxel through catalysis by a heterologously introduced suite of paclitaxel biosynthetic enzymes. Acetyl-CoA acetyl-coenzyme A, HGM-CoA 3-hydroxy-3-methylglutaryl coenzyme A, HMGR 3-hydroxy-3-methylglutaryl-CoA reductase, IPP isopentenyl pyrophosphate, DMAPP dimethylallyl pyrophosphate, FPP, farnesyl pyrophosphate, GGPPS geranylgeranyl diphosphate synthase, GGPP geranylgeranyl diphosphate, TXS taxadiene synthase, T5αOH taxane 5α-hydroxylase, TAT taxadiene-5α-ol-O-acetyl transferase, T10βOH taxane 10β-hydroxylase, T13αOH taxane 13α-hydroxylase, T2αOH taxane 2α-hydroxylase, T9αOH taxane 9α-hydroxylase, T9α oxidase taxane 9α-oxidase, T7βOH taxane 7β-hydroxylase, T1βOH taxane 1β-hydroxylase, TBT taxane-2α-O-benzoyltransferase, DBAT 10-deacetyl baccatin III-10-O-acetyltransferase, BAPT baccatin III-3-amino, 13-phenylpropanoyltransferase, T2′αOH taxane 2′α-hydroxylase, DBTNBT 3′-N-debenzoyl-2′-deoxytaxol-N-benzoyltransferase, PAM phenylalanine aminomutase, PCL β-phenylal-anine coenzyme A ligase. β-phenylalanoyl-CoA β-phenylalanoyl-coenzyme A, FoTO1 facilitator of taxane oxidation, T2′OGD taxoid-2′-oxoglutarate-dependent dioxygenase, T3′NBT taxoid-3′-N-benzoyltransferase.

In this study, we employ transient-stable combined engineering strategy (TSCES) and modular iterative transformation (MIT) strategy to respectively construct the initial reconstructed pathway and the optimized pathway for paclitaxel biosynthesis based on *P. patens* (Fig. 1), and obtain a genetically stable engineered strain producing paclitaxel.

## Results

### Biosynthesis of paclitaxel precursors

To improve the efficiency of constructing paclitaxel-engineered lines, we adopted the TSCES and first employed a transient expression system in *P. patens* to validate whether the combination of genes involved in the paclitaxel biosynthetic pathway could effectively synthesize paclitaxel and its precursors. All gene sequences used were codon-optimized according to the codon preference of *P. patens*.

The biosynthesis of paclitaxel initiates with GGPP as the precursor substrate. GGPP is synthesized through the condensation of three isopentenyl pyrophosphate (IPP) molecules and one dimethylallyl pyrophosphate (DMAPP) molecule, catalyzed by geranylgeranyl pyrophosphate synthase (GGPPS)^25^. In plants, IPP and DMAPP are produced via two pathways: the plastidial MEP pathway, which directly supplies both precursors for early taxane synthesis, and the cytosolic mevalonate (MVA) pathway, where IPP is converted to DMAPP by IDI^26^. In *P. patens*, both pathways coexist, enabling chloroplasts and the cytosol to provide substrates for GGPPS^21^. Subsequently, TXS cyclizes GGPP to form taxadiene—the first cyclic intermediate and core scaffold of paclitaxel.

**Fig. 2:**
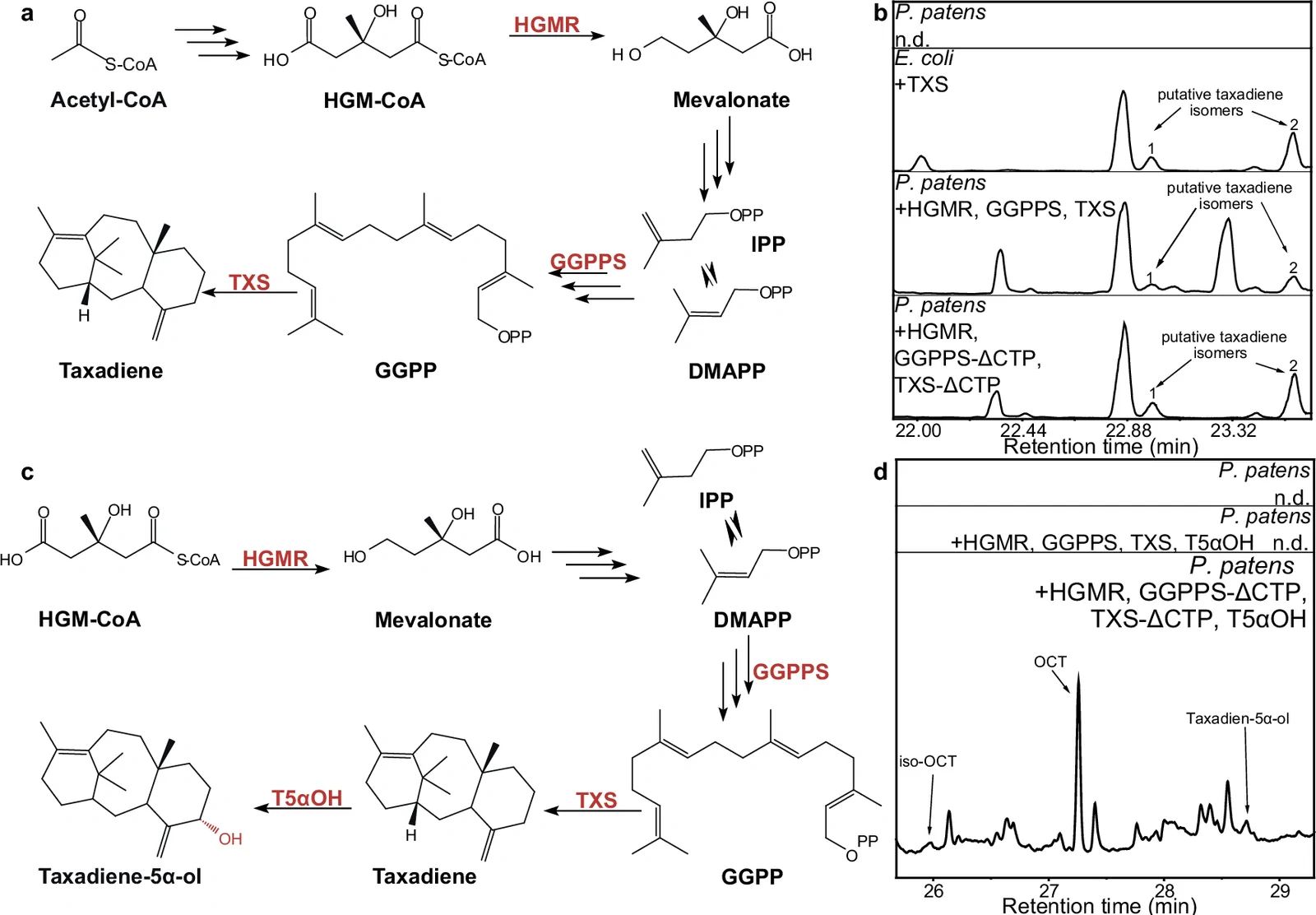
Transient expression in *P. patens* enables heterologous biosynthesis of taxadiene and taxadiene-5α-ol. **a** Proposed pathway from GGPP to taxadiene. **b** GC-MS detection of taxadiene in transiently transformed protoplasts. **c** Proposed pathway from taxadiene to taxadiene-5α-ol. **d** GC-MS detection of taxadiene-5α-ol in transiently transformed protoplasts. n.d., not detected; TXS-ΔCTP, TXS without chloroplast transit peptide; GGPPS-ΔCTP, GGPPS without chloroplast transit peptide. *E. coli* +TXS, taxadiene-producing *E. coli* positive control. Source data are provided as a Source Data file.

To enhance the conversion efficiency of IPP, we co-expressed *GGPPS*, *TXS*, and *HMGR* genes under the control of the p35S strong promoter in *P. patens* protoplasts via transient expression. Gas chromatography-mass spectrometry (GC-MS) analysis revealed that, compared with the product produced in *E. coli*, taxadiene-4(20), 11(12)-diene and taxadiene-4(5), 11(12)-diene were detected (Fig. 2a, b, and Supplementary Fig. 1). The mass spectra of these isomers matched those of previously reported^27,28^ taxadiene-4(5), 11(12)-diene and taxa-4(20),11(12)-diene with *m/z* 272.2 (Supplementary Fig. 1).

Further transient co-expression of *GGPPS*, *TXS*, *HMGR*, and *T5αOH* in *P. patens* protoplasts did not yield detectable 5α-hydroxytaxadiene (Fig. 2c, d). Both GGPPS and TXS contain chloroplast transit peptides and are localized in the chloroplast, whereas HMGR and T5αOH are localized in the endoplasmic reticulum. We hypothesized that insufficient taxadiene transport from the chloroplast to the cytoplasm limited downstream reactions catalyzed by T5αOH. Therefore, we propose to remove the signal peptides of GGPPS and TXS, and prioritize the validation of their removal efficiency. Full-length and signal-peptide-deleted TXS and GGPPS were each fused to GFP (green fluorescent protein) and transiently expressed in *P. patens* protoplasts, and their subcellular localization was visualized by fluorescence microscopy (Supplementary Fig. 2). The results showed that the green fluorescent chimeras derived from TXS/GFP and GGPPS/GFP completely overlapped with the spontaneous red fluorescence of chloroplasts (Supplementary Fig. 2). In contrast, the green fluorescent derived from signal-peptide-deleted TXS and GGPPS were dispersed in the cytoplasm (Supplementary Fig. 2). This confirmed that we had successfully removed the signal peptides. Therefore, we removed the chloroplast transit peptides of *GGPPS* and *TXS* and co-expressed them with *HMGR* in *P. patens* protoplasts. This approach also resulted in detectable taxadiene (Fig. 2a, b). Subsequently, transient co-expression of these modified genes with *T5αOH* and *HMGR* in *P. patens* protoplasts enabled successful detection of taxadiene-5α-ol, OCT, iso-OCT with *m/z* 288.2 by GC-MS analysis (Fig. 2c, d, and Supplementary Fig. 3).

### Biosynthesis of baccatin III and Taxol

**Fig. 3:**
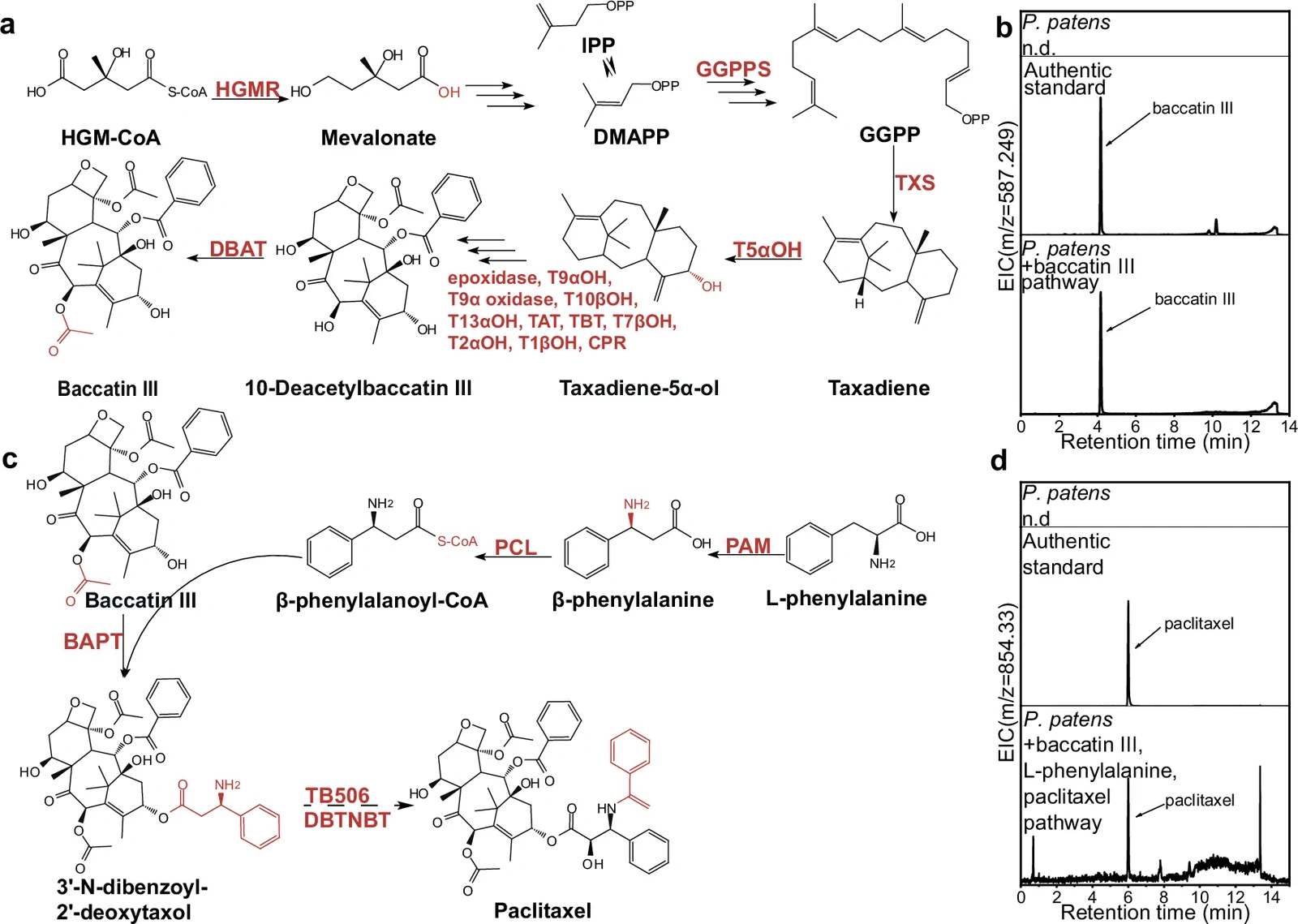
Transient expression in *P. patens* enables heterologous biosynthesis of baccatin III and paclitaxel. **a** Proposed pathway for the biosynthesis of baccatin III. **b** UPLC-qTOF-MS detection of baccatin III in transiently transformed protoplasts. **c** Proposed pathway for the biosynthesis of paclitaxel. **d** UPLC-qTOF-MS detection of paclitaxel in transiently transformed protoplasts. n.d., not detected. Source data are provided as a Source Data file.

We transiently co-expressed signal peptide-truncated *GGPPS* and *TXS* along with the remaining 14 genes required for baccatin III biosynthesis in *P. patens* protoplasts. Based on LC/MS analysis, baccatin III (high-resolution MS [HRMS] *m/z* 587.2482 [M+H]^+^ and 609.2288 [M+Na]^+^) was successfully detected (Fig. 3a, b, and Supplementary Fig. 4a). *BAPT*, *PCL* (Pc21g30650), *PAM*, *TB506*, and *DBTNBT* were transiently co-expressed in *P. patens* protoplasts, and the culture medium was supplemented with L-phenylalanine and baccatin III, leading to the successful detection of paclitaxel ([HRMS] *m/z* 854.3393 [M+H]^+^ and 876.3221 [M+Na]^+^) (Fig. 3c, d, and Supplementary Fig. 4b).

### Paclitaxel engineered strain design

After validating that the known gene set could produce paclitaxel in *P. patens* using a transient expression system (Fig. 3, Supplementary Fig. 4, and Supplementary Data 1), we continued to construct stably inherited taxol-engineered lines. The key considerations included the selection of promoters, the choice of target neutral genomic sites for integration, and the overall construction strategy.

**Fig. 4:**
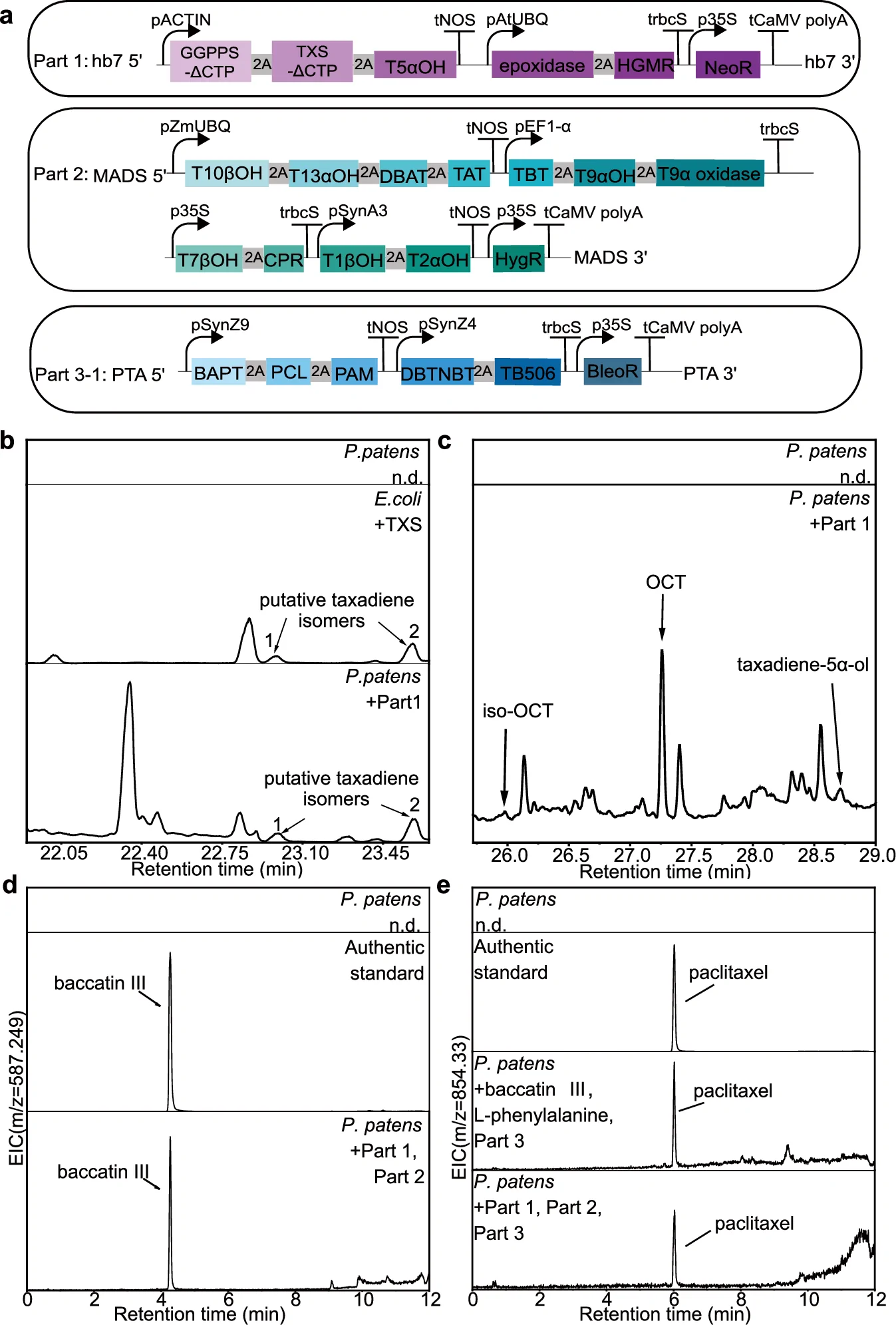
Stable expression of the complete paclitaxel biosynthetic pathway in *P. patens* using a modular vector system. **a** Schematic of the three-part assembly of heterologous gene modules: Part 1 (genes from *HMGR* to *T5αOH*) for the production of taxadiene-5α-ol; Part 1 + Part 2 (additional P450s and modifying enzymes) for the production of baccatin III; Part 1 + Part 2 + Part 3 (genes from *PAM* to *DBTNBT*) for the production of paclitaxel. 2A: 2A self-cleaving peptides. Selection markers: *NeoR* (G418 resistance), *HygR* (hygromycin resistance) and *BleoR* (zeocin resistance). GC-MS or UPLC-qTOF-MS chromatograms showing the production of taxa-4(20),11(12)-diene (1), taxa-4(5),11(12)-diene (2) (**b**), taxadiene-5α-ol, OCT, iso-OCT (**c**), baccatin III (**d**), and paclitaxel (**e**) in stable transgenic lines harboring the corresponding gene modules. n.d., not detected; *E. coli* +TXS, taxadiene-producing *E. coli* positive control. Source data are provided as a Source Data file.

To avoid transcriptional gene silencing caused by promoter homology^29^, we adopted a heterologous promoter strategy to construct the expression cassettes. Due to the limited availability of promoters suitable for *P. patens*, in addition to commonly used plant promoters in this system, we validated the strength of known synthetic plant promoters^30^ using a dual-fluorescence reporter system ^31^ in *P. patens* (Supplementary Fig. 5). Three synthetic plant promoters with higher activity were selected to drive the expression of genes in the paclitaxel biosynthetic pathway (Fig. 4a and Supplementary Fig. 5c). To minimize the number of promoters used and reduce the overall length of the reconstructed synthetic pathway, we introduced a self-cleaving 2A peptide strategy^32–34^ to develop a multi-gene co-expression system (Fig. 4a). To validate the effectiveness of the 2A peptide in *P. patens*, we selected the porcine teschovirus-1 (P2A)^33^ peptide to link TXS with GFP, and also directly fused GFP to the C-terminus of TXS (Supplementary Fig. 6). The cleavage of the P2A peptide in *P. patens* protoplasts was assessed via confocal microscopy (Supplementary Fig. 6). Unlike the chloroplast localization observed for TXS-GFP, in protoplasts transformed with *TXS*-*P2A*-*GFP* construct, most of the GFP fluorescence was distinctly distributed in the cytoplasm, indicating that most of the GFP protein had been cleaved from TXS (Supplementary Fig. 6).

The adoption of the MIT strategy facilitates the systematic, phased verification of intermediate products and enables rapid iterative optimization of the paclitaxel synthetic pathway. We divided the paclitaxel biosynthetic pathway into three parts (Fig. 4a), with the synthesized metabolites progressively leading to taxadiene-5α-ol, baccatin III, and finally paclitaxel. The neutral sites selected for integration were the *PTA*, *MADS*, and *HB7* gene loci^35^ (http://www.nibb.ac.jp/evodevo/PHYSCOmanual/11.6_revised_170727.htm), respectively. The designed fragments were chemically synthesized in segments and assembled using a yeast-based system. Due to the cytotoxicity of some genes in the first half of the paclitaxel biosynthetic pathway in both *E. coli* and *S. cerevisiae*, we employed the low-copy *E. coli-*yeast shuttle plasmid vector for DNA assembly to mitigate the negative effects associated with gene dosage. The three successfully assembled fragments (Supplementary Fig. 7) were used to construct the paclitaxel-engineered line.

### Paclitaxel-producing line construction

We constructed transgenic lines via PEG-mediated transformation. Individual fragments (Part 1, Part 2, and Part 3) were separately transformed and selected on media containing G418, hygromycin (HygR), and zeocin (Zeo) (Fig. 4a), yielding 7, 10, and 15 transgenic lines, respectively. The Part 1 transgenic lines successfully produced taxadiene (*m/z* 272.2) (Fig. 4a, b, and Supplementary Fig. 8), and taxadiene-5α-ol (Fig. 4a, c, and Supplementary Fig. 9), OCT, iso-OCT (*m/z* 288.2) (Fig. 4a, c), as detected by GC-MS. Subsequently, the Part 3 transgenic lines were cultured in medium supplemented with L-phenylalanine and baccatin III and paclitaxel ([HRMS] *m/z* 854.3380 [M+H]^+^ and 876.3203 [M+Na]^+^) was detected via LC-MS analysis (Fig. 4a, e, and Supplementary Fig. 10b).

Based on the Part 1 or Part 2 transgenic lines, additional transformations were performed to integrate Part 2 or Part 1, respectively. Dual-fragment lines containing both Part 1 and Part 2 was successfully obtained, and baccatin III ([HRMS] *m/z* 587.2491 [M+H]^+^ and 609.2306 [M+Na]^+^) was detected by LC-MS (Fig. 4a, d, and Supplementary Fig. 10a). Based on the transformants from part 1/2, further transformation with Part 3 yielded 20 transgenic lines, among which paclitaxel ([HRMS] *m/z* 854.3378 [M+H]^+^ and 876.3209 [M+Na]^+^) was detected by LC-MS (Fig. 4a, e, and Supplementary Fig. 10d), with the highest titer reaching 0.17 μg/g (FW), 0.92 μg/g (DW) in solid culture or 2.41 μg/L in liquid culture.

After more than 4 months and 20 successive subcultures, the engineered line consistently maintained stable production of paclitaxel, demonstrating the successful establishment of a genetically stable paclitaxel biosynthetic line.

### Optimization of paclitaxel- engineered strain

**Fig. 5:**
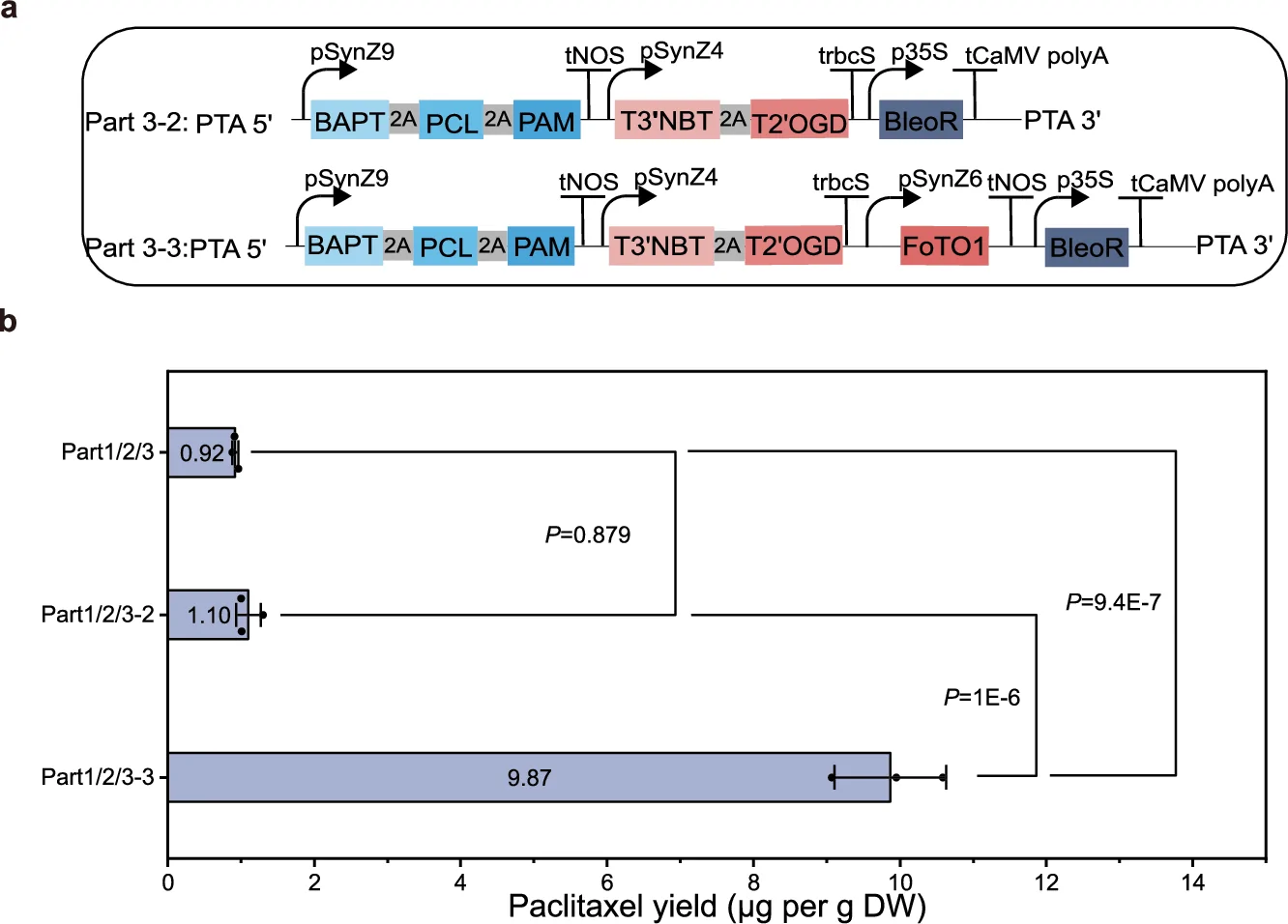
Optimization and detection of the paclitaxel-engineered strain. **a** Schematic diagram of the modification of the third part of the heterologous gene module. Part 3-2 (*BAPT*, *PAM*, *PCL*, *T2*′*OGD* and *T3*′*NBT*). Part 3-3 (*BAPT*, *PAM*, *PCL*, *T2*′*OGD*, *T3*′*NBT*, *FoTO1*). 2A: 2A self-cleaving peptides. Selection markers: *BleoR* (zeocin resistance). **b** Detection of paclitaxel production in stable transgenic lines harboring the corresponding gene modules using QTRAP 6500+ Low Mass spectrometer. Part 1/2/3: transgenic lines carrying fragments Part 1, 2, and 3 were constructed. Part 1/2/3-2: transgenic lines carrying fragments Part 1, 2, and 3-2 were constructed. Part1/2/3-3: transgenic lines carrying fragments Part 1, 2, and 3-3 were constructed. The one-way ANOVA *p*-values were used for significance difference analysis (*p*-values of 0.05 or less indicated statistical significance). Data are mean ± SD (*n* = 3). Data are from biological replicates; each unit is an independent transgenic line. Source data are provided as a Source Data file.

As international research teams have progressively elucidated the biosynthetic pathway of paclitaxel^6,8–12^, various enzymes and facilitators have been successively identified that can enhance the yield of paclitaxel and its precursors, such as T2′OGD and T3′NBT discovered by Liang et al., which can increase the yield of paclitaxel^6^, and FoTO1 discovered by McClune et al., which can enhance the yield of taxadien-5α-ol^11^. We first validated in chassis cell *P. patens* through transient expression that T2′OGD and T3′NBT can increase the yield of paclitaxel (Supplementary Fig. 11). Subsequently, based on yeast recombination, two modifications were made to vector Part 3: first, TB506 and DBTNBT in Part 3 were replaced with T2′OGD and T3′NBT, yielding recombinant vector Part 3-2; second, FoTO1 was added to Part 3-2, yielding recombinant vector Part 3-3 (Fig. 5a). Based on the transformants of Part 1/2 of *P. patens*, Part 1/2/3-2 and Part 1/2/3-3 transformants were constructed respectively. After selection with Zeocin resistance, quantitative analysis was performed using a QTRAP 6500+ Low Mass spectrometer. The results showed that the highest yields of transformants Part 1/2/3-2 and Part 1/2/3-3 reaching 1.10 and 9.90 μg/g (DW), respectively (Fig. 5b). Compared with transformant Part 1/2/3, the yields of transformants Part 1/2/3-2 and Part 1/2/3-3 were increased by approximately 1.2-fold and 10.8-fold, respectively.

## Discussion

The amount of paclitaxel biosynthesis in engineered moss strains reached 1.10 μg/g (FW), 9.90 μg/g (DW) in solid culture or 21.8 μg/L in liquid culture. To date, two relevant studies on paclitaxel biosynthesis have been reported: one achieved a maximum yield of 64.29 ng/g (FW) using a tobacco transient expression system, though not in a stable transgenic line^8^, the other established a semi-synthetic pathway from baccatin III to paclitaxel in yeast, with a yield of 0.97 μg/L^6^. In comparison, the paclitaxel production in our engineered moss system was 17 times higher than that of the tobacco transient system under solid culture conditions, and 22.5 times higher than that of the yeast semi-synthetic system in liquid culture.

The cumulative dosage of paclitaxel in a standard chemotherapy cycle is approximately 3 g. *Taxus cuspidata* is a species of yew with a high paclitaxel content. The bark of 56-year-old *Taxus cuspidata* contains 0.00465% paclitaxel^36^, meaning approximately 64.5 kg of bark is required to obtain 3 g of paclitaxel. However, stripping the bark kills the trees, leading to an unsustainable ecological disaster. In the semi-synthetic approach, 10-deacetylbaccatin III (10-DAB) serves as a precursor for chemical conversion to paclitaxel. *Taxus chinensis* var. *mairei* contains 0.0584% 10-DAB^37^. Under ideal conditions, producing 3 g of paclitaxel requires 8.055 kg of *Taxus chinensis* var. *mairei* leaves and branches. However, actual production efficiency is considerably lower. Currently, using engineered *P. patens* biomass, although 303 kg of engineered biomass (DW) is required to produce 3 g of paclitaxel. Under ideal conditions, the production target can be achieved with just about six 20-ton fermentation tanks, each occupying approximately 2–3 square meters of floor space, operating for 1 week. Moreover, this method does not rely on scarce yew resources at all, offers highly controlled fermentation conditions, and holds significant potential for further optimization to substantially increase yield.

The optimization strategy can be implemented in the following four aspects. Firstly, optimize key rate-limiting steps. Enzymes, such as taxadiene synthase (TXS)^38^, taxadiene-5α-hydroxylase (T5αOH)^39^, and 10-deacetylbaccatin III-10β-O- acetyltransferase (DBAT)^40^, are bottlenecks in the metabolic pathway. Enhancing their catalytic efficiency or expression levels is crucial for effectively increasing the final yield of paclitaxel. The functional gene FoTO1, which significantly improves the catalytic efficiency of enzyme T5αOH, reduces the production of isomers (OCT, iso-OCT), and leads to a marked increase in the yield of taxadien-5α-ol^11^, has been incorporated into the optimization of our engineered strain paclitaxel, and significantly improved the yield of paclitaxel (Fig. 5). Although the regulatory genes responsible for enhancing the catalytic efficiency of the two remaining rate-limiting enzymes have not yet been identified, we can employ genetic engineering approaches to over-express these enzymes, thereby increasing the metabolic flux through the entire pathway. Alternatively, rate-limiting enzymes can be engineered, for example, through rational design or directed evolution based on structural and mechanistic studies to enhance their catalytic efficiency.

Secondly, since the cleavage efficiency of 2A peptides is not 100% (Supplementary Fig. 6), the 2A peptides (Fig. 4a) can be replaced with different strong promoters. This substitution will enhance the independent and controllable expression levels of genes involved in the paclitaxel biosynthesis pathway, enabling fine‑tuned regulation of multigene expression profiles.

Thirdly, to enhance the overall efficiency of the paclitaxel biosynthetic pathway, a key strategy involves screening and introducing high-activity enzyme genes to replace those with lower catalytic efficiency within the pathway. This approach optimizes the catalytic capacity and metabolic flux at specific nodes. For instance, the introduction of T2′OGD and T3′NBT discovered by the Kampranis team will lead to a high yield of paclitaxel^6^. Therefore, we replaced TB506 and DBTNBT with T2′OGD and T3′NBT, which improved the yield of paclitaxel to some extent (Fig. 5). Although there was no significant improvement in *P. patens* as reported by the Kampranis team, which may be caused by the difference of chassis cells. Meanwhile, the yield of baccatin III based on tobacco reported by the Sattely team is equivalent to that of *Taxus*^11^. We will consider introducing the gene set reported by the Sattely team to improve the yield of paclitaxel.

Finally, precursor supply can be enhanced by overexpressing endogenous pathway enzymes, integrating heterologous synthesis modules, or optimizing cofactor availability to elevate intracellular levels of essential precursors, such as GGPP and cofactors, including NADPH^7^. The synergistic integration of these approaches is expected to achieve a significant increase in paclitaxel yield.

Croteau’s team identified a 3′-N-debenzoyl-2′-deoxy-taxol N-benzoyltransferase (DBTNBT) that can catalyze the conversion of N-debenzoyl-2′-deoxy-taxol to 2′-deoxy-taxol^41^. Moyano’s team identified that TB506 is involved in the final hydroxylation step of the paclitaxel biosynthetic pathway through in silico characterization, molecular docking, and a heterologous system using *Pisum sativum* protoplasts^42^. Liang et al. identified T2′OGD and T3′NBT, through a heterologous system utilizing tobacco protoplasts, as direct participants in the final two steps of paclitaxel biosynthesis^6^, and proposed that two enzymes, DBTNBT and TB506, could not produce the target product based on the substrate. We validated the functions of TB506 and DBTNBT in the final steps of paclitaxel biosynthesis within a heterologous system. Paclitaxel could not be detected after removing TB506 (Supplementary Fig. 11). Although the yield of paclitaxel was significantly reduced after removing DBTNBT, paclitaxel could still be detected in a small amount, however, after adding DBTNBT, the yield of paclitaxel was markedly increased (Supplementary Fig. 11), which indicated that both TB506 and DBTNBT were very important for taxol biosynthesis based on *P. patens*, and it was also speculated that *P. patens* contains endogenous enzyme with a function similar to DBTNBT.

Although T2′OGD and T3′NBT did increase paclitaxel production to some extent compared to TB506 and DBTNBT in *P. patens*, no significant doubling effect was detected (Supplementary Fig. 11). This indicates that both TB506 and DBTNBT are functionally involved in the paclitaxel biosynthetic pathway. Previous studies have also shown that overexpressing *DBTNBT* in *Taxus baccata* significantly increased the yield of paclitaxel^43^. Although Liang et al. proposed that the final pathway for paclitaxel synthesis follows the order of benzoylation followed by hydroxylation^6^; however, current results suggest that the DBTNBT/TB506 pathway different from the proposed linear sequence, allowing an alternative order of hydroxylation followed by benzoylation.

Biosynthesis based on cellular chassis holds significant advantages over traditional chemical synthesis in producing complex, high-value natural products, primarily due to the rich natural enzyme resources inherent in cells. Among these, plant chassis demonstrate unique value compared to microbial chassis, such as *E. coli* and yeast: plant systems not only possess functional genes more similar to those of higher plants like *Taxus*, but also provide a cellular and metabolic microenvironment closer to that of natural products. This makes them more conducive to simulating and optimizing the biosynthetic pathways of target compounds. It should be noted that this study does not elucidate the paclitaxel biosynthetic pathway with full mechanistic resolution, and that certain reactions may be catalyzed by the background metabolism of *P. patens*.

It is now widely accepted in the scientific community that paclitaxel biosynthesis is not a simple linear process, but rather a complex and dynamically regulated network composed of multiple parallel and critical catalytic nodes^7,10,13^. With advances in integrated multi-omics technologies, a comprehensive and systematic understanding of the paclitaxel biosynthetic network is anticipated in the near future.

## Methods

### Chemicals and reagents

The taxadiene reference standard was purified from engineered *E. coli* strains harboring the *TXS* gene. Baccatin III (Sigma, Cat. No. B8154); paclitaxel (Sigma, Cat. No. T7402) and L-phenylalanine (Sigma, Cat. No. P2126) were purchased commercially.

### Plant cultivation

The Gransden 2004 wild-type strain of *Physcomitrium patens* subsp. Patens^44^ was maintained through protonemal culture on solid BCDATG agar medium (1 mM MgSO_4_, 10 mM KNO_3_, 45 μM FeSO_4_, 1.84 mM KH_2_PO_4_ [adjust the pH to 6.5 with KOH], and trace element solution [0.22 μM CuSO_4_, 0.19 μM ZnSO_4_, 10 μM H_3_BO_3_, 0.10 μM Na_2_MoO_4_, 2 μM MnCl_2_, 0.23 CoCl_2_, and 0.17 μM KI], 1 mM CaCl_2_, 5 mM diammonium (+)-tartrate, 0.5% (w/v) glucose, and solidified with 0.8% (w/v) agar) under controlled conditions (22 °C with a 16/8 h light/dark photoperiod)^45^.

### Transient expression plasmid construction

Vector pTEV was used for transient gene expression in *P. patens*. The gene sequences were codon-optimized for *P. patens* and chemically synthesized by General Biosystems (Anhui) Co., Ltd. The codon optimization was performed using the GeneOptimizer Algorithm^46^. The algorithm intelligently balances multiple parameters, including codon usage preference, secondary structure minimization, elimination of repetitive sequences and cryptic splice sites, removal of restriction enzyme cleavage sites, GC content control, design of homologous recombination regions, and eradication of detrimental motifs. Consequently, the codon composition of the target sequence was optimized for expression in *P. patens*.

Expression vectors were constructed by inserting the p35S promoter and gene of interest using Gibson assembly. All gene sequences used in this study were referenced from previously published research (Supplementary Data 1)^6,8,11^.

### Isolation of protoplasts

To isolate protoplasts, we submerged 5-day-old protonemata of *P. patens* in an enzyme solution composed of 0.8% (w/v) Driselase and 8% (w/v) mannitol for 30 min. After diluting the mixture with 8% mannitol, we filtered it through a 100-µm nylon mesh. The filtrate was collected by centrifugation at 180 × *g* for 3 min in a 50 ml round-bottomed tube (Thermo Fisher), and the pellet was resuspended in 8% mannitol and spun again to harvest the protoplasts^22,47^.

### Transient gene expression in *P. patens*

The isolation protocol involved resuspending freshly harvested protoplasts in MMM solution (0.1% MES, pH 5.6; 0.5 M mannitol, 15 mM MgCl_2_) to achieve a final density of 1.6 × 10^6^ cells/ml. For transformation, 300 µl of protoplast suspension was carefully combined with 20 µg plasmid DNA (in <30 µl volume) and 330 µl PEG solution [40% (w/v) PEG-6000, 0.4 M mannitol, 100 mM Tris buffer (pH 8.0), 100 mM Ca(NO_3_)_2_], followed by 30 min incubation. Post-incubation, the protoplasts underwent washing with 1.5 ml MMM solution. After centrifugation at 180 × *g* for 3 min, discard the supernatant, resuspend the protoplasts with 200 μl MMM, transfer to a cell culture dish, and culture at 22 °C in the dark for 48 h.

### Confocal laser scanning microscopy

Transiently transfected protoplasts were plated on specialized confocal imaging dishes (Cellvis, Cat. No. 35-20-1.5-N). After culturing for 1 day, fluorescence microscopy was performed directly using a confocal microscope (ZEISS Elyra 7-LSM980 module). Cell imaging was conducted at room temperature using a 63× oil immersion objective. For green fluorescent protein (GFP) imaging, fluorescence was excited with a 488 nm laser line, and emission was collected within the 500–550 nm wavelength range. Chloroplast autofluorescence was simultaneously captured using the same excitation parameters, with emission signals collected in the 650–700 nm range. Fluorescence microscopy images were processed and analyzed using ZEN 3.1(blue edition) software.

### Dual-fluorescence reporter construction

The β-glucuronidase (*GUS*) gene was linked to the AtUBI promoter, and the *LUC* gene was connected to the promoter to be tested. All synthetic promoters and mini CaMV35S promoter were chemically synthesized by General Biosystems (Anhui) Co., Ltd, and a dual-fluorescence reporter vector was constructed using seamless cloning.

### Luciferase assay

The frozen protoplasts were resuspended in 50 µl of lysis buffer containing 2.5 mM Tris-phosphate (pH 7.8), 1 mM DTT, 2 mM DACTAA, 10% (v/v) glycerol, and 1% (v/v) Triton X-100. A 20 µl aliquot of the protoplast lysate was mixed with 100 µl of LUC assay reagent (Promega, E1500), and luminescence from luciferase (LUC) was measured using a Varioskan flash (Thermo Scientific) plate reader.

### GUS assay

A 2 µl of protoplast lysate was combined with 10 µl of 4-methylumbelliferyl β-D-glucuronide (MUG; Gold Biotechnology) substrate solution, which contained 10 mM Tris-HCl (pH 8.0), 1 mM MUG, and 2 mM MgCl_2_. After incubation at 37 °C for 30 min, the reaction was terminated by adding 100 µl of 0.2 M Na_2_CO_3_. GUS activity was determined using a plate reader with an excitation wavelength of 355 nm and an emission wavelength of 460 nm.

### Large-fragment DNA assembly

The paclitaxel biosynthetic pathway was engineered as five large DNA fragments, each flanked by restriction sites. Within each fragment, a single promoter drove the expression of multiple genes connected via 2A peptides^48^. The designed constructs were then divided into multiple 3 kb segments for chemical synthesis, with 150 bp homologous overlaps incorporated between adjacent fragments. Following synthesis, the 3-kb fragments were assembled into a PCC1 vector using *S. cerevisiae*, following the protocol provided in the Yeast Assembly Kit (Cat. No. MF658-01, Beijing Polymerase Biotechnology Co., Ltd).

### Stable genetic transformation in *P. patens*

The target plasmid was extracted from *E. coli* using a plasmid extraction kit (Cat. No. EM123-01, TransGen Biotech, China) and subsequently linearized by digestion with the restriction enzyme BsiWI (NEB, Cat. No. R3553L), NotI,(NEB, Cat. No. R3189L),, or KpnI (NEB, Cat. No. R0142L), respectively.

Freshly harvested protoplasts were resuspended in MMM solution (0.1% MES, pH 5.6; 0.5 M mannitol, 15 mM MgCl_2_) to obtain a final cell density of 1.6 × 10^6^ cells/ml. For transformation, 300 µl of protoplast suspension was mixed gently with 20 µg plasmid DNA (dissolved in less than 30 µl) and 330 µl PEG solution (40% (w/v) PEG-6000, 0.4 M mannitol, 100 mM Tris buffer [pH 8.0], and 100 mM Ca(NO_3_)_2_). The mixture was incubated for 30 min. After incubation, the protoplasts were washed with 5 ml of W5 solution (13.33 mM MES, pH 5.7, 33.33 mM KCl, 833.33 mM CaCl_2_, 1026.67 mM NaCl). Folloeing centrifugation at 180 × *g* for 3 min, the supernatant was discarded, and the protoplast pellet was resuspended in 8 ml of PRMT medium (1.84 mM KH_2_PO_4,_ 1 mM MgSO_4_·7H_2_O, 40 mM KOH, 10 mM KNO_3_, 45 µM FeSO_4_·7H_2_O, 5.0 mM ammonium tartrate, 0.22 µM CuSO_4_·5H_2_O, 10 µM H_3_BO_3_, 0.23 µM CoCl_2_·6H_2_O, 0.19 µM ZnSO_4_·7H_2_O, 2 µM MnCl_2_·4H_2_O, 0.17 µM KI, 0.1 µM Na_2_MoO_4_·2H_2_O, 0.48 M mannitol, 10 mM CaCl_2_·2H_2_O 0.3% glucose, and 10 mM 0.6% Agar), transferred to 4 petri dish with PRMB medium (1.84 mM KH_2_PO_4_, 1 mM MgSO_4_·7H_2_O, 40 mM KOH, 10 mM KNO_3_, 45 µM FeSO_4_·7H_2_O, 5.0 mM ammonium tartrate, 0.22 µM CuSO_4_·5H_2_O, 10 µM H_3_BO_3_, 0.23 µM CoCl_2_·6H_2_O, 0.19 µM ZnSO_4_·7H_2_O, 2 µM MnCl_2_·4H_2_O, 0.17 µM KI, 0.1 µM Na_2_MoO_4_·2H_2_O, 10 mM CaCl_2_·2H_2_O, 0.48 M mannitol, 0.3% glucose, and 0.8% agar), and culture at 22 °C with a 16/8 h light/dark photoperiod^49^. The transformed protoplasts were screened on solid medium supplemented with the corresponding antibiotic (hygromycin 30 μg/ml, G418 25 μg/ml, or zeocin 100 μg/ml). The selected transformants were cultured on BCD solid medium (1 mM MgSO_4_, 10 mM KNO_3_, 45 μM FeSO_4_, and 1.8 mM KH_2_PO_4_ [adjust the pH to 6.5 with KOH], and trace element solution [0.22 μM CuSO_4_, 0.19 μM ZnSO_4_, 10 μM H_3_BO_3_, 0.10 μM Na_2_MoO_4_, 2 μM MnCl_2_, 0.23 CoCl_2_, and 0.17 μM KI]) for subsequent analysis^45^.

### Junction PCR analysis

The target large-fragment DNA was divided into multiple 3 kb fragments for chemical synthesis, which were then assembled into a complete large fragment via yeast homologous recombination technology. The strains are cultured in a nutrient-deficient medium^50^. To verify correct assembly, we designed junction PCR primers at each adjacent fragment junction, with all primers spanning the boundaries between two fragments (Supplementary Data 2). For PCR analysis, *TransStart Taq* DNA polymerase was applied (TransGen Biotech [Beijing] Co. Ltd, China). PCR was performed as follows: 94 °C for 2 min; 32 cycles of 94 °C for 30 s, annealing (based on primer Tm) for 30 s, 72 °C extension (1–2 min/kb); final extension 72 °C for 10 min^51^.

### GC-MS

For GC-MS analysis of taxadiene or taxadiene-5α-ol, the transiently transfected cell material (stably transformed strains were first ground in liquid nitrogen, three biological replicates were set for each sample group) was thawed from −80 °C storage, mixed with 1–3 ml n-hexane, and homogenized by pipetting^52^. Samples were immersed in an ice-water slurry and subjected to ultrasonic extraction in pulsed mode (4 s on, 5 s off) for 20 min at 120 W power. After centrifugation at 400 × *g* for 5 min, the organic extract was collected and concentrated under a vacuum drying oven, filtered through a 0.22 μm polytetrafluoroethylene (PTFE) syringe filter, and transferred to a chromatography vial for subsequent analysis. Samples were purified by high‑performance liquid chromatography (HPLC) prior to analysis. A 1 μl aliquot was injected in splitless mode into an Agilent 7890B-5977B gas chromatograph equipped with an Agilent J&W HP-5ms capillary column, and the separation was carried out on the same column under splitless conditions. Ultra-high-purity helium was used as the carrier gas at a flow rate of 1.0 mL/min. The oven temperature was held at 80 °C for 2 min, then increased at 10 °C/min to 200 °C, and finally held for 10 min. The injector and transfer line temperatures were set at 200 and 250 °C, respectively. The column effluent was ionized by electron impact ionization at 70 eV, and mass spectra were acquired over the range *m/z* 30–300. MassHunter software was used to extract mass, retention time, and peak intensities from the raw data files. Metabolites were identified and annotated by comparison retention times and characteristics with authentic standards and predicted monoisotopic masses. MSD ChemStation/MS Acquisition B.07.05.2479 was used to collect GC-MS data, and MassHunter Qualitative Analysis B.07.00 was used to analyze the results.

### UPLC-qTOF-MS analysis

The ultra-performance liquid chromatography-quadrupole-time of flight-mass spectrometry (UPLC-qTOF-MS) (xevo G2-XS Qtof, England) workflow, consisting of a Waters Acquity UPLC coupled to an Exactive Orbitrap mass spectrometer, was performed as follows: plant samples (three biological replicates were set for each sample group) were ground to powder in liquid nitrogen and extracted with methanol. The organic phase was dried using a vacuum concentrator, filtered through a 0.22 μm PTFE syringe filter, and transferred to a glass vial for analysis. Samples were purified by HPLC prior to analysis. Separation was achieved on a Waters HSS T3 C18 column (100 × 2.1 mm ID, 1.8 μm) at 40 °C using 0.1% formic acid in water (mobile phase A) and 0.1% formic acid in acetonitrile (mobile phase B) at 400 μl/min flow rate with 2 μl injection volume. Mass spectrometry was conducted on an Xevo G2-XS Q-TOF (Waters Corporation) with HESI source in positive full-scan mode (*m/z* 100–1500), using the following parameters: 25,000 resolution, 250 ms maximum scan time, sheath/auxiliary gas flows at 60/35 arb, capillary temperature 150 °C, source heater 300 °C, spray voltage 3 kV, capillary voltage 25 V, and skimmer voltage 15 V. Data were acquired over the 0–15 min chromatographic window, processed using Xcalibur software for mass/retention time/peak intensity extraction, and metabolites were identified by comparison with standards and predicted monoisotopic masses^53,54^. The MASSLynx V4.2 SCN997 software was used to collect and analyze the results.

### QTRAP 6500+ Low Mass

The quantitative method for paclitaxel and its precursor metabolites using a QTRAP 6500 triple-quadrupole mass spec trometer (SCIEX) (QTRAP6500+, America)^55^. Three biological replicates were set for each sample group. Data were acquired using Analyst software (V1.7) and processed for quantitation with SCIEX Multiquant (V3.0.3). The yields of baccatin III and paclitaxel were quantitatively determined by monitoring both their precursor and fragment ions (constituting two characteristic ion pairs). Standard solutions were sequentially diluted into six concentration gradients to establish a linear calibration curve based on the peak areas measured by the system. The specific concentrations of the samples were then calculated by comparing their detected peak areas against the calibration curve.

### High-performance liquid chromatography

In order to obtain high‑purity target metabolites, normal‑phase or reversed‑phase HPLC (LC-2030C 3D, SHIMADZU, Japan) can be selected according to the polarity of the target compounds. For taxadiene or taxadiene-5α-ol, normal‑phase HPLC is chosen, using a silica gel column and isocratic elution with n‑hexane–ethyl acetate as the mobile phase. For baccatin III or paclitaxel, reversed‑phase HPLC is chosen, using a C18 column and isocratic elution with methanol–acetonitrile as the mobile phase system. The elution peak time of a reference standard is used to locate and collect the sample peak within that time window. During collection, the standard is first injected to determine its retention time; then, centered on this retention time, the corresponding effluent fraction is collected by cutting according to the peak width. After pooling the fractions, the concentration affords the purified product.

### Data analysis and illustrations

InDraw v6.2.3 was used to draw chemical structures. Microsoft Excel 2016 and Origin 2025 were used for bar charts, and Adobe Illustrator and Microsoft PowerPoint were used for the preparation of illustrations.

### Statistics and reproducibility

No statistical method was used to predetermine sample size. No data were excluded from the analyses. The experiments were not randomized. The investigators were not blinded to allocation during experiments and outcome assessment.

### Reporting summary

Further information on research design is available in the Nature Portfolio Reporting Summary linked to this article.

## Supplementary information

Supplementary Information

Peer Review File

Description of Additional Supplementary Files

Supplementary Data 1

Supplementary Data 2

Reporting Summary

## Source data

Source Data

Source data

## Data Availability

The data generated in this study are provided in the Supplementary Information/Source Data file. The ID numbers of all enzymes used in this study are from GenBank and are listed in Supplementary Data 1. All DNA plasmid sequences used in this study are provided in the Source Data as Snapgene files. Source data are provided with this paper.
