# Supplementary material for: Engineering the paclitaxel biosynthetic pathway in Physcomitrium patens for de novo and stable biosynthesis
Source: Nat Commun. 2026 Oct 21;17:10648. doi: 10.1038/s41467-026-76625-x (PMC13687077; doi:10.1038/s41467-026-76625-x)
Supplement: Supplementary file 1 [file 41467_2026_76625_MOESM1_ESM.pdf]

Engineering the Paclitaxel Biosynthetic Pathway in *Physcomitrium patens* for De Novo and Stable Biosynthesis

Zhao *et al.*

Supplementary Information is available for this paper.

Supplementary Figures

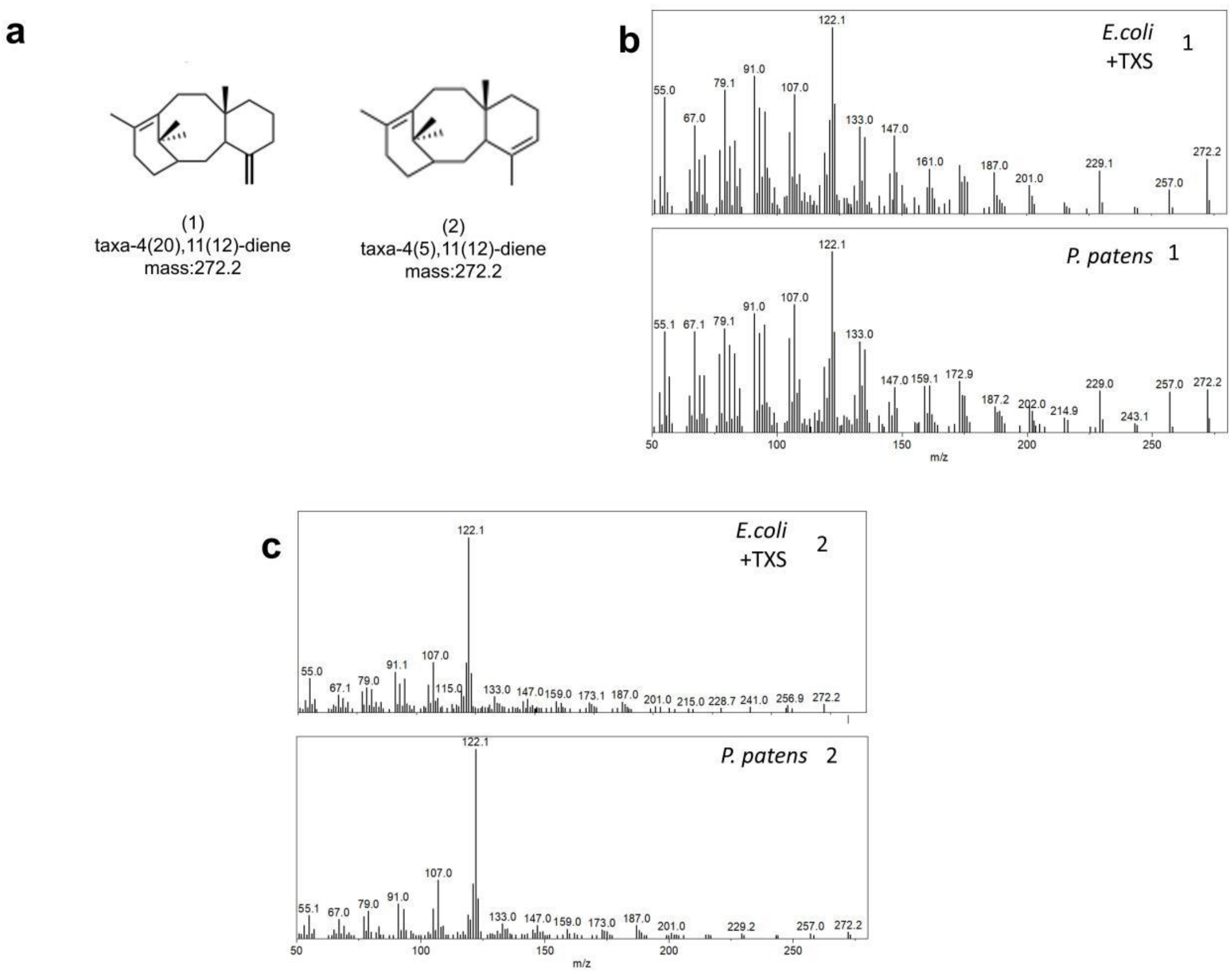

Supplementary Fig. 1|Metabolite analysis of *P. patens* by GC–MS.

(a)The structural formula of taxadiene-4(20), 11(12)-diene, taxadiene-4(5), 11(12)-diene. (b, c) In protoplasts transiently overexpressing *HMGR*, *GGPPS*, and *TXS* (with transit peptide) or *HMGR*, *GGPPS-ΔCTP*, and *TXS-ΔCTP* (without transit peptide), the production of taxadiene and its derivatives were detected by mass spectrometry, compared with the product of *TXS* expressed in *E. coli*. (b) Taxadiene-4(20), 11(12)-diene. (c) Taxadiene-4(5), 11(12)-diene. Source data are provided as a Source Data file.

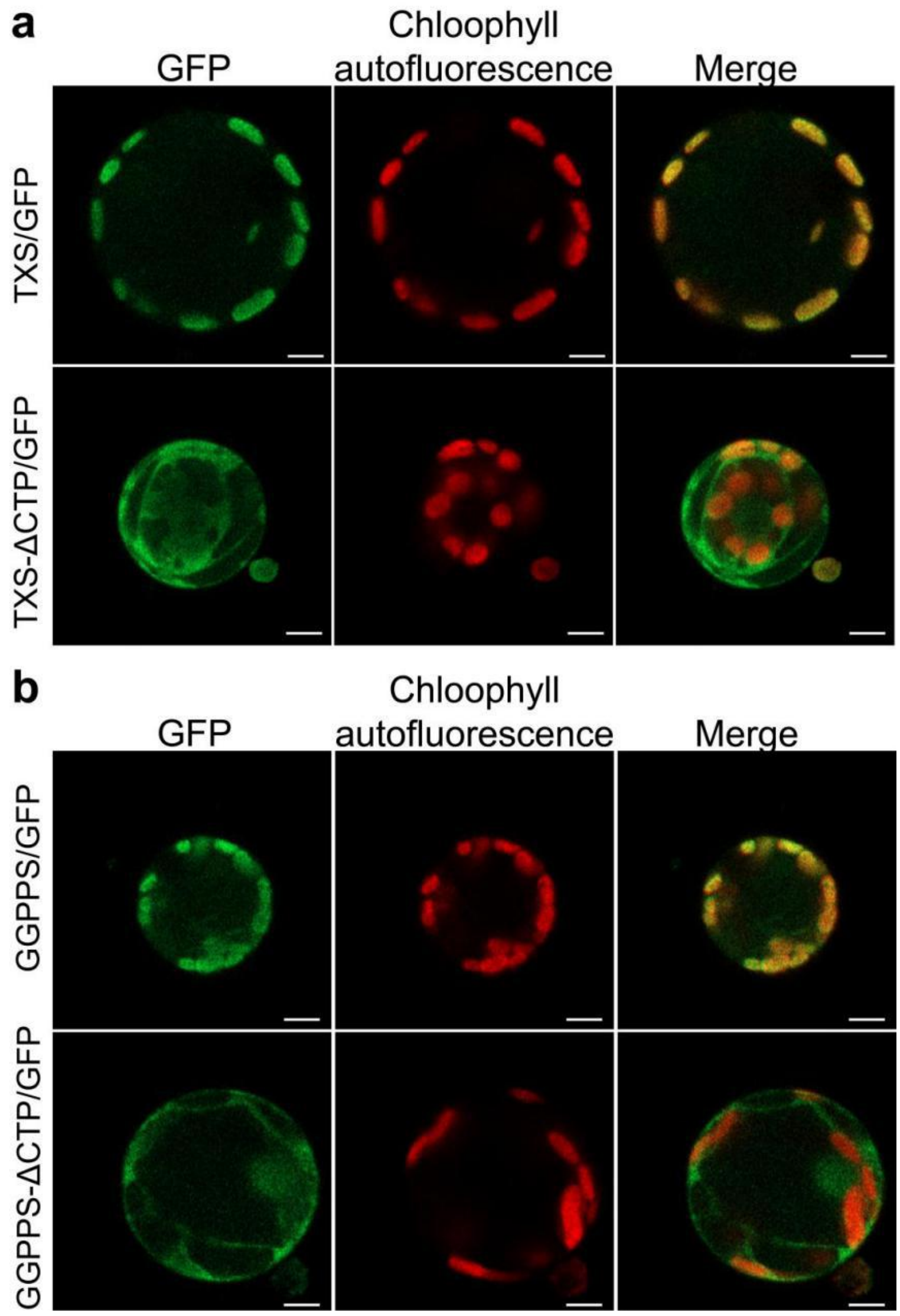

Supplementary Fig. 2|Subcellular localization and targeting of heterologous proteins.

Fluorescence labeling of the fusion proteins: **(**a) TXS-/GFP, TXS-ΔCTP/GFP; (b) GGPPS-/GFP, GGPPS-ΔCTP/GFP. GFP was fused to the C-terminus of the target protein, and individual plasmids were introduced into *P. patens* protoplasts via PEG-mediated transformation. Protoplasts were harvested 1 day after transformation and observed using confocal microscopy. Left to right: GFP channel, Chlorophyll autofluorescence, and merged images. Scale bars: 5 μm. All images shown in this

Figure were derived from three independent biological replicate experiments, and all replicates yielded consistent results. Source data are provided as a Source Data file.

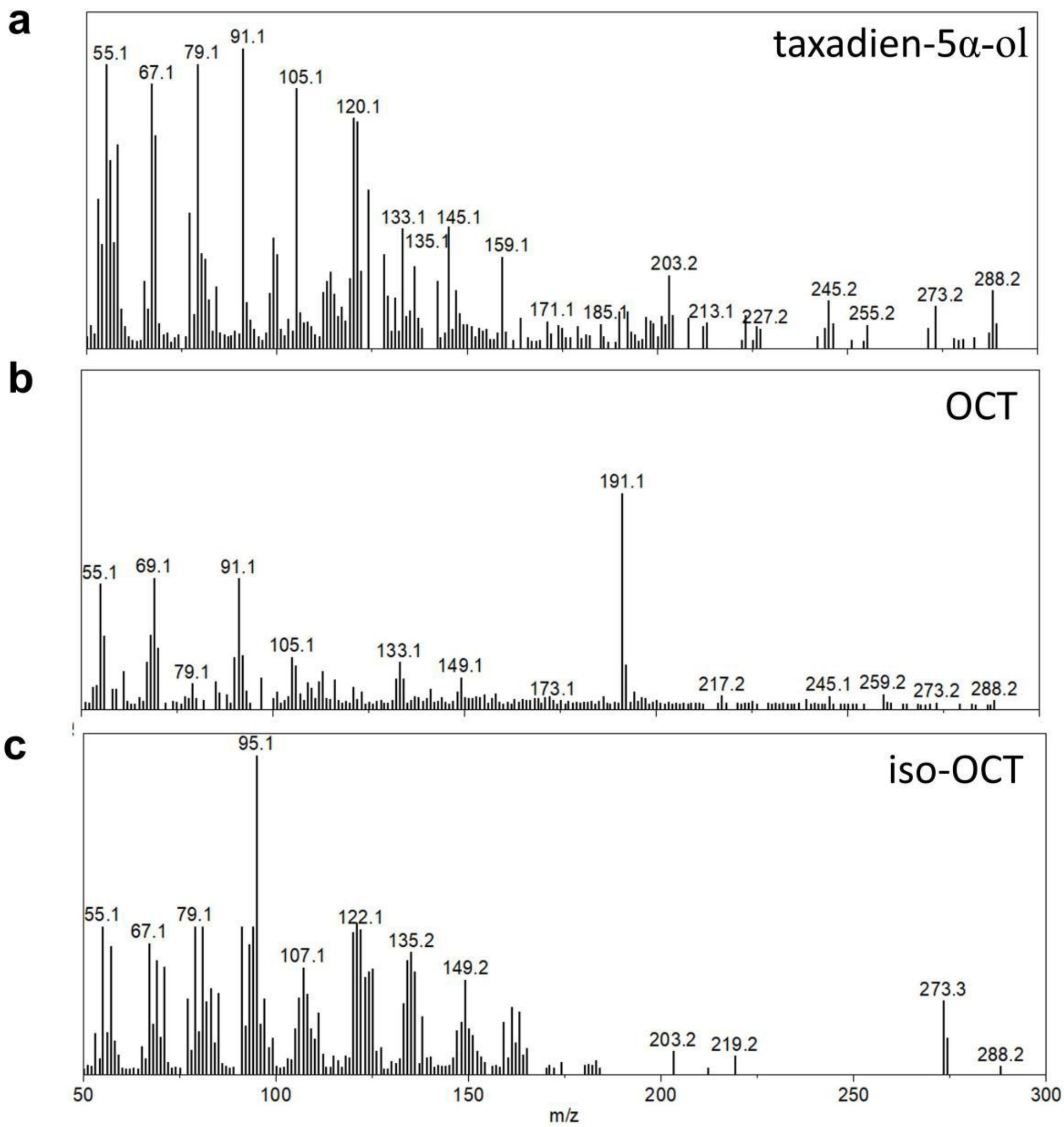

Supplementary Fig. 3|Metabolite analysis of *P. patens* by GC–MS.

In protoplasts transiently overexpressing *HMGR*, *GGPPS-ΔCTP*, *TXS-ΔCTP* (without transit peptide), and *T5αOH*, the production of taxadiene-5α-ol, OCT and iso-OCT were detected by mass spectrometry. (a) Taxadiene-5α-ol. (b) OCT. (c) Iso-OCT. Source data are provided as a Source Data file.

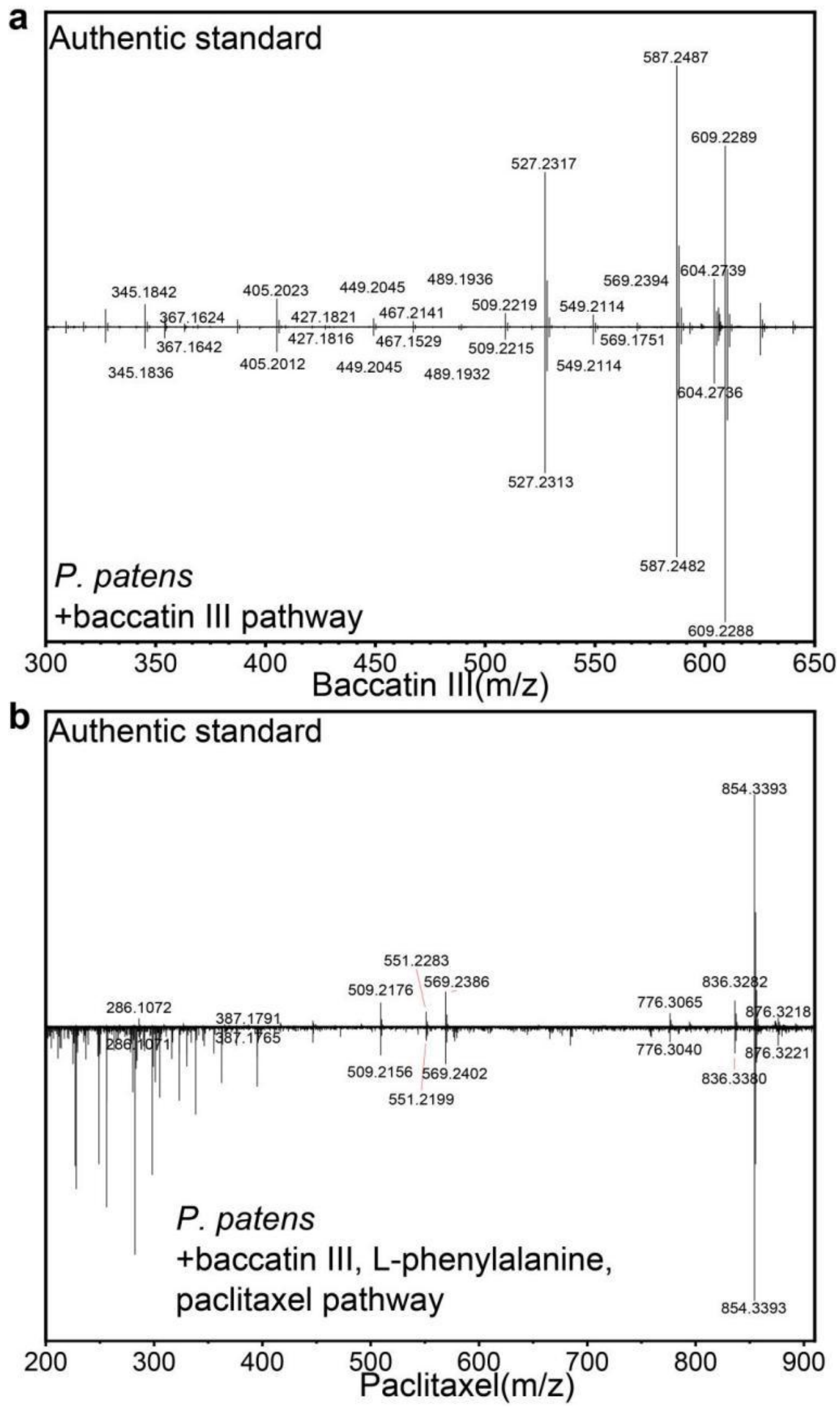

Supplementary Fig. 4|Metabolite analysis of *P. patens* by UPLC-qTOF-MS.

(a) Mass spectrum (MS fragmentation $[M+H]^+$=587.248 Da and MS fragmentation $[M+Na]^+$=609.228 Da) of baccatin III detected in protoplasts transiently expressing the baccatin III biosynthetic pathway, compared to authentic standard. (b) Mass spectrum (MS fragmentation $[M+H]^+$=854.339 Da and MS fragmentation $[M+Na]^+$=876.322 Da) of paclitaxel detected in protoplasts transiently expressing the

complete paclitaxel biosynthetic pathway, compared to authentic standard. Source data are provided as a Source Data file.

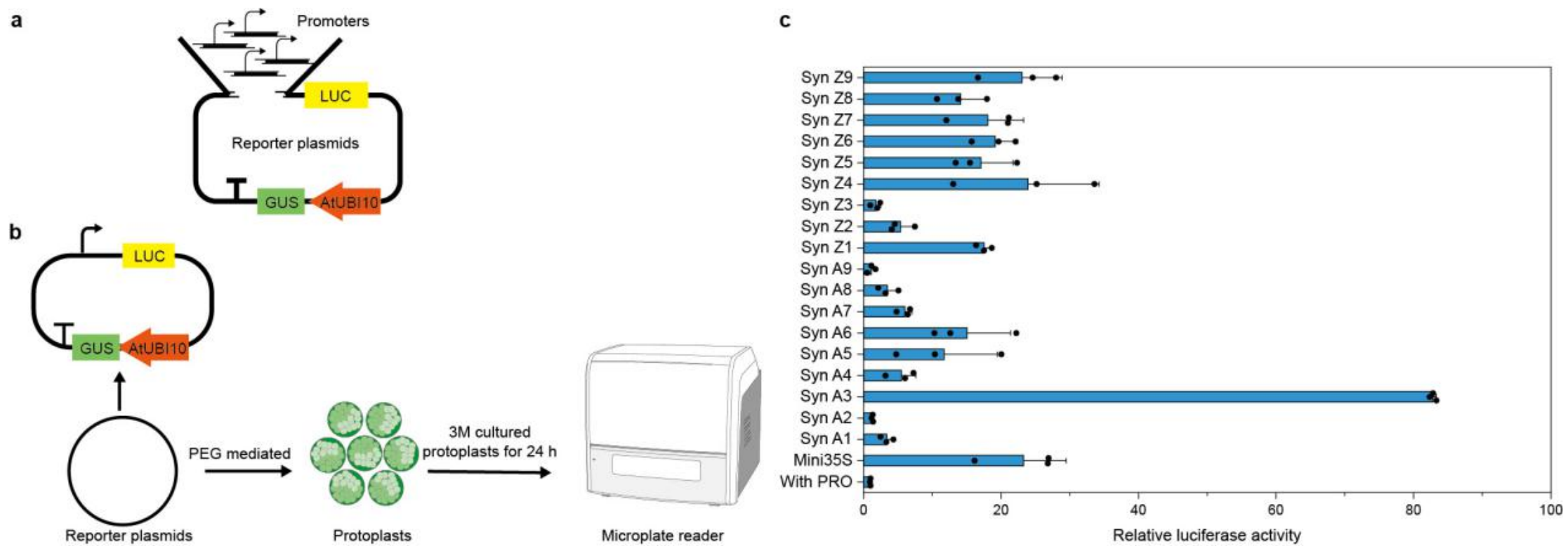

Supplementary Fig. 5|Dual-luciferase assay for evaluating promoter activity in *P. patens*.

(a) Schematic diagram of the dual-luciferase reporter system. GUS, β-glucuronidase; LUC, firefly luciferase. The *GUS* and *LUC* reporter genes are driven by the AtUBI10 promoter and the promoter to be validated, respectively. (b) Illustration of the PEG-mediated protoplast transformation and luminescence detection using a microplate reader. (c) Relative luciferase activity driven by various synthetic promoters (SynA1-SynA9, SynZ1-SynZ9), the minimal 35S promoter (mini35S), and a promoter-less construct (Without PRO). Data are mean ± SD (n = 3). All data in this study are derived from biological replicates, with each experimental unit defined as an independent transformation group. Source data are provided as a Source Data file.

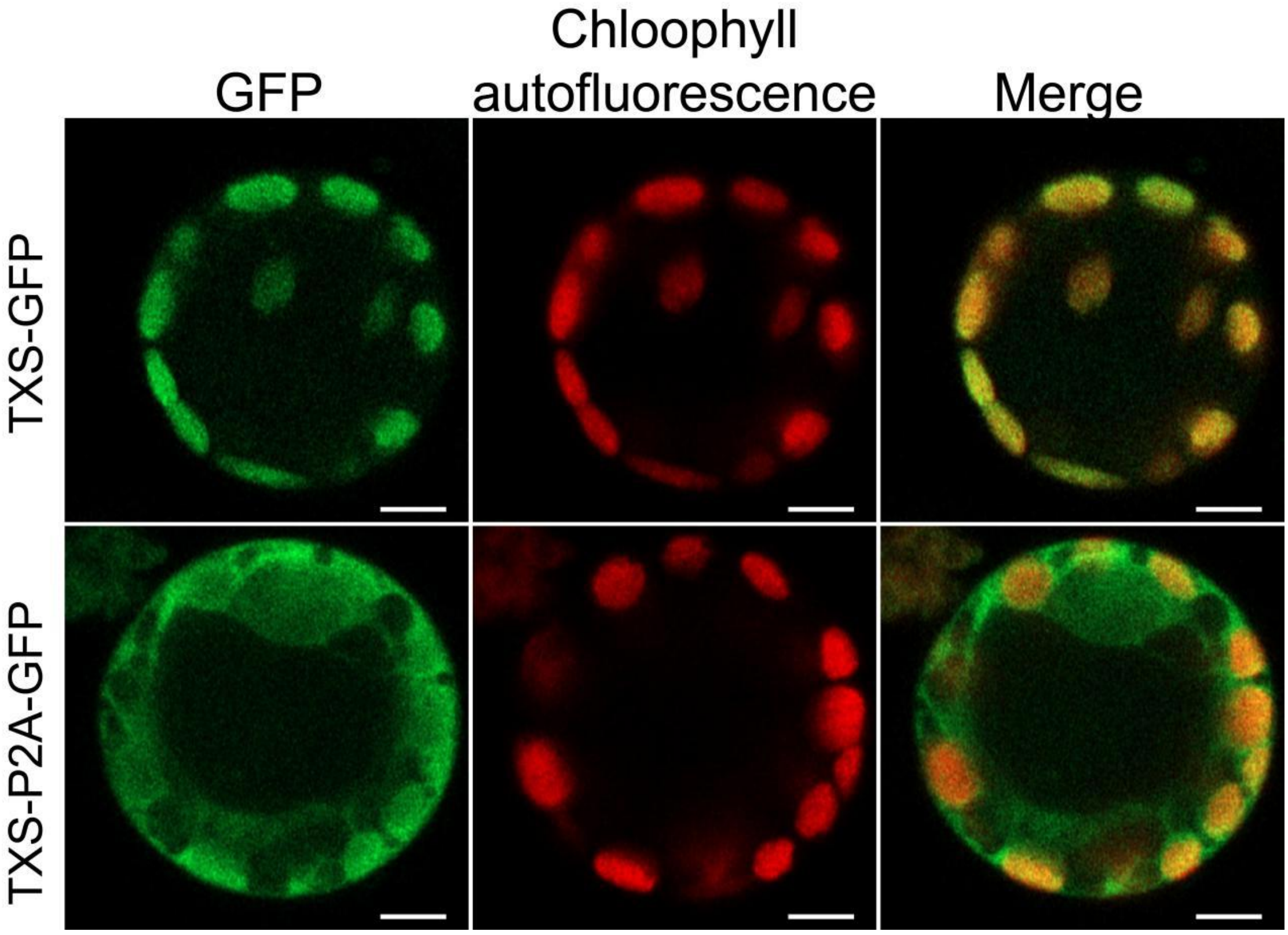

Supplementary Fig. 6|Subcellular localization of TXS-GFP and TXS-P2A-GFP in *P. patens* protoplasts.

TXS-GFP and *TXS-P2A-GFP* were driven by the CaMV 35S promoter and terminated by the NOS terminator. TXS-GFP: GFP was fused to the C-terminus of TXS. TXS-P2A-GFP: the TXS and GFP were linked via P2A. Individual plasmids were introduced into *P. patens* protoplasts via PEG-mediated transformation. Protoplasts were harvested 1 day after transformation and observed using confocal microscopy. From left to right: GFP channel, chloroplast autofluorescence, and merged images. Scale bars: 5 μm. All images shown in this figure are representative of three independent biological replicates, all of which gave similar results. Source data are provided as a Source Data file.

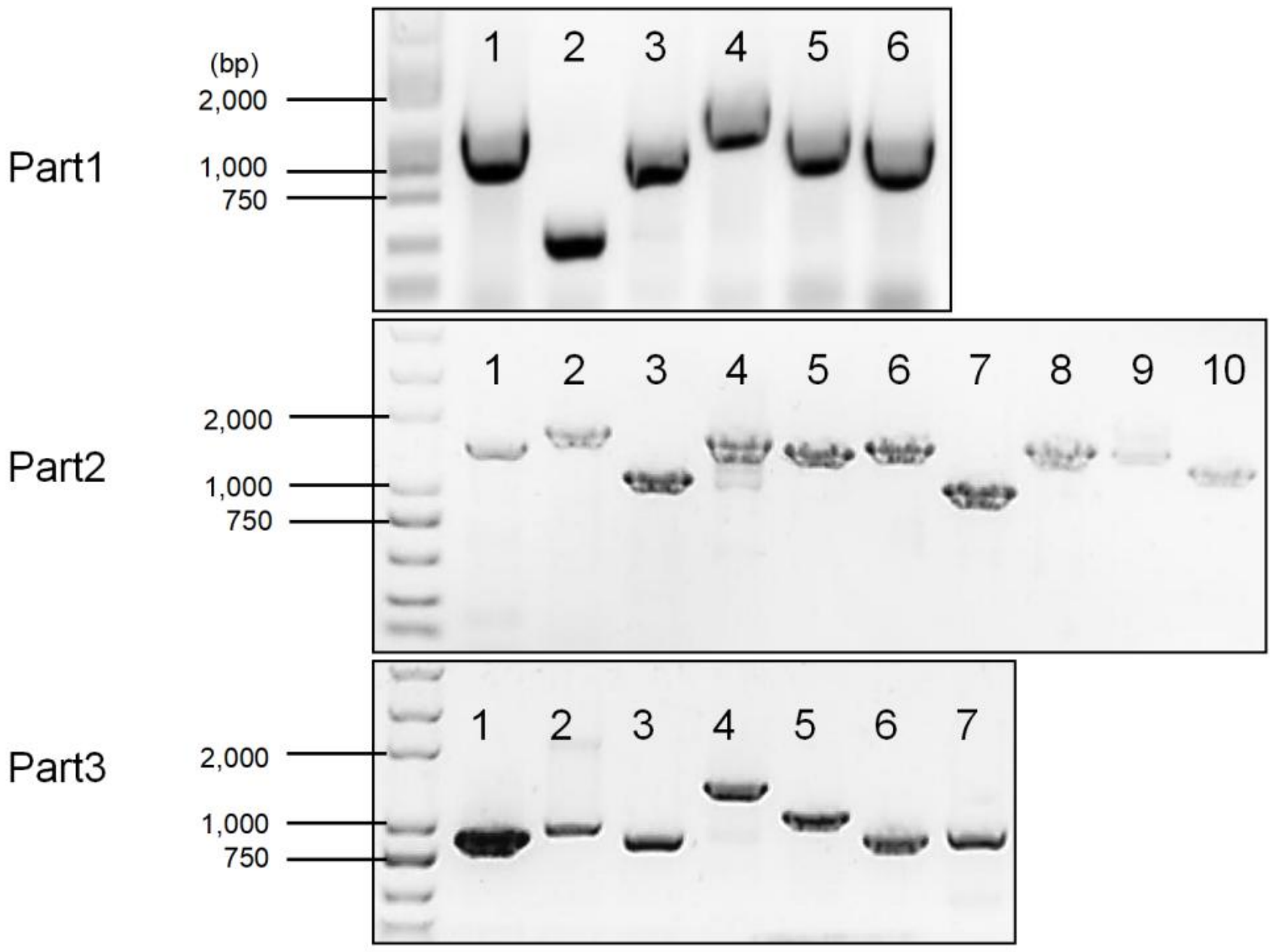

Supplementary Fig. 7|Junction PCR analysis of Part1, 2 and 3.

Part 1, 2, and 3 were tested for 6, 10, and 7 junction sites, respectively, including the connection sites between each pair of 3 kb chemically synthesized fragments as well as the junctions between the fragments and the flanking backbone sequences. All junctions were successfully amplified, producing target bands consistent with the expected sizes. Three independent biological replicates were performed for each transgenic line, and the results were consistent across all replicates. All images shown in this figure are representative of three independent biological replicates, all of which gave similar results. Source data are provided as a Source Data file.

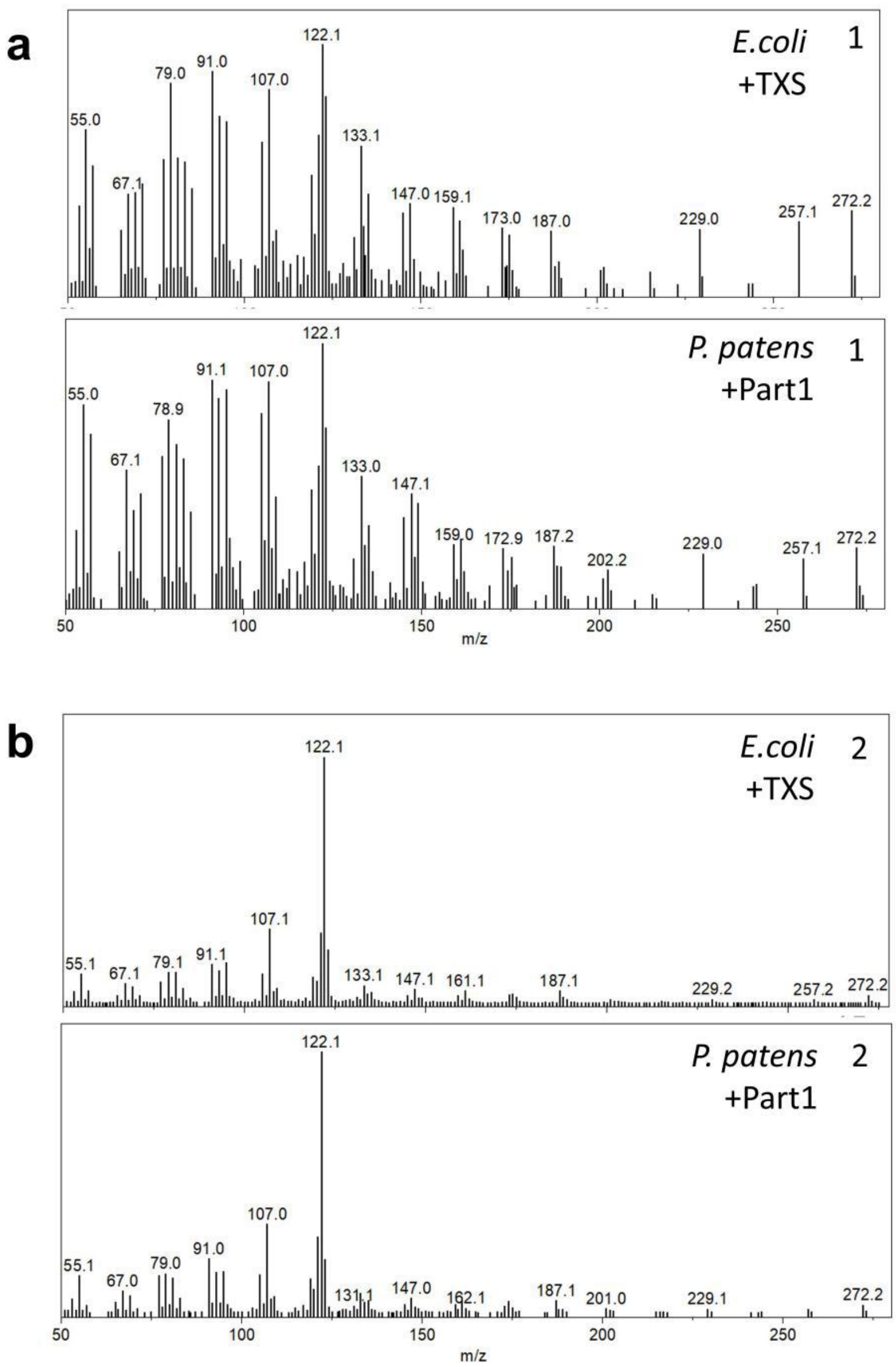

Supplementary Fig. 8|Metabolite analysis of stable transgenic *P. patens* strains by GC–MS.

Mass spectrum of taxadiene detected in Part 1 stable transgenic lines, compared to *E. coli* +TXS. (a) Taxadiene-4(20), 11(12)-diene. (b) Taxadiene-4(5), 11(12)-diene. Source data are provided as a Source Data file.

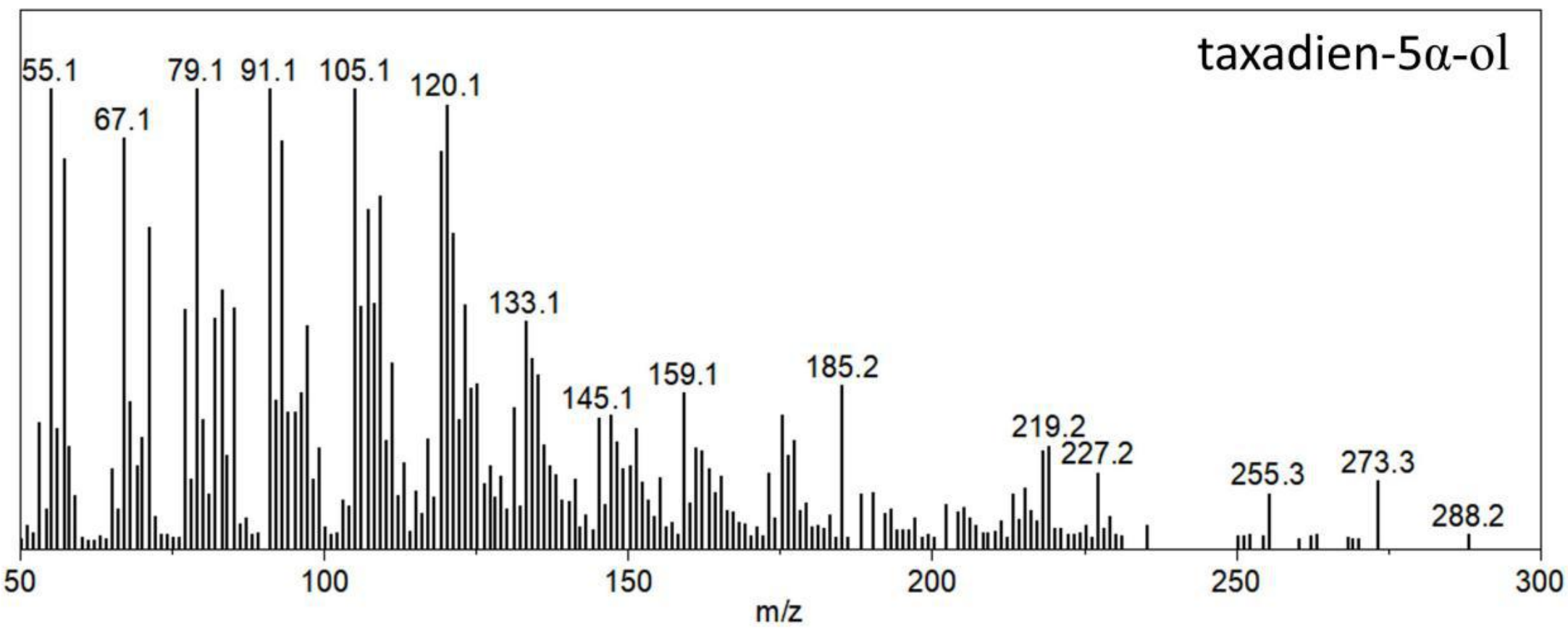

Supplementary Fig. 9|Mass spectrum of taxadien-5α-ol detected in Part 1 stable transgenic lines by GC-MS. Source data are provided as a Source Data file.

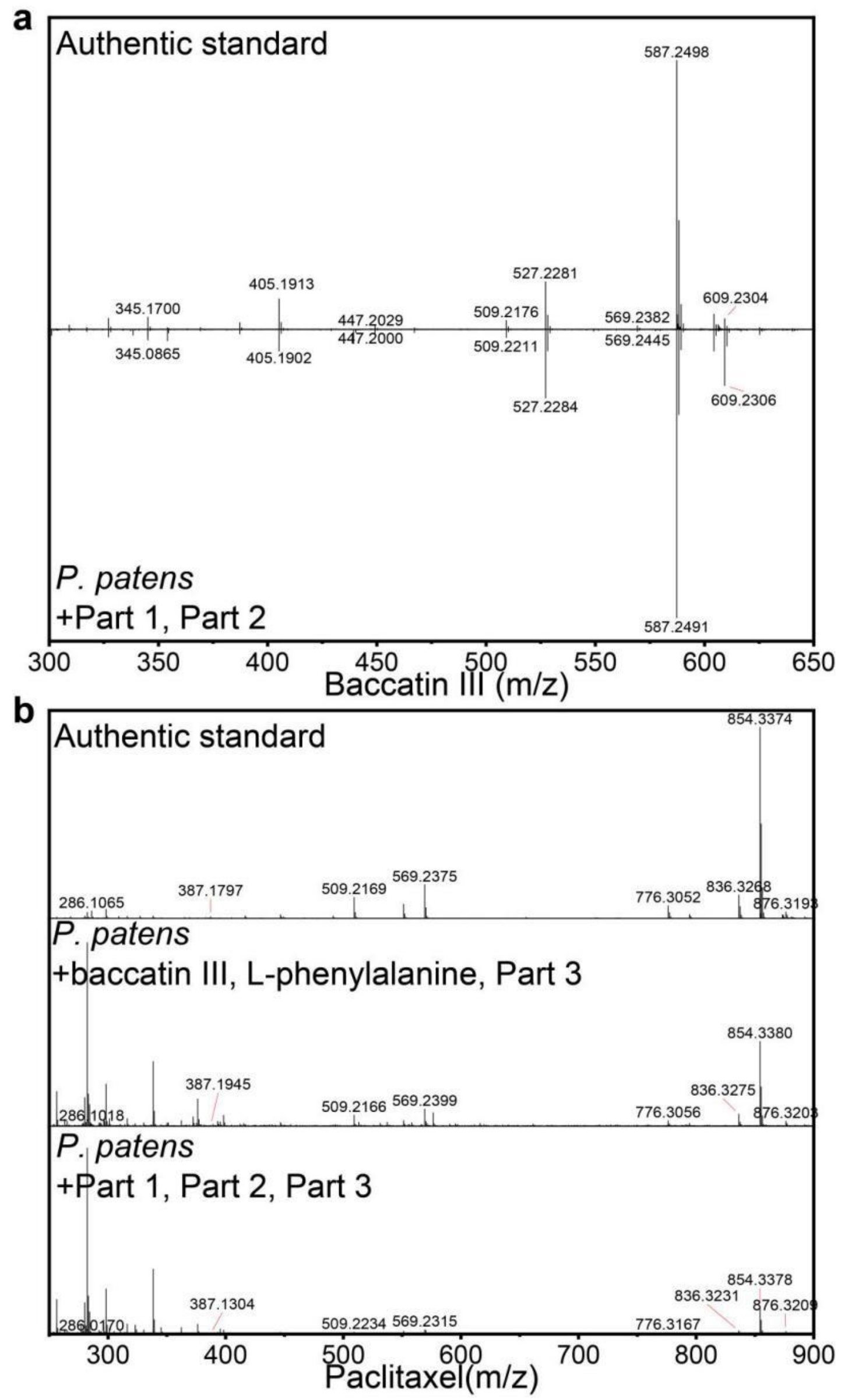

Supplementary Fig. 10|Metabolite analysis of stable transgenic *P. patens* strains by UPLC-qTOF-MS.

(a) Mass spectrum (MS fragmentation $[M+H]^+$=587.249 Da and MS fragmentation $[M+Na]^+$=609.230 Da) of baccatin III detected in Part 1/2 stable transgenic lines, compared to authentic standard. (b) Mass spectrum (MS fragmentation $[M+H]^+$=854.337 Da and MS fragmentation $[M+Na]^+$=876.319 Da) of paclitaxel detected in Part 3 and Part 1/2/3 stable transgenic lines, compared to authentic standard. n.d., not detected. Source data are provided as a Source Data file.

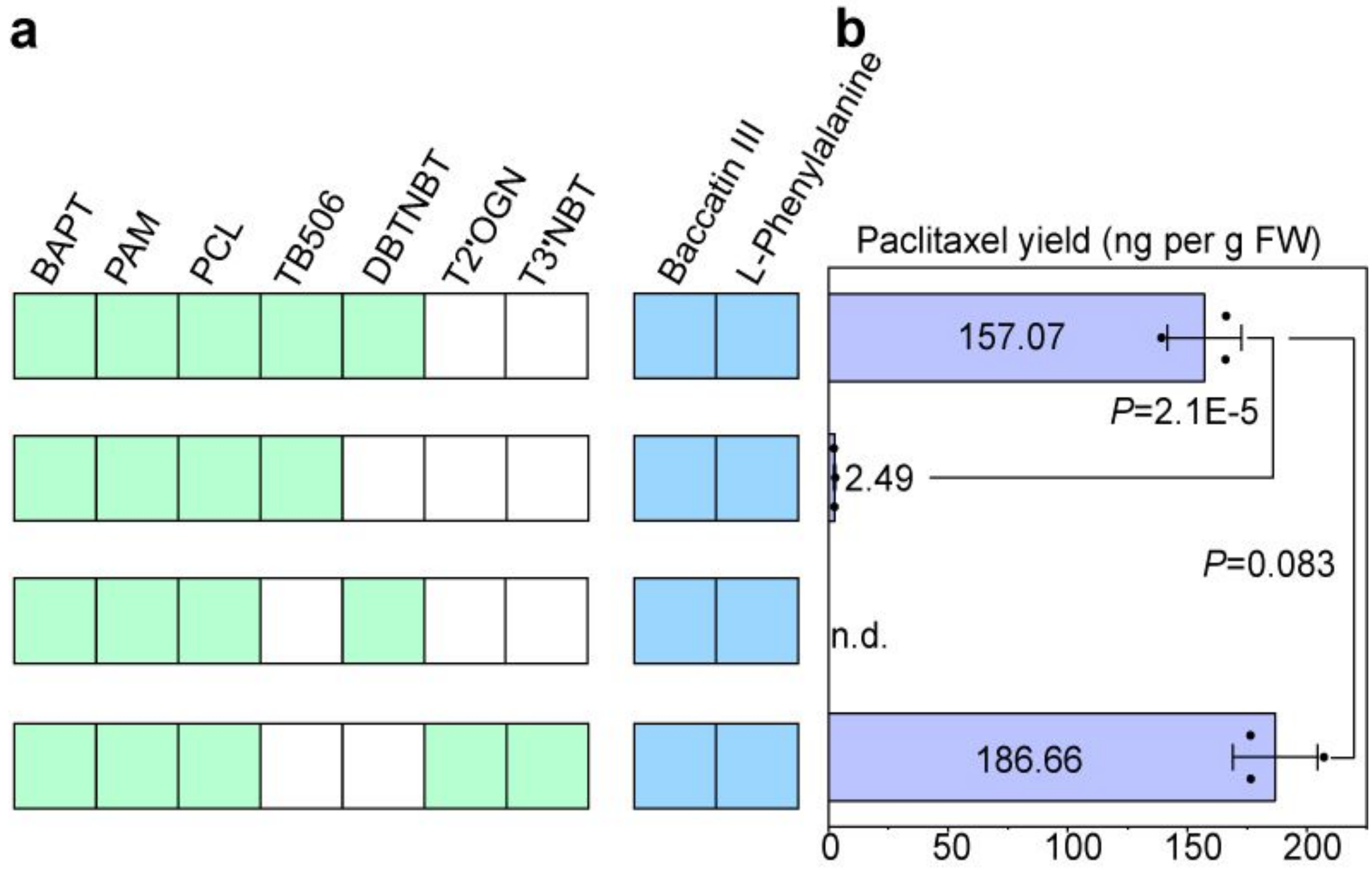

Supplementary Fig. 11| Functional validation of TB506 and DBTNBT**.**

(a) Schematic of enzyme combinations transiently expressed in *P. patens* protoplasts. Filled green squares denote overexpressed enzymes; filled blue squares indicate supplementation with the corresponding exogenous substrate (0.1 mM baccatin III and 1 mg/ml L-Phenylalanine). Blank squares represent the absence of the component.

(b) Quantification of paclitaxel by the transient expression of corresponding enzyme combinations in (a). n.d., not detected. The one-way ANOVA *p*-values were used for significance difference analysis (*p*-values of 0.05 or less indicated statistical significance). Data are mean ± SD (n = 3). All data in this study are derived from biological replicates, with each experimental unit defined as an independent transformation group. Source data are provided as a Source Data file.
