# Supplementary material for: Engineering the paclitaxel biosynthetic pathway in Physcomitrium patens for de novo and stable biosynthesis
Source: Nat Commun. 2026 Oct 21;17:10648. doi: 10.1038/s41467-026-76625-x (PMC13687077; doi:10.1038/s41467-026-76625-x)
Supplement: Supplementary file 2 [file 41467_2026_76625_MOESM2_ESM.pdf]

nature portfolio

# Peer Review File

## Engineering the Paclitaxel Biosynthetic Pathway in Physcomitrium patens for De Novo and Stable Biosynthesis

Corresponding Author: Professor Zhangli Hu

**This file contains all reviewer reports in order by version, followed by all author rebuttals in order by version.**

**Parts of this Peer Review File have been redacted as indicated to remove third-party material.**

Version 0:

Reviewer comments:

Reviewer #1

(Remarks to the Author)
The manuscript by annonymous follows on from a spate of recent papers on paclitaxel biosynthesis. The authors use the geneset described by Zhang et al.´s 2023 paper initially to repeat the work this paper acheived in moss (the intial study was in yeast), but additionally to go considerably further and generate stable lines by splitting the pathway in three and combining the resulting transgenics. They additionally optimized the pathway by modifying the organellar targeting of two of the enzymes.

The work is well designed and well executed I only have relatively minor suggestions for improvement.

Firstly, I think the fact that three different pathways have been postulated in Molecular Plant, Science and Nature should be explicitly discussed in the Introduction and the reason for chosing Zhangs geneset should be provided (possibly the Nature paper was published too late!).

Secondly, I would like the authors to expand the section where they discuss the production capacity of their system. Including the fact that a normal paclitaxel treatment cycle requires 3g of the metabolite. To me this suggests that while the authors work is a considerable breakthrough further refinements will be needed in the future.

Finally, and pedantically, centifugal forces should be provided in <g> not rpm - this should be corrected.

Reviewer #2

(Remarks to the Author)
This manuscript reports the first successful biosynthesis of paclitaxel in the bryophyte Physcomitrella patens. The authors employed a dual-fluorescence reporter system to quantify promoter strength and a self-cleaving 2A peptide strategy to enable multi-gene co-expression, and demonstrate that a complete set of previously characterized paclitaxel biosynthetic genes can be functionally expressed in this heterologous host. This is a significant proof-of-concept which evidences that a non-Taxus plant chassis can carry out the entire series of enzymatic steps for Taxol synthesis. I beleive this is totally worth publication if the comments are addressed

Specific comments:
• The novelty claim is strong, but the manuscript should more explicitly of P. patens relative advantages to other platforms would strengthen the positioning.
Reported paclitaxel yields are low (0.92 µg/g DW; 2.41 µg/L liquid culture). The authors acknowledge this, but additional context is needed. How do these values compare quantitatively to N. benthamiana transient systems, and what needs to be improved to increase these yields.
The 2A peptide strategy is mentioned but not benchmarked. Did the authors verify complete or near-complete cleavage in P. patens?
The choice to remove chloroplast transit peptides from GGPPS and TXS is well justified. Still, the manuscript could benefit from quantitative data on metabolite compartmentalization or enzyme localization (e.g. confocal imaging of tagged proteins).
• Are there indications that flux through the MEP vs MVA pathway constrains precursor supply? Could precursor engineering be a near-term optimisation target?

• The GC-MS and LC-MS chromatograms shown in Figures 2–4 are convincing, but readers would benefit from full spectra (main text or supplementary) and authentic standard overlays. Some extended data figures are referenced but not sufficiently described in the main text.
• Absolute quantification methods should be clarified. Were calibration curves with authentic standards constructed for each metabolite, or was relative quantification used?

Minor comments
• Figures: Some labels are very small (especially in Extended Data). Please enlarge for readability.
• Terminology: Throughout, “paclitaxe” appears instead of “paclitaxel” in some figure legends and text. Please correct.
• Methods: Clarify which codon optimisation algorithm was used. Specify the source/sequence IDs of the paclitaxel pathway genes.
• For clarity and readability, I recommend that the authors label each GC-MS chromatogram peak with the corresponding identified compound. Unidentified peaks can be marked as “unknown” or “putative intermediates”, etc.

Reviewer #3

(Remarks to the Author)
This manuscript reports the reconstruction of the paclitaxel biosynthetic pathway in P. patens (moss), claiming stable de novo production of paclitaxel. While the engineering strategy and use of moss as a chassis are conceptually interesting, the work is fundamentally undermined by its reliance on an outdated pathway structure.

The authors base their design on the enzyme set proposed by Fernie et al. (Zhang, Y. et al. Synthetic biology identifies the minimal gene set required for paclitaxel biosynthesis in a plant chassis. Mol. Plant. 16, 1951-1961), which has since been revised substantially. More recent studies (Jiang, B. et al. Characterization and heterologous reconstitution of Taxus biosynthetic enzymes leading to baccatin III. Science. 383, 622-629 (2024); Liang, F. et al. Elucidation of the final steps in Taxol biosynthesis and its biotechnological production. Nat. Synth. (2025); McClune, C. J. et al. Discovery of FoTO1 and Taxol genes enables biosynthesis of baccatin III. Nature. 643, 582-592 (2025); Yang, C., Wang, Y., Su, Z. et al. Biosynthesis of the highly oxygenated tetracyclic core skeleton of Taxol. Nat Commun 15, 2339 (2024)) have corrected the order of oxidations on the taxane core, identified a validated taxoid 9α-hydroxylase that supports flux to baccatin III, and demonstrated that oxetane formation is catalyzed by CYP725A55 and not by the enzymes used in this manuscript. In these later manuscripts, several of the enzymes implemented here have been shown to be non-functional in the proposed reactions when tested in pathway reconstitutions in tobacco and yeast. The authors neither acknowledge these critical updates nor provide experimental evidence to justify continued use of the Fernie enzyme set.

In addition, the manuscript omits key components now known to be essential for pathway functionality. Notably, it fails to include FoTO1, an NTF2-like factor shown to be required for efficient flux through early steps of the pathway, while it also overlooks the complex acetylation-mediated protection-deprotection mechanism described by McClune et al., which plays a central role in enabling progression through mid-path intermediates.

As demonstrated by Liang et al. and McClune et al., the pathway steps proposed by Fernie were misassigned due to promiscuous background enzyme activities in tobacco cells that supported some conversions at very low levels. In the present manuscript, the authors do not validate each step independently, nor do they provide side-by-side comparisons of alternative pathway architectures. By reconstructing multiple steps simultaneously, this work is confounded by the same issue, background activity from moss enzymes.

Publishing this outdated version of the pathway would propagate incorrect assumptions about enzyme functionality and pathway architecture, mislead future research efforts, and risk wasting resources in other laboratories. Establishing a moss platform for paclitaxel production has both research and commercial value, but this requires reconstructing the pathway in a stepwise manner, critically evaluating each step against the most recent findings, and supporting claims with robust evidence for intermediate identity (including NMR characterization where necessary). Achieving this would essentially require repeating most of the reported work. For these reasons, the flaws cannot be remedied through revision, and I can only recommend rejection.

Version 1:

Reviewer comments:

Reviewer #1

(Remarks to the Author)
The authors have revised their manuscript in a manner that fully addresses all my previous concerns. I have no further comments.

Reviewer #2

(Remarks to the Author)
I am happy with the way the authors have reviewed my comments, and I believe the paper is suitable for publication in the

current format

Reviewer #3

(Remarks to the Author)
I appreciate the additional experiments carried out by the authors. However, these experiments do not address my fundamental concerns regarding the validity of the results and the central claims of the manuscript. Careful examination of the data reinforces the conclusion that it is highly unlikely that paclitaxel is produced by the reconstructed pathway.

The problems in this study arise already at the very first step of the pathway and propagate throughout the entire reconstruction.

1. The pathway reconstruction fails at the initial step, namely the production of taxadiene (endotaxadiene) in moss. Upon expression of taxadiene synthase (TXS), the authors report the appearance of two peaks that are clearly distinct from the two peaks observed when TXS is expressed in E. coli, which serves as their control. The authors attribute this discrepancy to the formation of a taxadiene isomer (exotaxadiene), which they further suggest may be advantageous for pathway reconstruction.

This interpretation is not supported by the data. Expression of TXS in E. coli yields the two expected products, endotaxadiene and exotaxadiene, as confirmed by the MS spectra shown in Supplementary Figs. 1 and 7. These spectra match well with previously published MS data for these compounds (e.g., Zhao et al., J. Am. Chem. Soc. 2024, 146, 801–810; https://doi.org/10.1021/jacs.3c10864), and the relative retention times reported here are consistent with the known elution order of endotaxadiene and exotaxadiene. In contrast, the two peaks observed in moss do not correspond to either endotaxadiene or exotaxadiene based on both retention time and MS characteristics.

Therefore, the two compounds detected in moss are neither endotaxadiene nor exotaxadiene. They are most likely endogenous moss diterpenoids or aberrant products that do not possess the taxadiene skeleton. A plausible explanation for their appearance is the inappropriate choice of controls. To enable taxadiene biosynthesis, the authors co-express HMGR and a GGPPS to boost GGPP supply. However, their control consists of unmodified moss cells lacking these enzymes. Expression of HMGR and/or GGPPS in a plant system is expected to increase GGPP levels and consequently stimulate the production of endogenous diterpenoids. The observed peaks are therefore likely moss-derived diterpenoids produced as a result of enhanced GGPP availability, or potentially geranylgeraniol or geranyllinalool, which are known hydrolysis products of GGPP and show MS features similar to those presented.

Appropriate controls, such as expression of HMGR alone, GGPPS alone, or their combination in the absence of TXS, are entirely missing and would have been essential to identify the origin of these compounds. An alternative possibility is that endotaxadiene or exotaxadiene, if produced at low levels, are rapidly modified by endogenous moss enzymes. In either case, the data do not support the conclusion that bona fide paclitaxel pathway intermediates are produced, casting serious doubt on the validity of the entire pathway reconstruction.

2. The problematic interpretation of the data continues at the next step, where the authors introduce the taxadiene-5α-hydroxylase (CYP725A4). The authors report the formation of taxadiene-5α-ol and provide an MS spectrum in support of this claim. However, comparison of this spectrum with published MS data for taxadiene-5α-ol demonstrates that this assignment is incorrect. The authors can refer, for example, to the supplementary data in McClune et al. (Nature 643, 582–592 (2025), https://doi.org/10.1038/s41586-025-09090-z), where the MS spectrum of taxadiene-5α-ol is reported and differs substantially from the spectrum shown here.

Thus, the compound detected is not taxadiene-5α-ol and cannot be considered a correct pathway intermediate. It is far more likely that this compound results from the activity of the hydroxylase on an endogenous moss diterpene or on the unrelated compounds observed in the previous step. Again, critical controls are missing. In particular, expression of HMGR and GGPPS together with the hydroxylase, but in the absence of TXS, would have revealed whether the detected compound originates from endogenous substrates rather than from taxadiene.
Taken together with the failure to demonstrate correct taxadiene production, this step provides further evidence that the pathway reconstruction is fundamentally flawed.

3. Following the first two problematic steps, it is not possible to assess or validate the function of any of the downstream enzymes. The authors introduce all remaining pathway genes simultaneously, without providing data for individual steps or appropriate enzyme-omission controls. No chromatograms are shown for intermediates corresponding to individual enzymatic reactions, and there is no validation of their identities.

Moreover, there is no evidence that the heterologously expressed enzymes are even produced in moss (e.g., by Western blot). Given the clear issues already identified at the earliest steps of the pathway (no production of taxadiene or taxadien-5a-ol), the absence of stepwise validation and expression controls makes it impossible to assign any observed metabolites to the intended enzymatic activities.

4. Eventually, the authors claim the production of baccatin III and paclitaxel. However, the chromatographic and MS data

provided in support of these claims are extremely limited and insufficient for compound identification. The chromatograms are narrowly cropped to show only a small retention time window around a single peak, and the MS spectra are similarly truncated to display only one major fragment ion, while omitting the diagnostically critical lower m/z region.
With such selectively presented data, it is not possible to confirm the identity of these compounds. To support these claims, the authors would need to present complete chromatograms, including all relevant negative controls in which individual pathway genes are omitted, and extracted ion chromatograms for all diagnostic fragment ions of both standards and samples. Full, untrimmed MS spectra should be provided for all proposed intermediates and final products.
In the absence of these data, the reported detection of baccatin III and paclitaxel cannot be validated.

5. The substrate feeding experiments are critically undermined by the fact that baccatin III preparations are not chemically pure and are well known to contain additional taxanes, including paclitaxel itself. Consequently, the detected paclitaxel signal could arise from trace paclitaxel contamination in the baccatin III preparation, or from the activity of one or more introduced enzymes on other taxanes or pathway intermediates present in the fed substrate, rather than from de novo paclitaxel biosynthesis.

Importantly, in the revised manuscript, paclitaxel is detected even when baccatin III is supplied to cells expressing an incomplete set of pathway enzymes (Supplementary Fig. 10, second and third samples from the top). Under these conditions, paclitaxel biosynthesis should not be possible, as essential enzymatic steps are absent. The detection of paclitaxel in these samples therefore strongly suggests that the observed signal originates either from paclitaxel already present in the supplied substrate, from enzymatic modification of contaminating intermediates, or that the compound assigned as paclitaxel has been misidentified.

6. The study also fails to address key discrepancies between the results and the literature. For example, for their reconstruction, the authors utilize the 9a-hydroxylase CYP725A22-1/CYP725A37. It is well established that this CYP requires the presence of an acetyl group at the C-13 of the taxadiene skeleton to functon(McClune et al., Nature 643, 582–592 (2025). https://doi.org/10.1038/s41586-025-09090-z; Yang et al., Nat Commun 15, 2339 (2024). https://doi.org/10.1038/). There is no C-13 acetyltransferase in the utilized enzyme set. How are then baccatin III or paclitaxel produced? Even if a moss enzyme could non-specifically catalyze this step, the added C-13 acetyl group would have to be subsequently removed to produce baccatin II and to allow the attachment of the C-13 side chain to eventually synthesize paclitaxel. Neither acetylation nor deacetylation at C-13 is accounted for or discussed. This omission represents a major gap in the pathway the authors are aiming to reconstruct.

7. In addition to the above, my earlier concerns about essential pathway components, like FoTO1, TOT1, T7dA, and T9dA, missing from the reconstruction, were not adequately addressed. Mentions to the relevant publications were added in the text, but their functional significance for the pathway was neither discussed nor experimentally validated.

Taken together, the key concerns raised above propagate from the earliest steps to the full pathway and are fundamental for the study. In my view, the data far from support the manuscript's central conclusion, and resolving these problems would require a fundamentally different experimental and analytical approach rather than revision of the current study.

Version 2:

Reviewer comments:

Reviewer #3

(Remarks to the Author)
The revised manuscript and rebuttal do not resolve my central concerns. On the contrary, several of the experiments provided in the revision reinforce the concerns raised in the previous rounds of review. The manuscript claims "de novo reconstruction of the paclitaxel biosynthetic pathway in moss." However, the revised data instead suggest that paclitaxel formation in these experiments depends on a fortuitous combination of introduced taxane biosynthetic genes and unidentified endogenous P. patens activities. This is a fundamentally different conclusion: paclitaxel production in this system is a fortuitous outcome, not the result of legitimate pathway reconstruction.

The most important evidence comes from two key experiments:

First, in the late-stage enzyme-omission experiment, the authors report that paclitaxel can still be detected when DBTNBT is omitted, and they interpret this result as evidence that P. patens may contain an endogenous enzyme with DBTNBT-like activity. This observation is highly significant because it directly supports the concern that the reported paclitaxel signal arises through fortuitous background metabolism in moss. If a terminal activity required for paclitaxel formation is supplied by an unidentified moss enzyme, then the pathway has not been reconstructed from the introduced gene set.

Second, the same concern is reinforced by the experiment in which the authors report a marked increase in paclitaxel production after introducing FoTO1 and replacing the originally used terminal enzymes with T2′OGD and T3′NBT. These introduce key enzymes from more recent pathway models that were absent from the original reconstruction. The large effect

of these additions strongly supports the point raised repeatedly in the previous rounds of review: the original gene set was incomplete, and the reported paclitaxel production depends on functions not included in the initial reconstruction.

These findings also reinforce another central concern raised, which has not been addressed in the revision: the pathway has not been validated step by step. The authors argue that endpoint detection of baccatin III or paclitaxel is sufficient to demonstrate pathway function. However, their own data show that this reasoning is flawed: paclitaxel is apparently detected even when an expected enzyme is omitted. Thus, endpoint detection cannot be used to infer that the introduced pathway functions as proposed. Each step must be validated with enzyme-addition and enzyme-omission controls, appropriate negative controls, and rigorous intermediate identification. Without this, the contribution of the introduced genes cannot be distinguished from endogenous moss metabolism.

This point is particularly important because the field of paclitaxel biosynthesis has advanced substantially beyond the pathway model on which this manuscript is based. The authors use the Zhang et al. pathway model. However, more recent pathway reconstructions, including those by McClune et al. and Liang et al., include several additional enzymatic and auxiliary functions that are absent from present manuscript. In addition to FoTO1 and T3NBT which were proven to be critical in the revised manuscript, omissions include the pathway-specific T9αH-750C rather than the CYP725A22-1/CYP725A37 route used in the Zhang model; T7AT, which stabilizes the C7β-hydroxylated intermediate and facilitates productive 2α-benzoylation; TOT, which is required for oxetane formation; T1βH, which acts after oxetane formation; the deacetylases T9dA and T7dA; and T9ox, which completes the C9 oxidation state needed for baccatin III. Recent work also suggests that additional cryptic deacetylation chemistry, including C13α-O-deacetylation, may be part of the pathway logic (https://www.biorxiv.org/content/10.64898/2026.04.28.721278v1). Thus, the differences are not limited to one optional enzyme or one yield-enhancing factor; they affect the architecture of the pathway from the early oxidation step through baccatin III formation and the final conversion to paclitaxel.

FoTO1 is a clear example. The authors describe FoTO1 as non-essential because it is not a canonical catalytic enzyme. However, recent work shows that FoTO1 is not merely a minor yield-improvement factor as the authors claim. It changes the outcome of the first oxidation step, suppresses non-productive OCT/iso-OCT formation, and promotes formation of the productive taxadien-5α-ol-derived pathway (https://www.biorxiv.org/content/10.64898/2026.03.21.713420v1; https://www.biorxiv.org/content/10.64898/2026.03.30.715439v1). In the early-pathway data presented in this manuscript, taxadien-5α-ol appears to be only a small fraction of the oxygenated products, while OCT/iso-OCT dominate. Therefore, including FoTO1 is not optional. It is essential for proper pathway reconstruction.

The same applies to oxetane formation. The authors dismiss the absence of TOT by referring to earlier pathway models in which baccatin III or related intermediates were reported without this enzyme. However, the formation of the oxetane-containing core is a defining step in paclitaxel biosynthesis. Multiple recent studies have identified specific oxetane-forming enzymes and have shown that this chemistry is not a trivial spontaneous process that can simply be assumed to occur in any host. If the authors propose that moss produces baccatin III and paclitaxel without a validated oxetanase, they must show the corresponding oxetane-containing intermediates and identify the activity responsible. Endpoint LC-MS detection of baccatin III or paclitaxel does not resolve this mechanistic gap.

The authors' response also fails to resolve the additional mechanistic concerns about the utilized enzyme set around C13 acetylation. This is a critical flaw. The original point was not simply whether CYP725A22-1 and CYP725A37 are identical enzymes, or whether the Zhang et al. pathway model differs from the McClune et al. model. The concern is that the pathway proposed by the authors does not account for the substrate requirements and functional-group changes required to reach baccatin III and paclitaxel.
The authors respond that the 9α-hydroxylase used in their study is CYP725A22-1 from Zhang et al., and that this enzyme is distinct from the CYP725A37 discussed in other studies. I believe that the authors should confirm and compare the sequences of these enzymes more carefully. More importantly, they argue that Zhang et al. proposed a different reaction sequence and that their successful detection of baccatin III and paclitaxel demonstrates that this gene set is functional in moss. This argument is not sufficient. The authors still do not show that CYP725A22-1 acts on the actual intermediate generated in their moss system, nor do they identify the intermediates that would demonstrate this proposed reaction sequence.

This is important because the most recent pathway reconstructions raise a specific mechanistic problem for the Zhang-type C9α-hydroxylase route. McClune et al. report that replacing T9αH-750C with T9αH-725A gives negligible baccatin III production, statistically indistinguishable from a ΔTDS negative control, and state that this is expected because T9αH-725A appears to require 13α-O-acetylation, which is absent from their baccatin III pathway and from Taxol. This directly relates to the concern raised in the review: if the authors use a C9α-hydroxylase route that requires a C13-acetylated substrate, then the pathway must also include the enzyme that installs the C13 acetyl group and the enzyme that later removes it to regenerate the C13 hydroxyl required for side-chain attachment. Neither step is accounted for in the manuscript.

In addition to not clarifying the paclitaxel pathway, this distinction also affects the central biotechnology claim of this manuscript. If paclitaxel formation depends on unidentified moss activities, then the system cannot be rationally optimized, transferred, or engineered in the way implied by the manuscript. Without identifying the endogenous enzymes responsible for these missing steps, the authors cannot determine which reactions are limiting, which products are formed through the intended route, or how the pathway should be improved. The reported fortuitous production therefore does not establish a validated and expandable platform for paclitaxel biosynthesis.

Reviewer #4

(Remarks to the Author)
In my opinion, the manuscript reports an important and technically impressive advance. The authors have transferred a large paclitaxel biosynthetic gene set into Physcomitrium patens and demonstrate production of taxadiene-5α-ol, baccatin III, and paclitaxel through a modular engineering strategy. The establishment of a genetically stable moss line producing paclitaxel is a significant achievement and, in my view, is suitable for publication in Nature Communications, provided that the biochemical claims are phrased carefully.
First, DBTNBT has been functionally characterized in previous studies as a taxoid side-chain N-benzoyltransferase, including the original work by Walker, Long and Croteau and more recent work showing that overexpression of BAPT and DBTNBT can increase paclitaxel accumulation in Taxus baccata cultures. Therefore, the inclusion of DBTNBT in the pathway is biochemically justified. Nevertheless, because paclitaxel production is substantially reduced upon DBTNBT omission, the data still support a functional contribution of DBTNBT to the reconstructed pathway.
Second, the addition of FoTO1 and the replacement of TB506/DBTNBT with T2′OGD/T3′NBT should not be interpreted as disproving the original reconstruction. In metabolic engineering, it is common that an initial pathway is functional but later improved by newly identified enzymes or accessory factors. FoTO1 is now known to improve early taxane oxidation, and T2′OGD/T3′NBT provide an alternative and apparently more efficient terminal route. Their use strengthens the engineering result rather than negating the original pathway. However, the manuscript should clearly distinguish between the “initial reconstructed pathway” and the “optimized pathway.” The strongest claim should be that moss can serve as a stable plant chassis for paclitaxel biosynthesis, not that the initially introduced gene set represents the uniquely complete or definitive Taxus pathway.
Third, I partly agree that the pathway has not been validated step by step in the strict biochemical sense in this story while it has been validated by other research in Zhang et al 2023 Molecular plant. The authors validate major modules: taxadiene/taxadiene-5α-ol, baccatin III, and paclitaxel. This is acceptable for a synthetic biology reconstruction paper, particularly because many individual enzyme functions have already been reported in prior publications. However, the authors should avoid claiming full mechanistic resolution of every enzymatic step in moss unless all intermediates are directly detected.
Fourth, the concern about unresolved C13-side-chain/late-stage biochemistry is fair. The current data do not fully establish the exact late-stage reaction sequence or all relevant intermediates in the moss chassis. In particular, if the pathway proceeds differently from the proposed linear sequence, or if moss endogenous enzymes participate, this should be acknowledged explicitly. However, this mechanistic uncertainty is not unusual in paclitaxel biosynthesis, which is increasingly understood as a branched and flexible biosynthetic network rather than a single invariant linear route. Therefore, I do not think this issue should preclude publication, but it should lead to more cautious wording.

Overall, I recommend publication after revision. The authors should be asked to temper the title/abstract/discussion claims so that the work is presented as a stable moss-based reconstruction and optimization of previous paclitaxel biosynthesis, rather than as definitive proof that every catalytic activity required for paclitaxel formation is supplied solely by the originally introduced gene set. They should also explicitly discuss the residual paclitaxel signal in the DBTNBT-omission experiment as evidence for possible endogenous moss acyltransferase activity or pathway flexibility. With these clarifications, the main conclusion—that P. patens can be engineered as a stable plant chassis for de novo paclitaxel production—is sufficiently supported.

Version 3:

Reviewer comments:

Reviewer #4

(Remarks to the Author)
I think the authors have addressed all of my concerns. Given that the pathway reported by Zhang et al. in 2023 was first submitted to Nature, although it was not successful, I am pleased to see that our results can now be independently reproduced by another group. I believe this represents an important contribution to the field.

**Open Access** This Peer Review File is licensed under a Creative Commons Attribution 4.0 International License, which permits use, sharing, adaptation, distribution and reproduction in any medium or format, as long as you give appropriate credit to the original author(s) and the source, provide a link to the Creative Commons license, and indicate if changes were made.
In cases where reviewers are anonymous, credit should be given to 'Anonymous Referee' and the source.
The images or other third party material in this Peer Review File are included in the article's Creative Commons license, unless indicated otherwise in a credit line to the material. If material is not included in the article's Creative Commons license and your intended use is not permitted by statutory regulation or exceeds the permitted use, you will need to obtain permission directly from the copyright holder.
To view a copy of this license, visit https://creativecommons.org/licenses/by/4.0/

Dear Editor and reviewers,

We sincerely appreciate the time and effort dedicated by the editor and reviewers, and we are deeply grateful for the valuable comments they have provided. The detailed responses to the reviewers' comments were listed below. And the manuscript was thoroughly revised according the comments.

Thank you and best wishes!

Yours Sincerely

**REVIEWER COMMENTS**

Reviewer #1 (Remarks to the Author):

The manuscript by annonymous follows on from a spate of recent papers on paclitaxel biosynthesis. The authors use the geneset described by Zhang et al.´s 2023 paper initially to repeat the work this paper acheived in moss (the intial study was in yeast), but additionally to go considerably further and generate stable lines by splitting the pathway in three and combining the resulting transgenics. They additionally optimized the pathway by modifying the organellar targeting of two of the enzymes. The work is well designed and well executed I only have relatively minor suggestions for improvement.

We are honored by the reviewer's positive evaluation of our work and deeply grateful for the constructive comments and time reviewer dedicated to reviewing our manuscript. We have revised the manuscript based on the suggestions provided.

1) Firstly, I think the fact that three different pathways have been postulated in Molecular Plant, Science and Nature should be explicitly discussed in the Introduction and the reason for chosing Zhangs geneset should be provided (possibly the Nature

paper was published too late!).

Response:

We sincerely thank the reviewer for their valuable comment. We have thoroughly discussed the three distinct biosynthetic pathways of baccatin III or paclitaxel in the Introduction section, as detailed in lines 51-82 of the resubmitted manuscript.

The gene set reported by Zhang et al. was selected in this study for the following reasons. As the Fernie's team (Zhang et al., *Mol. Plant*. 2023 Dec 4;16(12):1951-1961) was the first to report the complete paclitaxel biosynthetic pathway, and its baccatin III yield (approximately 100-fold higher than that of the Yan team's approach (Jiang et al., *Science*. 2024 Feb 9;383(6683):622-629) demonstrated superior application potential, we adopted this pathway and successfully performed its stepwise validation using a transient expression system in *P. patens*. As reviewer noted, the gene combination from the Nature article (McClune et al., *Nature*. 2025 Jul 11;643(8071):582-592) was not included in our initial experimental design, primarily because the article was published (2025.06.11) after our study had been largely completed. The rationale for selecting this gene set has been added to the Introduction section of the revised manuscript (see lines 120-132 of the resubmitted version).

2) Secondly, I would like the authors to expand the section where they discuss the production capacity of their system. Including the fact that a normal paclitaxel treatment cycle requires 3g of the metabolite. To me this suggests that while the authors work is a considerable breakthrough further refinements will be needed in the future.

Response:

We sincerely thank the reviewer for their valuable comment. We have elaborated in detail from four perspectives in the Discussion section on the current production capacity of the system and potential strategies for further optimization in the future, namely: optimizing the key rate-limiting steps; replacing the 2A peptide with different

strong promoters; introducing high-activity enzyme genes; and increasing precursor substances (see lines 291-323 of the resubmitted version).

3) Finally, and pedantically, centifugal forces should be provided in <g> not rpm - this should be corrected.

Response:

We sincerely thank the reviewer for their valuable comment. We have changed the speed unit to g in the line 467 of resubmitted manuscript.

Reviewer #2 (Remarks to the Author):

This manuscript reports the first successful biosynthesis of paclitaxel in the bryophyte *Physcomitrella patens*. The authors employed a dual-fluorescence reporter system to quantify promoter strength and a self-cleaving 2A peptide strategy to enable multi-gene co-expression, and demonstrate that a complete set of previously characterized paclitaxel biosynthetic genes can be functionally expressed in this heterologous host. This is a significant proof-of-concept which evidences that a non-Taxus plant chassis can carry out the entire series of enzymatic steps for Taxol synthesis. I beleive this is totally worth publication if the comments are addressed.

We sincerely thank the reviewer for their time and for their thoughtful feedback, which we found not only constructive but also appreciative of the study's novelty. We have addressed each question individually and made corresponding revisions to the resubmitted manuscript.

Specific comments:

1) The novelty claim is strong, but the manuscript should more explicitly of *P. patens* relative advantages to other platforms would strengthen the positioning.

Response:

We sincerely thank the reviewer for their valuable comment.

In this study, the chassis cell selected—*Physcomitrium patens*, often referred to as the “green yeast”—exhibits typical advantages of eukaryotic expression systems, such as sufficient supply of key precursor substances like GGPP and ample NADPH, the correct folding and subcellular compartmentalization of heterologous eukaryotic proteins. Furthermore, this system is operationally convenient: it allows easy preparation of protoplasts and is suitable for rapid gene function validation via transient expression. Its nuclear genome exhibits high homologous recombination efficiency, facilitating precise integration of large foreign DNA fragments into

specific loci. In addition, the plant demonstrates strong regenerative capacity and can efficiently regenerate into intact plants from protoplasts, making it an ideal plant platform for synthetic biology and metabolic engineering research. We have supplemented the advantages of *P. patens* compared to other platforms in the Introduction of the newly submitted manuscript (see lines 109-119).

2) Reported paclitaxel yields are low (0.92 μg/g DW; 2.41 μg/L liquid culture). The authors acknowledge this, but additional context is needed. How do these values compare quantitatively to *N. benthamiana* transient systems, and what needs to be improved to increase these yields.

Response:

We sincerely thank the reviewer for their valuable comment.

The amount of paclitaxel biosynthesis in engineered moss strains reached 0.17 μg/g (FW), 0.92 μg/g (DW) in solid culture or 2.41 μg/L in liquid culture. A maximum yield of 64.29 ng/g (FW) using a tobacco transient expression system, though not in a stable transgenic line (Zhang et al., *Mol. Plant*. 2023 Dec 4;16(12):1951-1961). In comparison, the paclitaxel production in our engineered moss system was 2.7 times higher than that of the tobacco transient system under solid culture conditions. We have added a comparison of our yield with that achieved by transient expression in tobacco chassis in the Discussion section of the newly submitted manuscript, as detailed in lines 266-274.

In the “Discussion” of resubmitted manuscript, we optimized the production capacity of the current system from four aspects: optimizing the rate-limiting steps, replacing the 2A peptide with different strong promoters, introducing high-activity enzymes, and increasing the supply of precursor substances (see lines 291-323).

3) The 2A peptide strategy is mentioned but not benchmarked. Did the authors verify complete or near-complete cleavage in *P. patens*?

Response:

We sincerely thank the reviewer for their valuable comment.

Self-cleaving 2A peptides exhibit higher cleavage efficiency. Wang et al. demonstrated in *Arabidopsis* that the cleavage efficiency of P2A, E2A, and T2A varies between approximately 70% and 95%. Among them, P2A exhibits the highest self-cleavage efficiency (Wang et al., *aBIOTECH*. 2020 Nov 20;1(1):6-14). Meanwhile, Pan et al. successfully constructed a 2A-linked multi-gene cassette for sclareol biosynthesis in .*P. patens* and detected the production of sclareol (Pan et al., *Plant Biotechnol. Rep*. 2015 May 6;9:149-159).

In this study, we employed the P2A peptide-based multicistronic system. To validate the effectiveness of the 2A peptide in *P. patens*, we used the P2A peptide to link taxadiene synthase (TXS) with green fluorescent protein (GFP), while also directly fusing GFP to the C-terminus of TXS. The cleavage of the P2A peptide in *P. patens* protoplasts was assessed via confocal microscopy. As shown in the figure below, unlike the chloroplast localization observed for TXS-GFP, in protoplasts transformed with TXS-P2A-GFP, most of the GFP fluorescence was distinctly distributed in the cytoplasm, indicating that the GFP protein had been cleaved from TXS.

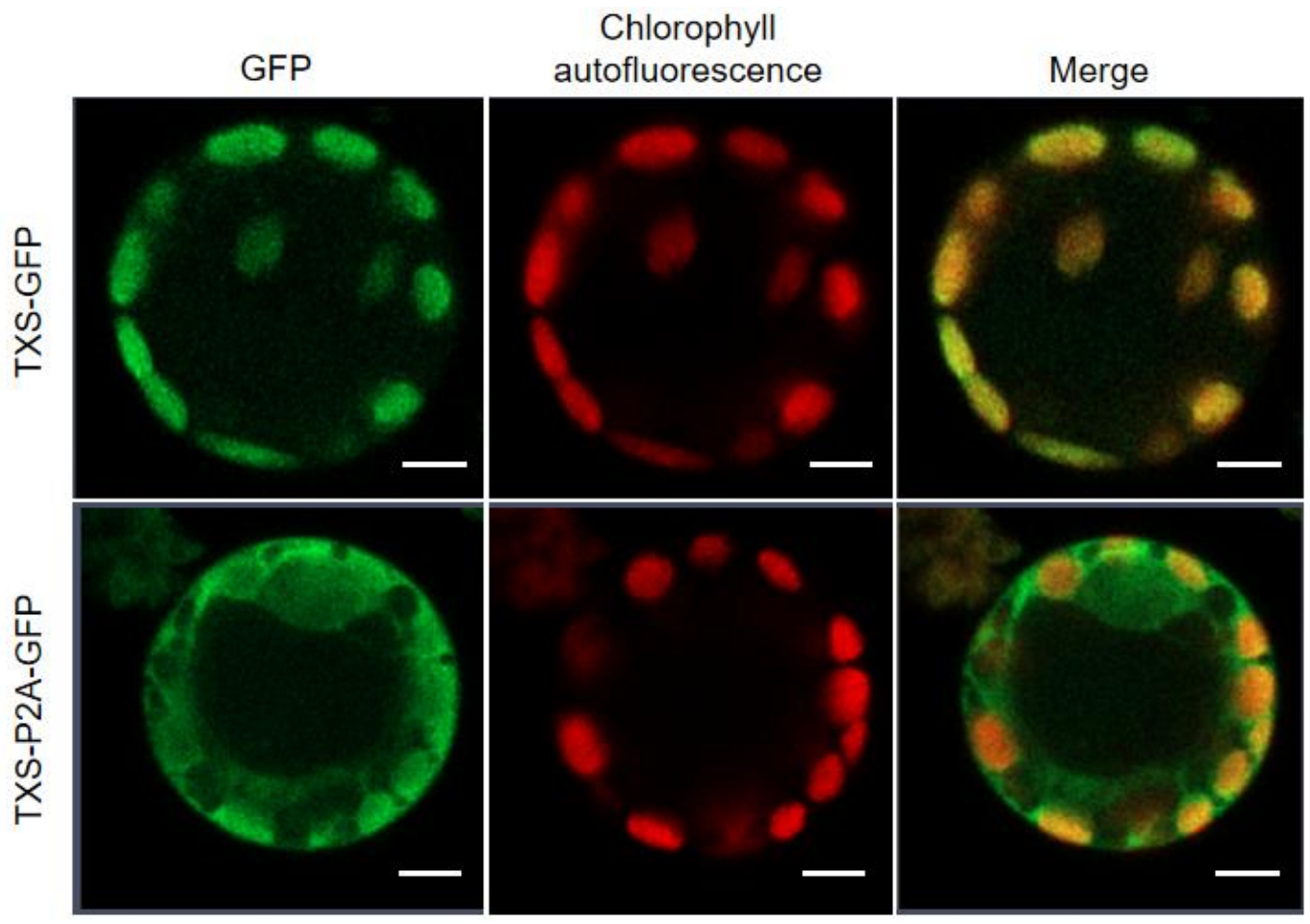

**Supplementary Fig. 5|Subcellular localization of TXS-GFP and TXS-P2A-GFP in *P. patens* protoplasts.**

TXS-GFP and TXS-P2A-GFP were driven by the CaMV 35S promoter and terminated by the NOS terminator. TXS-GFP: GFP was fused to the C-terminus of TXS. TXS-P2A-GFP: the TXS and GFP were linked via P2A. Individual plasmids were introduced into P. patens protoplasts via PEG-mediated transformation. Protoplasts were harvested 1 day after transformation and observed using confocal microscopy. From left to right: GFP channel, chloroplast autofluorescence, and merged images. Scale bars: 5 μm.

We have supplemented the corresponding results description and images in the Results section and the Supplementary Information of the manuscript, respectively (located at lines 220-227 and 815-824).

4) The choice to remove chloroplast transit peptides from GGPPS and TXS is well justified. Still, the manuscript could benefit from quantitative data on metabolite compartmentalization or enzyme localization (e.g. confocal imaging of tagged proteins).

Response:

We sincerely thank the reviewer for their valuable comment.

GFP was fused to the C-terminus of the full-length and signal-peptide-deleted TXS and GGPPS and transiently expressed in *P. patens* protoplasts, and their subcellular localization was visualized by fluorescence microscopy (Supplementary Fig. 2). The results showed that the green fluorescent chimeras derived from TXS/GFP and

GGPPS/GFP completely overlapped with the spontaneous red fluorescence of chloroplasts (Supplementary Fig. 2). In contrast, the green fluorescent derived from signal-peptide-deleted TXS and GGPPS were dispersed in the cytoplasm (Supplementary Fig. 2). This confirmed that we had successfully removed the signal peptides.

We have supplemented the corresponding results description and images in the Results section and the Supplementary Information section of the manuscript, respectively (located at lines 174-187 and 781-789).

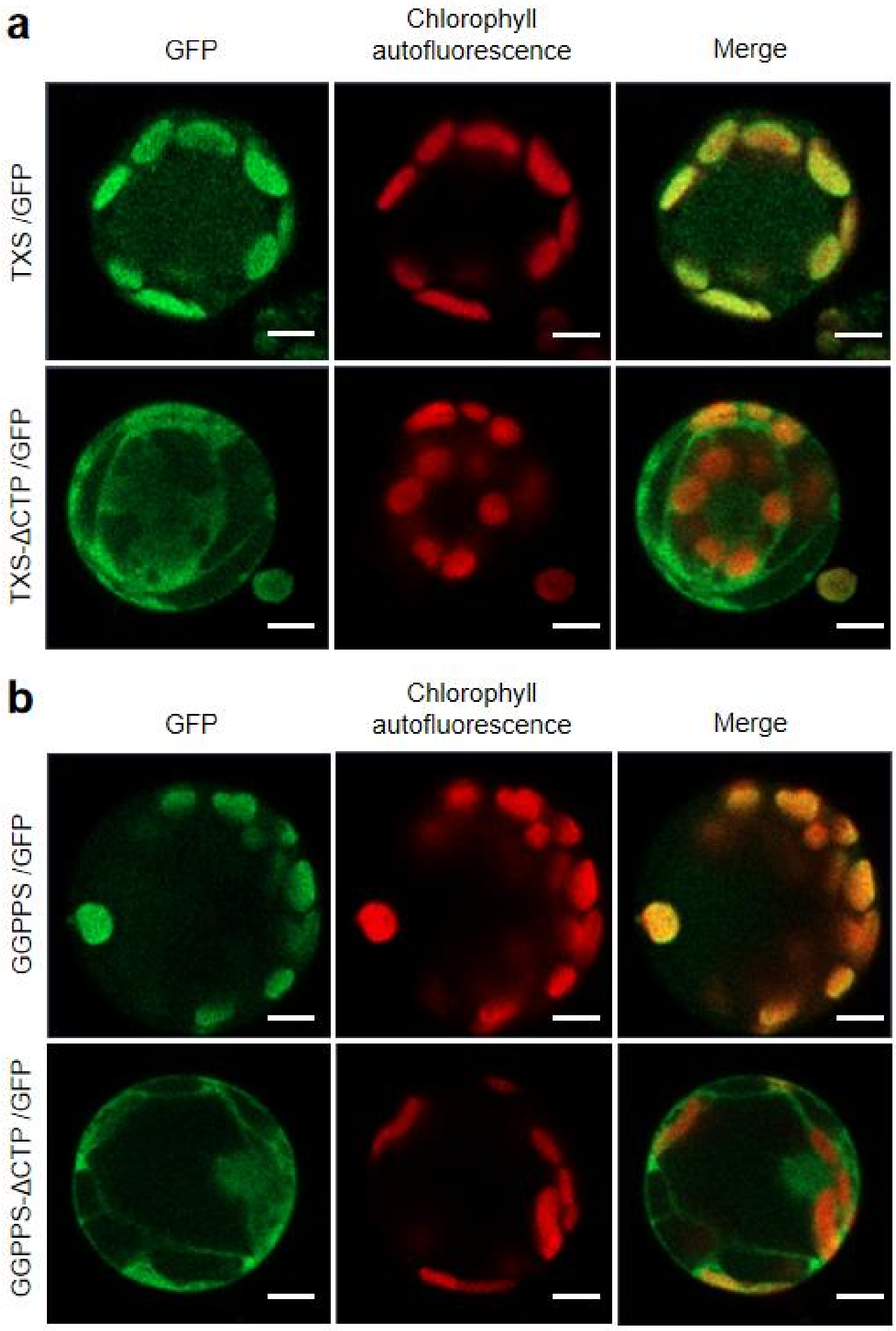


**Supplementary Fig. 2|Subcellular localization and targeting of heterologous proteins.**

Fluorescence labeling of the fusion proteins: a TXS-/GFP, TXS-ΔCTP/GFP; b GGPPS-/GFP, GGPPS-ΔCTP/GFP. GFP was fused to the C-terminus of the target

protein, and individual plasmids were introduced into *P. patens* protoplasts via PEG-mediated transformation. Protoplasts were harvested 1 day after transformation and observed using confocal microscopy. Left to right: GFP channel, Chlorophyll autofluorescence, and merged images. Scale bars: 5 μm.

5) Are there indications that flux through the MEP vs MVA pathway constrains precursor supply? Could precursor engineering be a near-term optimisation target?

Response:

We sincerely thank the reviewer for their valuable comment.

Studies have shown that the flow of MEP vs. MVA pathway limits the supply of precursors (Li et al., *Bioresour. Technol.* 2022 Sep 12:363:127981). One of the optimization strategies mentioned in the Discussion section is to optimize precursor engineering (see lines 319-323).

6) The GC-MS and LC-MS chromatograms shown in Figures 2–4 are convincing, but readers would benefit from full spectra (main text or supplementary) and authentic standard overlays. Some Supplementary figures are referenced but not sufficiently described in the main text.

Response:

We sincerely thank the reviewer for their valuable comment.

We have already provided comprehensive descriptions of the supplementary data in the main text.

7) Absolute quantification methods should be clarified. Were calibration curves with authentic standards constructed for each metabolite, or was relative quantification used?

Response:

We sincerely thank the reviewer for their valuable comment.

We performed quantitative analysis of baccatin III and paclitaxel using a QTRAP 6500+ Low Mass by monitoring both the precursor and fragment ions (representing two characteristic ion pairs). The quantitative method was adapted from the work of Yi et al (Yi et al., *J. Appl. Lab. Med*. 2016 Jul 1;1(1):14-24). The standard solutions were sequentially diluted into six concentration gradients to establish a linear calibration curve based on the peak areas obtained from the instrument. The concentrations of the samples were subsequently determined by correlating their measured peak areas with the established calibration curve. The relevant content has been added to the Methods section (see lines 492-500).

Minor comments

8) Figures: Some labels are very small (especially in Extended Data). Please enlarge for readability.

Response:

We sincerely thank the reviewer for their valuable comment.

We have enlarged all labels in the Figures of the newly submitted manuscript.

9) Terminology: Throughout, “paclitaxe” appears instead of “paclitaxel” in some figure legends and text. Please correct.

Response:

We sincerely thank the reviewer for their valuable comment. We have revised all instances of "paclitaxe" to "paclitaxel" in the newly submitted manuscript.

10) Methods: Clarify which codon optimisation algorithm was used. Specify the source/sequence IDs of the paclitaxel pathway genes.

Response:

We sincerely thank the reviewer for their valuable comment.

The codon optimization was performed using the GeneOptimizer Algorithm (Raab. et al. *Syst. Synth. Biol*. 2010 Sep 1;4(3):215-225.). The algorithm intelligently balances multiple parameters, including codon usage preference, secondary structure minimization, elimination of repetitive sequences and cryptic splice sites, removal of restriction enzyme cleavage sites, GC content control, design of homologous recombination regions, and eradication of detrimental motifs. We have incorporated the codon optimization method into the Methods section of resubmitted manuscript (see lines 386-392).

The gene IDs for the paclitaxel biosynthetic pathway used in the study are provided in Supplementary Table 2 of resubmitted manuscript.

11) For clarity and readability, I recommend that the authors label each GC-MS chromatogram peak with the corresponding identified compound. Unidentified peaks can be marked as "unknown" or "putative intermediates", etc.

Response:

We sincerely thank the reviewer for their valuable comment.

We have labeled all GC-MS chromatographic peaks in the Figures of newly submitted manuscript.

Reviewer #3 (Remarks to the Author):

This manuscript reports the reconstruction of the paclitaxel biosynthetic pathway in *P. patens* (moss), claiming stable de novo production of paclitaxel. While the engineering strategy and use of moss as a chassis are conceptually interesting, the work is fundamentally undermined by its reliance on an outdated pathway structure.

We thank the reviewer for their time and comments. While addressing the comments, we have gained a deeper understanding and developed further insights into the biosynthesis of paclitaxel. We have carefully considered all feedback and revised the manuscript accordingly. Below is a point-by-point response to the comments.

1) The authors base their design on the enzyme set proposed by Fernie et al. (Zhang, Y. et al. Synthetic biology identifies the minimal gene set required for paclitaxel biosynthesis in a plant chassis. Mol. Plant. 16, 1951-1961), which has since been revised substantially.

More recent studies (Jiang, B. et al. Characterization and heterologous reconstitution of Taxus biosynthetic enzymes leading to baccatin III. Science. 383, 622-629 (2024);
Liang, F. et al. Elucidation of the final steps in Taxol biosynthesis and its biotechnological production. Nat. Synth. (2025);
McClune, C. J. et al. Discovery of FoTO1 and Taxol genes enables biosynthesis of baccatin III. Nature. 643, 582-592 (2025);
Yang, C., Wang, Y., Su, Z. et al. Biosynthesis of the highly oxygenated tetracyclic core skeleton of Taxol. Nat Commun 15, 2339 (2024)) have corrected the order of oxidations on the taxane core, identified a validated taxoid 9α-hydroxylase that supports flux to baccatin III, and demonstrated that oxetane formation is catalyzed by CYP725A55 and not by the enzymes used in this manuscript. In these later manuscripts, several of the enzymes implemented here have been shown to be non-functional in the proposed reactions when tested in pathway reconstitutions in tobacco and yeast. The authors neither acknowledge these critical updates nor provide

experimental evidence to justify continued use of the Fernie enzyme set.

Response:

We sincerely thank the reviewer for their valuable comment.

We have thoroughly reviewed recent advances in the Introduction section and extensively discussed current pathways in the Discussion section. Moreover, we have provided experimental validation data to support our arguments in the subsequent sections.

To evaluate the function of the epoxidase reported by Zhang et al., we designed a comparative experiment. The complete baccatin III biosynthetic gene set used in this study (control group) and the version of this gene set with the aforementioned epoxidase removed (experimental group) were separately co-expressed in protoplasts. Metabolite detection revealed that the experimental group produced only trace amounts of baccatin III, whereas the yield in the control group was approximately 213-fold higher than that in the experimental group. The detection of trace amounts of baccatin III even in the absence of the introduced epoxidase suggests that *P. patens* may contains endogenous gene(s) with epoxidase function. This result confirms that the epoxidase identified by Zhang et al. plays a key catalytic role in the biosynthetic pathway of baccatin III.

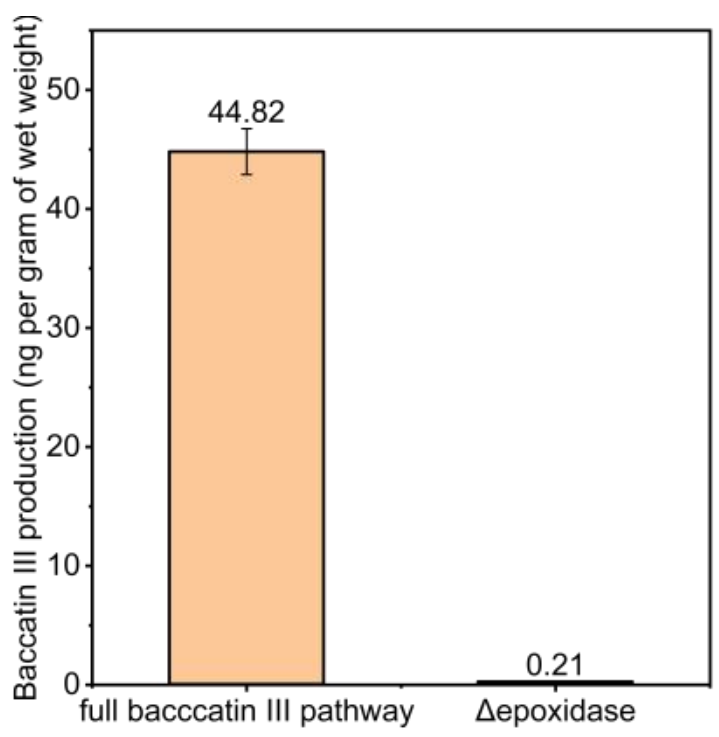


**Supplementary Fig. 9| Epoxidase is essential for baccatin III biosynthesis in the heterologous pathway.**

The "full baccatin III pathway" comprises genes encoding GGPPS-ΔCTP, TXS-ΔCTP, T5αOH, HMGR, CPR, epoxidase, TAT, T10βOH, T13αOH, T2αOH, T9αOH, T7βOH, T1βOH, TBT, and DBAT. The "Δepoxidase" pathway is identical but lacks the

epoxidase gene. Data are presented as the mean ± SD of three biological replicates.

2) In addition, the manuscript omits key components now known to be essential for pathway functionality. Notably, it fails to include FoTO1, an NTF2-like factor shown to be required for efficient flux through early steps of the pathway, while it also overlooks the complex acetylation-mediated protection-deprotection mechanism described by McClune et al., which plays a central role in enabling progression through mid-path intermediates.

Response:

We sincerely thank the reviewer for their valuable comment.

FoTO1 discovered by McClune et al. can reduce the production of OCT and Iso-OCT and increase the yield of taxadiene-5α-ol. However, taxadiene-5α-ol can still be produced based on TXS and T5αOH co-expression without FoTO1. We attach the relevant figure in the below (McClune et al., *Nature*. 2025 Jul 11;643(8071):582-592).

REDACTED

Fig. 3d, GC–MS TIC of *N. benthamiana* leaves transiently expressing the indicated genes. （McClune et al., 2025, Nature）

At present, Fernie's team, Yan's team and McClune's team have proposed three different biosynthetic pathways of baccatin III or paclitaxel respectively, It is now widely accepted in the scientific community that paclitaxel biosynthesis is not a simple linear process, but rather a complex and dynamically regulated network composed of multiple parallel (Liu et al., *Synth. Biol. J.* 2024, Apr 24, 5, 527-547; Shi et al., *Plant Commun.* 2025, Oct 13;6(10):101460; Yang et al., *Nat. Commun.* 2024, Mar 15;15(1):2339), so there is no only correct biosynthetic pathway. We believe that

more key genes or metabolic pathways will be discovered in the future, and we will continuously improve and update them in our subsequent studies. However, this manuscript focuses only on addressing the de novo reconstruction and stable expression of the paclitaxel biosynthetic pathway in the moss chassis.

3) As demonstrated by Liang et al. and McClune et al., the pathway steps proposed by Fernie were misassigned due to promiscuous background enzyme activities in tobacco cells that supported some conversions at very low levels. In the present manuscript, the authors do not validate each step independently, nor do they provide side-by-side comparisons of alternative pathway architectures. By reconstructing multiple steps simultaneously, this work is confounded by the same issue, background activity from moss enzymes.

Response:

We sincerely thank the reviewer for their valuable comment.

McClune et al. reported that co-expression of the previously identified genes *BAPT*, *PAM*, *PCL*, *T2'αOH*, and *DBTNBT* in tobacco, even in the presence of the substrate baccatin III, failed to produce detectable levels of paclitaxel (McClune et al., *Nature*. 2025 Jul 11;643(8071):582-592. As McClune et al. only gave the source of *DBTNBT* gene, but did not give the source and sequence of *BAPT*, *PAM*, *PCL* and *T2'αOH*, so it was impossible to judge whether the sequence used by McClune et al. was consistent with the gene we used, and the relevant excerpt from the text is provided as an image below.

REDACTED

Moreover, Fernie et al. co-expressed *BAPT*, *PAM*, *PCL*, *T2αOH* (*TB506*) and

*DBTNBT* in tobacco, and paclitaxel can be detected (Zhang et al., Mol. Plant. 2023 Dec 4;16(12):1951-1961). In our study, we co-expressed these five genes in *P. patens*, and paclitaxel can also be detected.

In Nature Synthesis, Liang et al. verified the enzyme groups BAPT, PAM, PCL, Tb506 and DBTNBT proposed by Fernie et al. Liang et al. proposed that two enzymes, DBTNBT, Tb506, could not produce the target product based on the substrate. However, DBTNBT can benzoylate N-debenzoyl-2'-deoxy-taxol to 2'-deoxy-taxol. The detection of paclitaxel from the co-expression of this enzyme set (BAPT, PAM, PCL, TB506 and DBTNBT) is attributed to background enzymes present in the tobacco chassis (Liang et al., *Nat. Synth.* 2025 April 30).

We individually removed TB506 and DBTNBT from the enzyme set consisting of BAPT, PAM, PCL, T2'αOH (TB506), and DBTNBT, followed by comparison with the control group (BAPT, PAM, PCL, TB506, DBTNBT). The results are shown in the figure below (Supplementary Fig. 10).

First, when both TB506 and DBTNBT were replaced with T2'OGD and T3'NBT simultaneously, compared with the control group (BAPT, PAM, PCL, TB506, DBTNBT), although the yield of paclitaxel increased, no significant doubling effect was detected, which indicates that both enzyme groups function independently.

Second, when TB506 and DBTNBT were individually removed from the enzyme set (BAPT, PAM, PCL, TB506, DBTNBT), the yield of paclitaxel significantly decreased compared to the control group. This indicates that both TB506 and DBTNBT are functionally involved in the paclitaxel biosynthetic pathway. Previous studies have also shown that overexpressing *DBTNBT* in *Taxus baccata* significantly increased the yield of paclitaxel (Perez-Matas et al., *Plant Biotechnol. J.* 2024 Sep 9;22, pp. 233-247). We speculate that there may be a branching step in the final stages of paclitaxel synthesis, rather than the sequential benzoylation followed by hydroxylation proposed by Liang et al. Alternatively, TB506 and DBTNBT may require the assistance of endogenous functional genes in plant chassis systems, such as *P. patens*. Therefore, it is not feasible to directly produce the target compound from

the substrate in yeast.

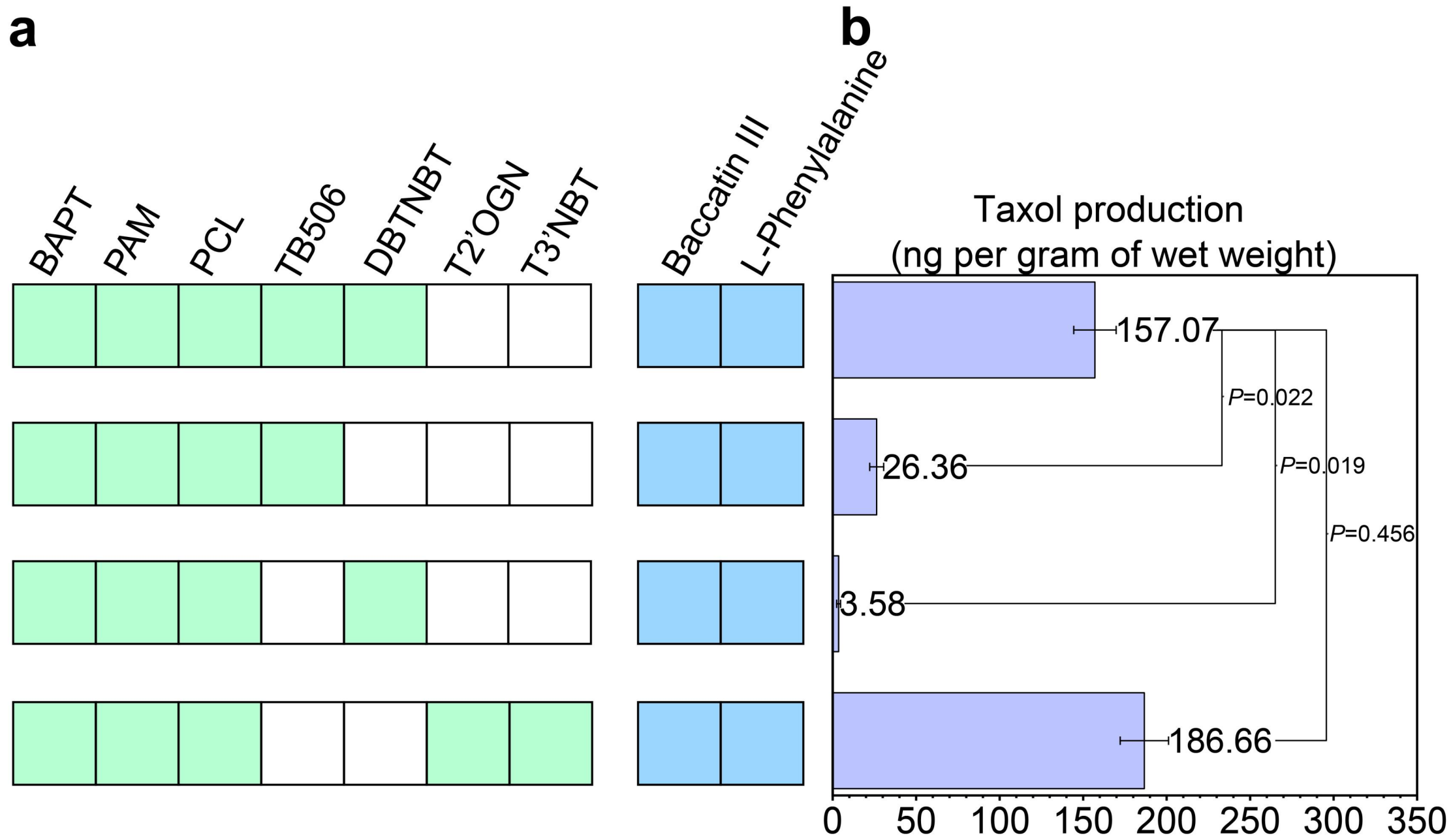


**Supplementary Fig. 10| Functional validation of TB506 and DBTNBT.**

(a) Schematic of enzyme combinations transiently expressed in *P. patens* protoplasts. Filled green squares denote overexpressed enzymes; filled blue squares indicate supplementation with the corresponding exogenous substrate (0.1 mM baccatin III and 1 mg/ml L-Phenylalanine). Blank squares represent the absence of the component. (b) Quantification of paclitaxel produced by the transient expression of corresponding enzyme combinations in (a). The one-way ANOVA *p*-values were used for significance difference analysis (*p*-values of 0.05 or less indicated statistical signifi cance). Data are mean ± SD (n = 3).

3) Publishing this outdated version of the pathway would propagate incorrect assumptions about enzyme functionality and pathway architecture, mislead future research efforts, and risk wasting resources in other laboratories. Establishing a moss platform for paclitaxel production has both research and commercial value, but this requires reconstructing the pathway in a stepwise manner, critically evaluating each step against the most recent findings, and supporting claims with robust evidence for intermediate identity (including NMR characterization where necessary). Achieving

this would essentially require repeating most of the reported work. For these reasons, the flaws cannot be remedied through revision, and I can only recommend rejection.

Response:

We sincerely thank the reviewer for their valuable comment.

We understand the reviewers' well-intentioned concern that publishing outdated metabolic pathways could mislead future researchers. Therefore, we have added a introduction of several recent studies on paclitaxel metabolic pathways (lines 51-82). Our additional experimental results indicate that these genes play an important role in moss [(Supplementary Fig. 9|Epoxidase is essential for baccatin III biosynthesis in the heterologous pathway.and Supplementary Fig. 10|Functional validation of TB506 and DBTNBT.)].In our discussion, we elaborated on our arguments and reviewed the currently reported diverse biosynthetic pathways of paclitaxel. Meanwhile, we do not believe these are outdated or incorrect genes, and hypothesize that there are alternative pathways supporting paclitaxel biosynthesis, and that the paclitaxel biosynthetic pathway is a dynamic network-like structure rather than a linear and exclusive route (Liu et al., *Synth. Biol. J.* 2024, Apr 24, 5, 527-547; Shi et al., *Plant Commun.* 2025, Oct 13;6(10):101460; Yang et al., *Nat. Commun.* 2024, Mar 15;15(1):2339). We propose that there are functional genes yet to be discovered within the paclitaxel biosynthetic pathway. Filling this knowledge gap in subsequent research will not only enrich the understanding of paclitaxel biosynthesis and provide more strategies for optimizing the heterologous production system of paclitaxel, but also help address the limitations of the yeast chassis. Furthermore, such findings would serve as a valuable reference for the analysis of biosynthetic pathways of other high-value natural products.

We sincerely appreciate the time and effort invested by the reviewers and the editorial team in evaluating our work. In particular, we appreciate Reviewer #3's detailed observations, which have helped us further strengthen the manuscript. Below we address each point raised.

## **Response to Reviewer #3**

I appreciate the additional experiments carried out by the authors. However, these experiments do not address my fundamental concerns regarding the validity of the results and the central claims of the manuscript. Careful examination of the data reinforces the conclusion that it is highly unlikely that paclitaxel is produced by the reconstructed pathway.

The problems in this study arise already at the very first step of the pathway and propagate throughout the entire reconstruction.

1. The pathway reconstruction fails at the initial step, namely the production of taxadiene (endotaxadiene) in moss. Upon expression of taxadiene synthase (TXS), the authors report the appearance of two peaks that are clearly distinct from the two peaks observed when TXS is expressed in *E. coli*, which serves as their control. The authors attribute this discrepancy to the formation of a taxadiene isomer (exotaxadiene), which they further suggest may be advantageous for pathway reconstruction.

This interpretation is not supported by the data. Expression of TXS in E. coli yields the two expected products, endotaxadiene and exotaxadiene, as confirmed by the MS spectra shown in Supplementary Figs. 1 and 7. These spectra match well with previously published MS data for these compounds (e.g., Zhao et al., J. Am. Chem. Soc. 2024, 146, 801−810; https://doi.org/10.1021/jacs.3c10864), and the relative retention times reported here are consistent with the known elution order of endotaxadiene and exotaxadiene. In contrast, the two peaks observed in moss do not correspond to either endotaxadiene or exotaxadiene based on both retention time and MS characteristics.

Therefore, the two compounds detected in moss are neither endotaxadiene nor exotaxadiene. They are most likely endogenous moss diterpenoids or aberrant products that do not possess the taxadiene skeleton. A plausible explanation for their appearance is the inappropriate choice of controls. To enable taxadiene biosynthesis, the authors co-express HMGR and a GGPPS to boost GGPP supply. However, their control consists of unmodified moss cells lacking these enzymes. Expression of HMGR and/or GGPPS in a plant system is expected to increase GGPP levels and consequently stimulate the production of endogenous diterpenoids. The observed peaks are therefore likely moss-derived diterpenoids produced as a result of enhanced GGPP availability, or potentially geranylgeraniol or geranyllinalool, which are known

hydrolysis products of GGPP and show MS features similar to those presented.

Appropriate controls, such as expression of HMGR alone, GGPPS alone, or their combination in the absence of TXS, are entirely missing and would have been essential to identify the origin of these compounds. An alternative possibility is that endotaxadiene or exotaxadiene, if produced at low levels, are rapidly modified by endogenous moss enzymes. In either case, the data do not support the conclusion that bona fide paclitaxel pathway intermediates are produced, casting serious doubt on the validity of the entire pathway reconstruction.

**Response:**

We appreciate the reviewer for raising this point. We apologize for the mistake.

Our initial GC/MS injection solution was neither purified nor concentrated. After purifying and concentrating the transiently or stably transformed materials via silica gel column chromatography, the following compounds were detected: taxa-4(20),11(12)-diene and taxa-4(5),11(12)-diene (Figure R1, R2), which are Fig. 2b and Supplementary Fig. 1; Fig. 2d and Supplementary Fig. 8 in the newly submitted manuscript, respectively. The description has been corrected in the revised manuscript (lines 156-161, 234-235, 703-704, 759-767).

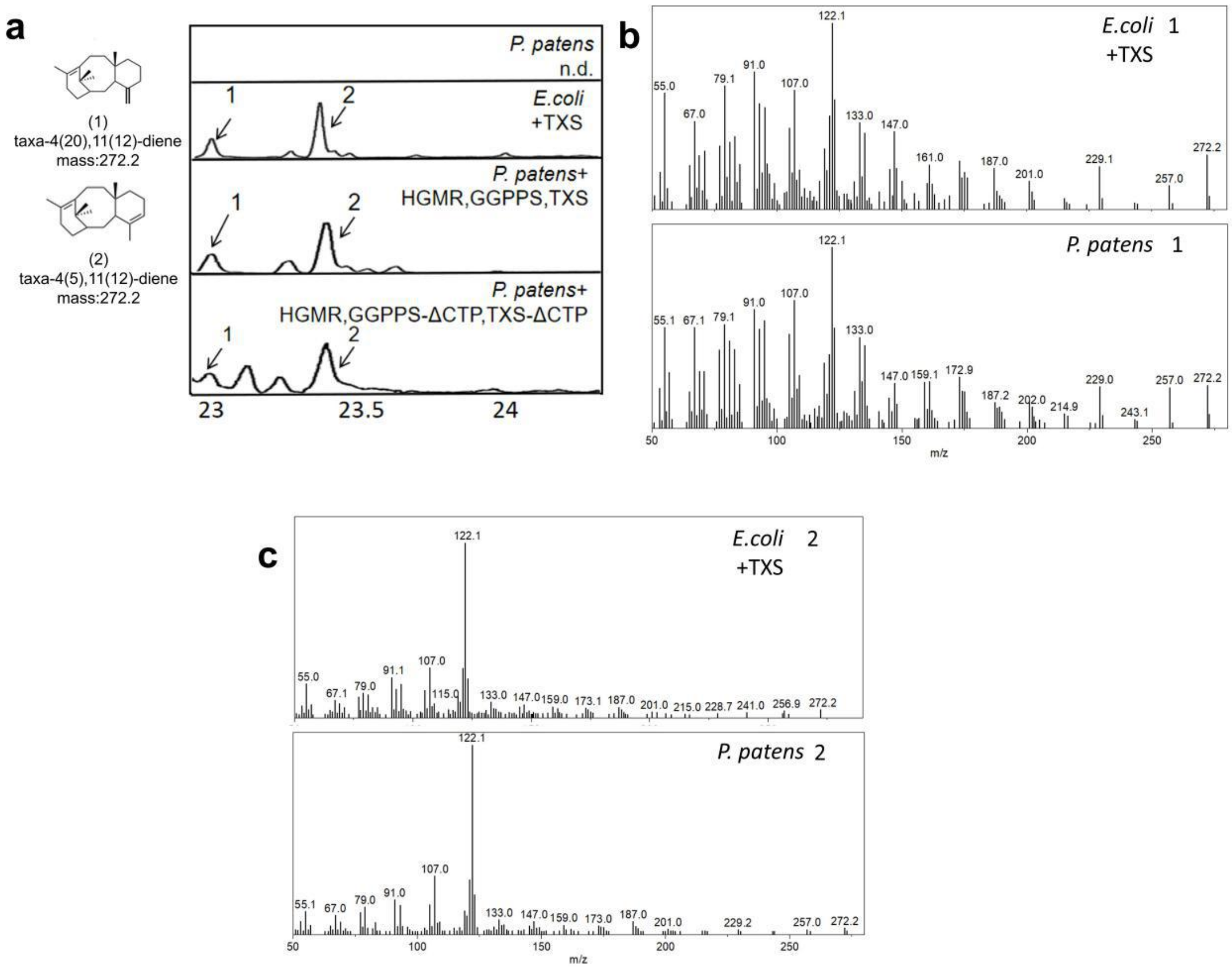


**Figure R1: Metabolite analysis of *P. patens* by gas chromatography-mass spectrometry (GC-MS).**

(a) Chromatogram, (b, c) mass spectrum of taxadiene and its derivatives in transiently transformed protoplasts. Taxadiene was detected in protoplasts overexpressing HMGR, GGPPS, and TXS (with transit peptide) or HMGR, GGPPS-ΔCTP, and TXS-ΔCTP (without transit peptide). Neither taxadiene was detected in wild-type protoplasts (n.d., not detected). (b) Taxadiene-4(20), 11(12)-diene. (c) Taxadiene-4(5), 11(12)-diene.

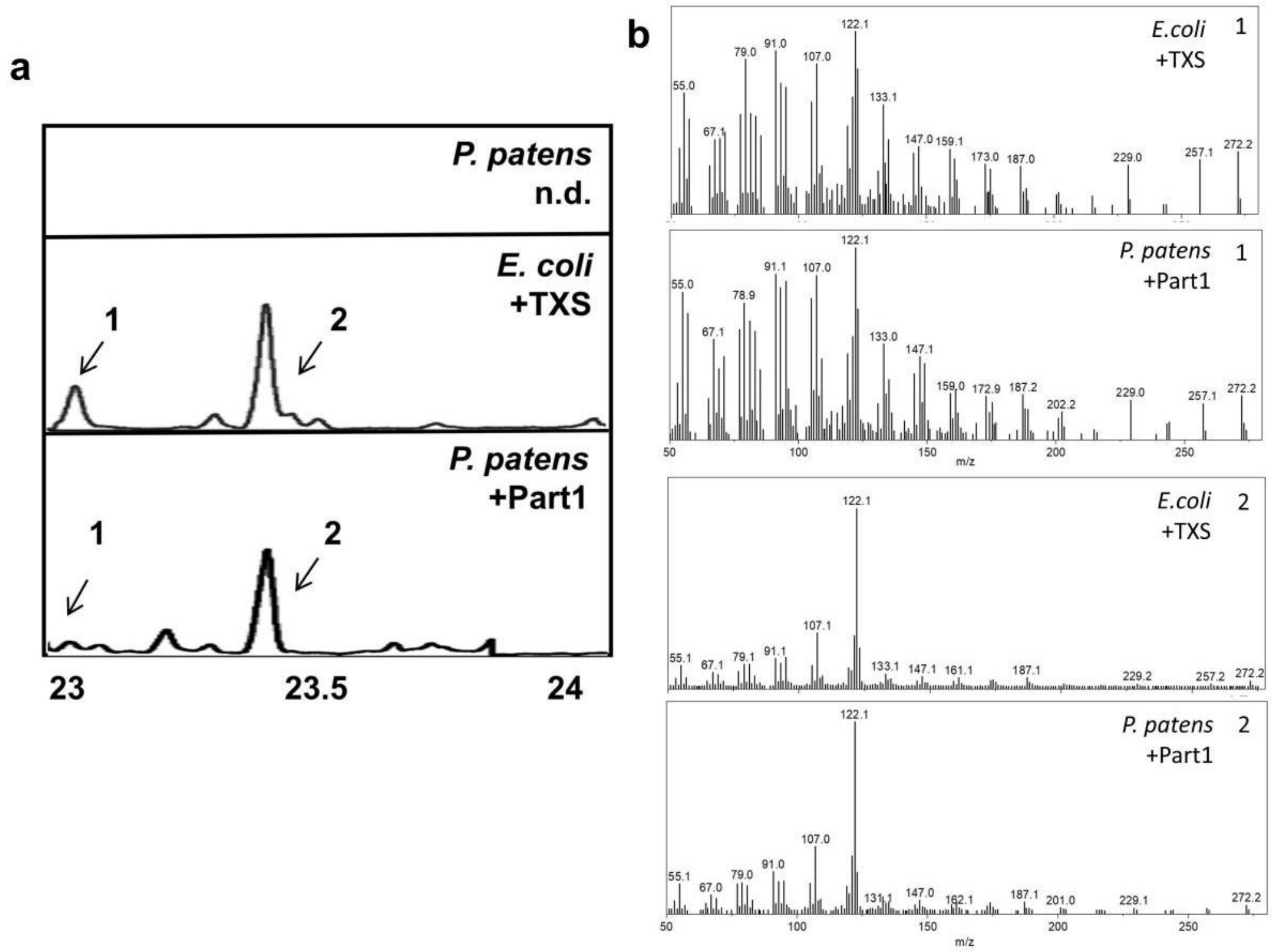


**Figure R2: Metabolite analysis of stable transgenic *P. patens* strains by GC-MS.**

(a) Chromatogram and (b) mass spectrum of taxadiene detected in Part 1 stable transgenic lines, compared to WT and taxadiene-producing *E. coli* positive control. n.d., not detected.

2. The problematic interpretation of the data continues at the next step, where the authors introduce the taxadiene-5α-hydroxylase (CYP725A4). The authors report the formation of taxadiene-5α-ol and provide an MS spectrum in support of this claim. However, comparison of this spectrum with published MS data for taxadiene-5α-ol demonstrates that this assignment is incorrect. The authors can refer, for example, to the supplementary data in McClune et al. (Nature 643, 582–592 (2025), https://doi.org/10.1038/s41586-025-09090-z), where the MS spectrum of taxadiene-5α-ol is reported and differs substantially from the spectrum shown here.

Thus, the compound detected is not taxadiene-5α-ol and cannot be considered a correct pathway intermediate. It is far more likely that this compound results from the activity of the hydroxylase on an endogenous moss diterpene or on the unrelated comp ounds observed in the previous step. Again, critical controls are missing. In particular, expression of HMGR and GGPPS together with the hydroxylase, but in the absence of TXS, would have revealed whether the detected compound originates from endogenous substrates rather than from taxadiene.

Taken together with the failure to demonstrate correct taxadiene production, this step provides further evidence that the pathway reconstruction is fundamentally flawed.

**Response:**

We appreciate the reviewer for raising this point.

Similarly, due to the lack of sample purification, impurity interference resulted in poor-quality mass spectra. Subsequently, we purified and concentrated the materials from both transient and stable transformations using silica gel column chromatography prior to GC-MS injection. This process led to the detection of three isomers: taxa-5α-ol, OCT, and iso-OCT (Figure R3 and R4), which are Fig. 2d and Supplementary Fig. 3; Fig. 4c and Supplementary Fig. 9 in the newly submitted manuscript, respectively. The description has been corrected in the revised manuscript (lines 181, 234-235).

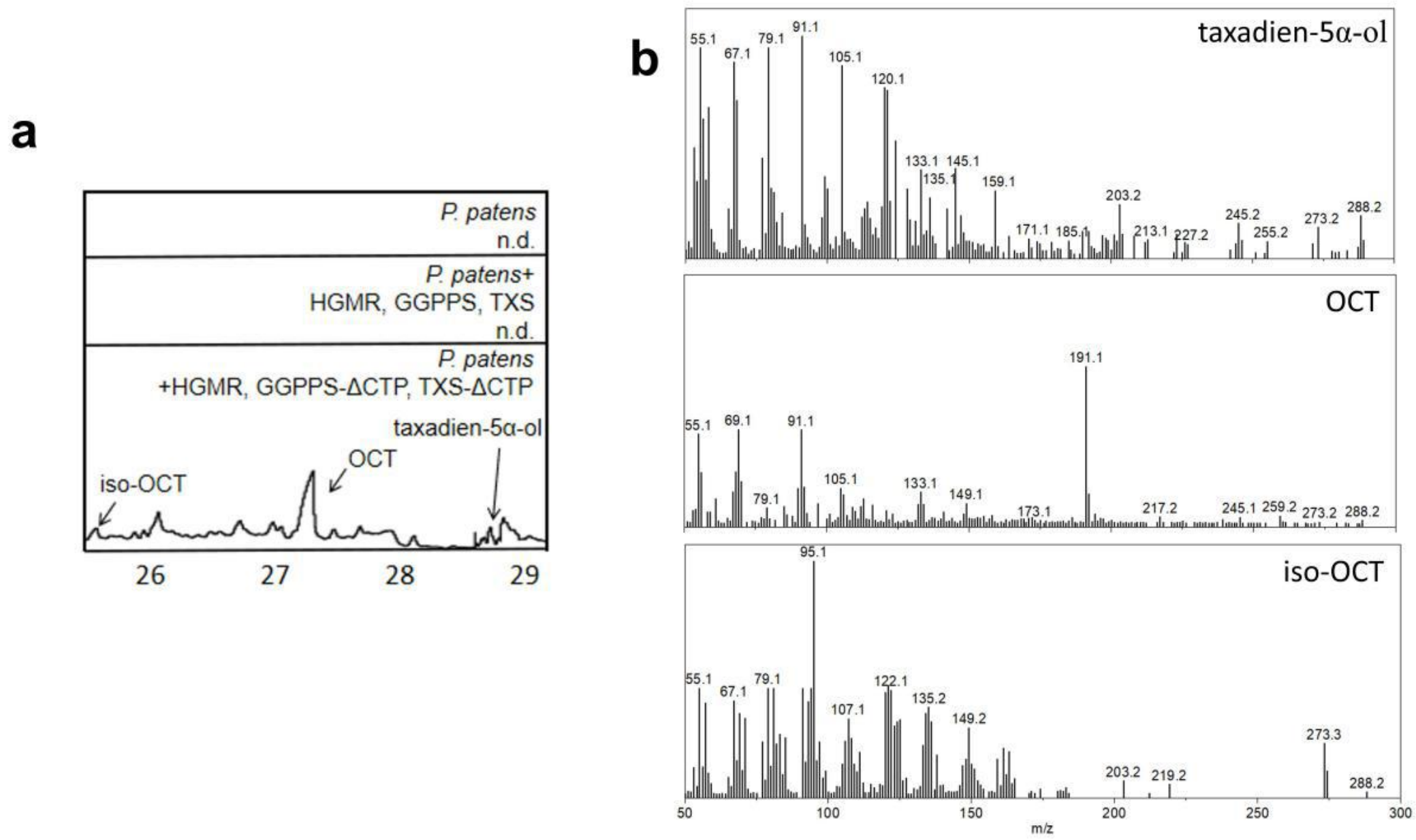


**Figure R3: Metabolite analysis of *P. patens* by GC-MS.**

(a) Chromatogram and (b) mass spectrum (m/z=288.20) of taxadiene produced in transiently transformed protoplasts. Taxadiene-5α-ol, OCT and iso-OCT were detected only in protoplasts overexpressing HMGR, GGPPS-ΔCTP, TXS-ΔCTP, and

T5αOH but not in those expressing HMGR, GGPPS, TXS, and T5αOH. Neither metabolite was detected in wild-type protoplasts (n.d., not detected).

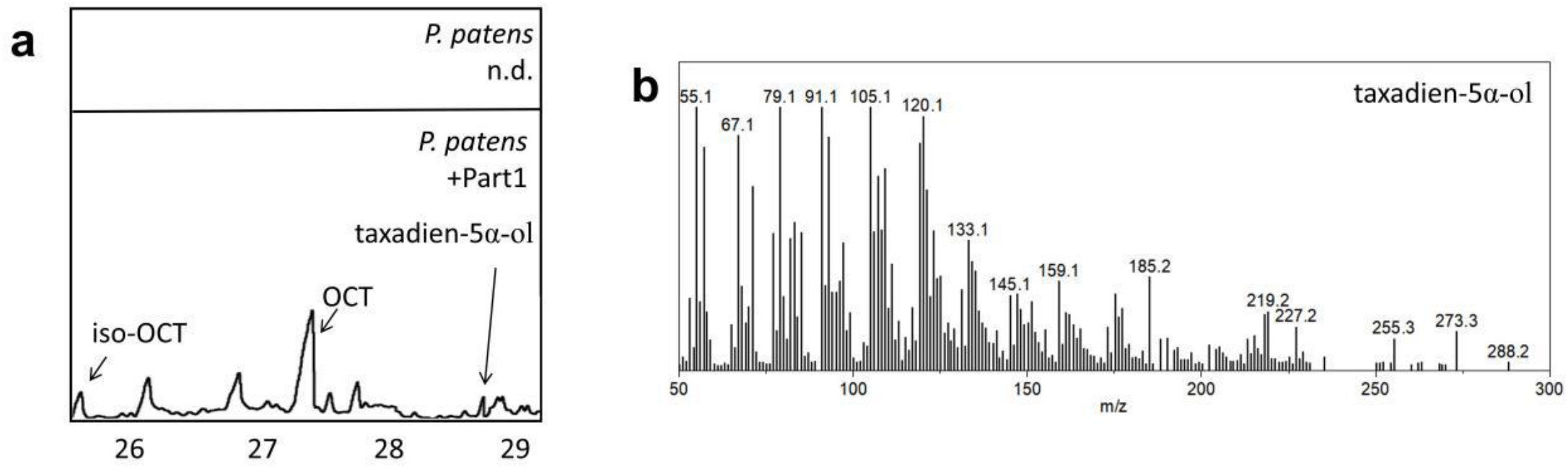


**Figure R4: Metabolite analysis of stable transgenic *P. patens* strains by GC-MS.**

(a) Chromatogram of taxadiene-5α-ol, OCT and iso-OCT were detected in Part 1 stable transgenic lines, compared to WT and taxadiene-producing *E. coli* positive control. n.d., not detected. (b) Mass spectrum (m/z=288.20) of taxadiene-5α-ol.

3. Following the first two problematic steps, it is not possible to assess or validate the function of any of the downstream enzymes. The authors introduce all remaining pathway genes simultaneously, without providing data for individual steps or appropriate enzyme-omission controls. No chromatograms are shown for intermediates corresponding to individual enzymatic reactions, and there is no validation of their identities.

Moreover, there is no evidence that the heterologously expressed enzymes are even produced in moss (e.g., by Western blot). Given the clear issues already identified at the earliest steps of the pathway (no production of taxadiene or taxadien-5a-ol), the absence of stepwise validation and expression controls makes it impossible to assign any observed metabolites to the intended enzymatic activities.

**Response:**

We appreciate the reviewer's interpretation.

The design logic of this study was not to identify the functions of unknown genes from scratch, but rather to validate whether the co-expression of genes with clearly

annotated functions (Sanchez-Muñoz et al., *Front. Bioeng. Biotechnol.* 2020 May 13;8:410; Walker et al., *Proc. Natl Acad. Sci. USA.* 2002 Jul 9;99(14):9166-9171; Xiong et al., *Nat. Plants*. 2021 Aug; 7, 1026-1036; Zhang et al., *Mol. Plant*. 2023 Dec 4;16(12):1951-1961) in *P. patens* could successfully synthesize paclitaxel. Since we have successfully detected the target product baccatin III and paclitaxel through co-expression, this directly demonstrates the overall functionality of this multi-gene pathway in *P. patens*. Therefore, conducting separate validation of each already well-characterized gene is neither necessary within the scope of this study nor consistent with conventional practices in the field—relevant studies typically consider the successful acquisition of the target product as sufficient validation of pathway functionality, without the need to redundantly verify individual gene functions that have been thoroughly documented in the literature (Yang et al., *Nat. Commun*. 2024, Mar 15;15(1):2339).

This study followed the conventional validation process in the field of natural product biosynthesis: direct detection and identification of the target product via liquid chromatography-mass spectrometry (LC-MS). Given that the target product has been successfully detected, this already constitutes key evidence demonstrating the functionality of the biosynthetic pathway. Therefore, we did not perform further protein-level validation of the related genes. This approach aligns with the standard practice in the field, where relevant literature typically prioritizes the presentation of metabolite detection data without requiring additional protein expression data (Ro et al., *Nature*. 2006 Apr 13; 440, 940-943; Zhang et al., *Nature*. 2022 Sep 8; 609, 341-347; Zhang et al., *Mol. Plant*. 2023 Dec 4;16(12):1951-1961).

4. Eventually, the authors claim the production of baccatin III and paclitaxel. However, the chromatographic and MS data provided in support of these claims are extremely limited and insufficient for compound identification. The chromatograms are narrowly cropped to show only a small retention time window around a single peak, and the MS spectra are similarly truncated to display only one major fragment ion, while omitting the diagnostically critical lower m/z region.

With such selectively presented data, it is not possible to confirm the identity of these compounds. To support these claims, the authors would need to present complete

chromatograms, including all relevant negative controls in which individual pathway genes are omitted, and extracted ion chromatograms for all diagnostic fragment ions of both standards and samples. Full, untrimmed MS spectra should be provided for all proposed intermediates and final products.
In the absence of these data, the reported detection of baccatin III and paclitaxel cannot be validated.

**Response:**

We appreciate the reviewer's interpretation.

The chromatographic and mass spectrometry data for baccatin III and paclitaxel are presented in accordance with the conventional format used in publicly available literature in the same field (Zhang et al., *Mol. Plant*. 2023 Dec 4;16(12):1951-1961). We provide the mass spectrometry data showing baccatin III and paclitaxel as ($[M+H]^+$) and ($[M+Na]^+$) adduct ions, rather than as a selective cropping of the original data. The relevant analyses have been validated through multiple parallel experiments, with the precursor and product ions showing good matches and reliable results.

To provide complete information, the full chromatographic and mass spectrometric plots corresponding to baccatin III and paclitaxel are now provided (Figures R6, R7, R9, R10). Additionally, to more clearly display the mass-to-charge ratios of the key fragment ions, corresponding locally enlarged plots are provided below (Figure R5, R8), which are Fig. 3b, 3d and Supplementary Fig. 4; Fig. 4d, 4e and Supplementary Fig. 10 in the newly submitted manuscript, respectively (lines 713, 724, 785 and 845).

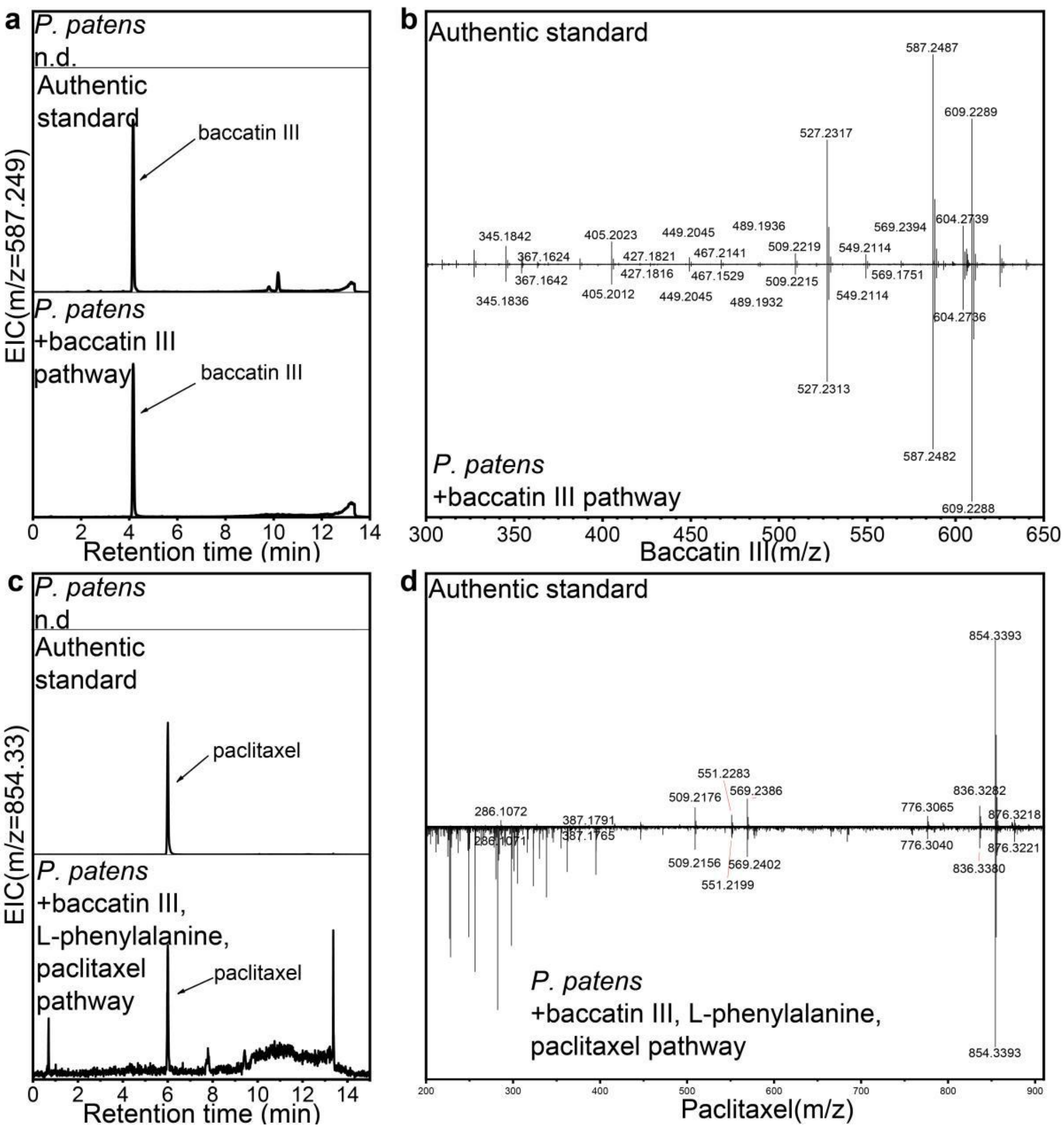


**Figure R5: Metabolite analysis of *P. patens* by UPLC-qTOF-MS. (Local mapping)**

(a) Chromatogram and (b) mass spectrum of baccatin III detected in *P. patens* protoplasts transiently expressing the baccatin III biosynthetic pathway, compared to wild-type (WT) and authentic standard. (c) Chromatogram and (d) mass spectrum of paclitaxel detected in *P. patens* protoplasts transiently expressing the complete paclitaxel biosynthetic pathway, compared to WT and authentic standard. n.d., not detected.

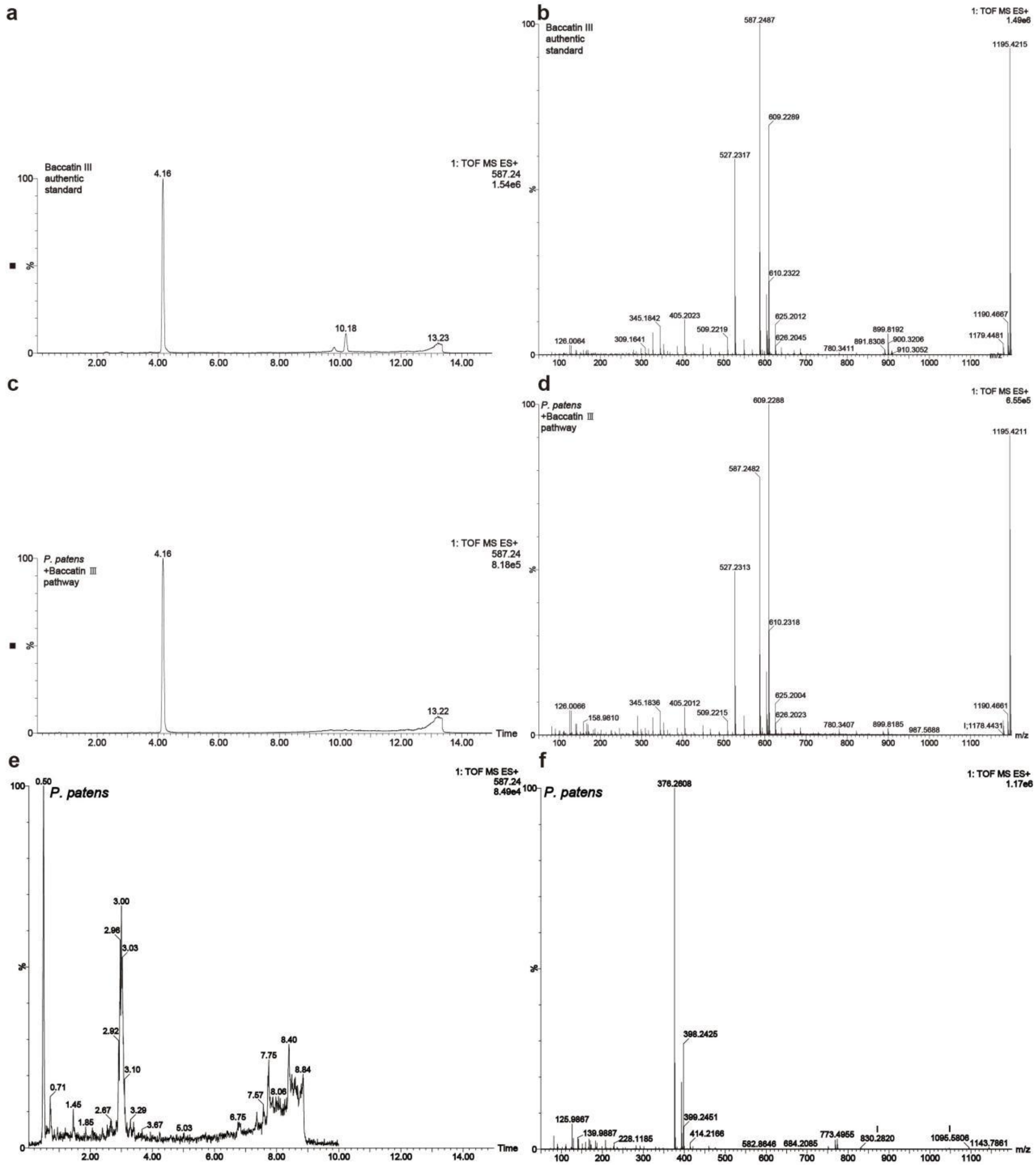


**Figure R6. The full images corresponding to Figure R5a and R5b.**

(a, c, e) Chromatogram and (b, d, f) mass spectrum of baccatin III detected in *P. patens* protoplasts transiently expressing the baccatin III biosynthetic pathway, compared to authentic standard and wild type of *P. patens*. (a, b) Baccatin III authentic standard. (c, d) *P. patens* protoplasts transiently expressing the baccatin III biosynthetic pathway. (e, f) Wild type of *P. patens*.

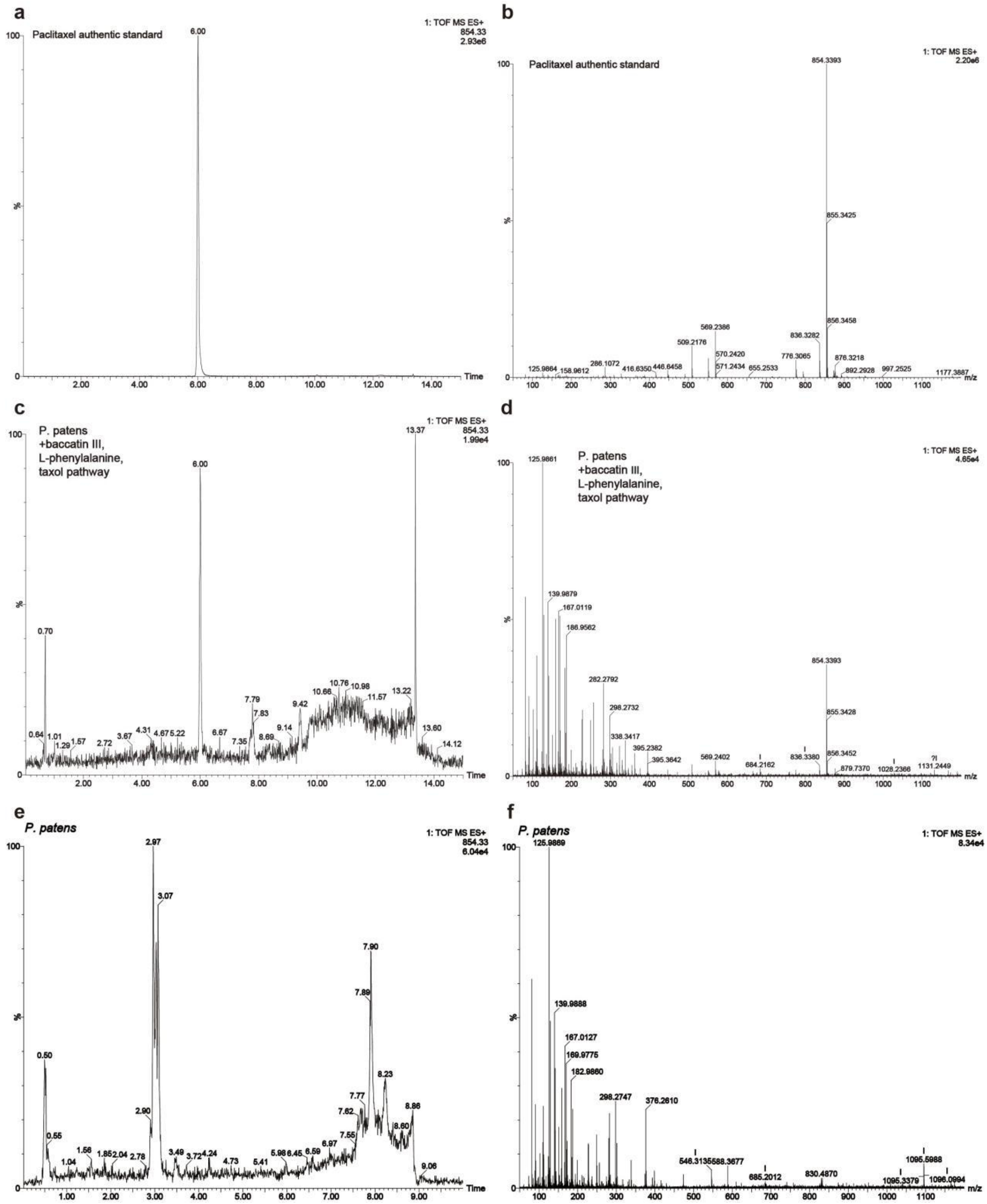


**Figure R7. The full images corresponding to Figure R5c and R5d.**

(a, c, e) Chromatogram and (b, d, f) mass spectrum of paclitaxel detected in *P. patens* protoplasts transiently expressing the complete paclitaxel biosynthetic pathway, compared to authentic standard and wild type of *P. patens*. (a, b) Paclitaxel authentic standard. (c, d) *P. patens* protoplasts transiently expressing the complete paclitaxel biosynthetic pathway. (e, f) Wild type of *P. patens*.

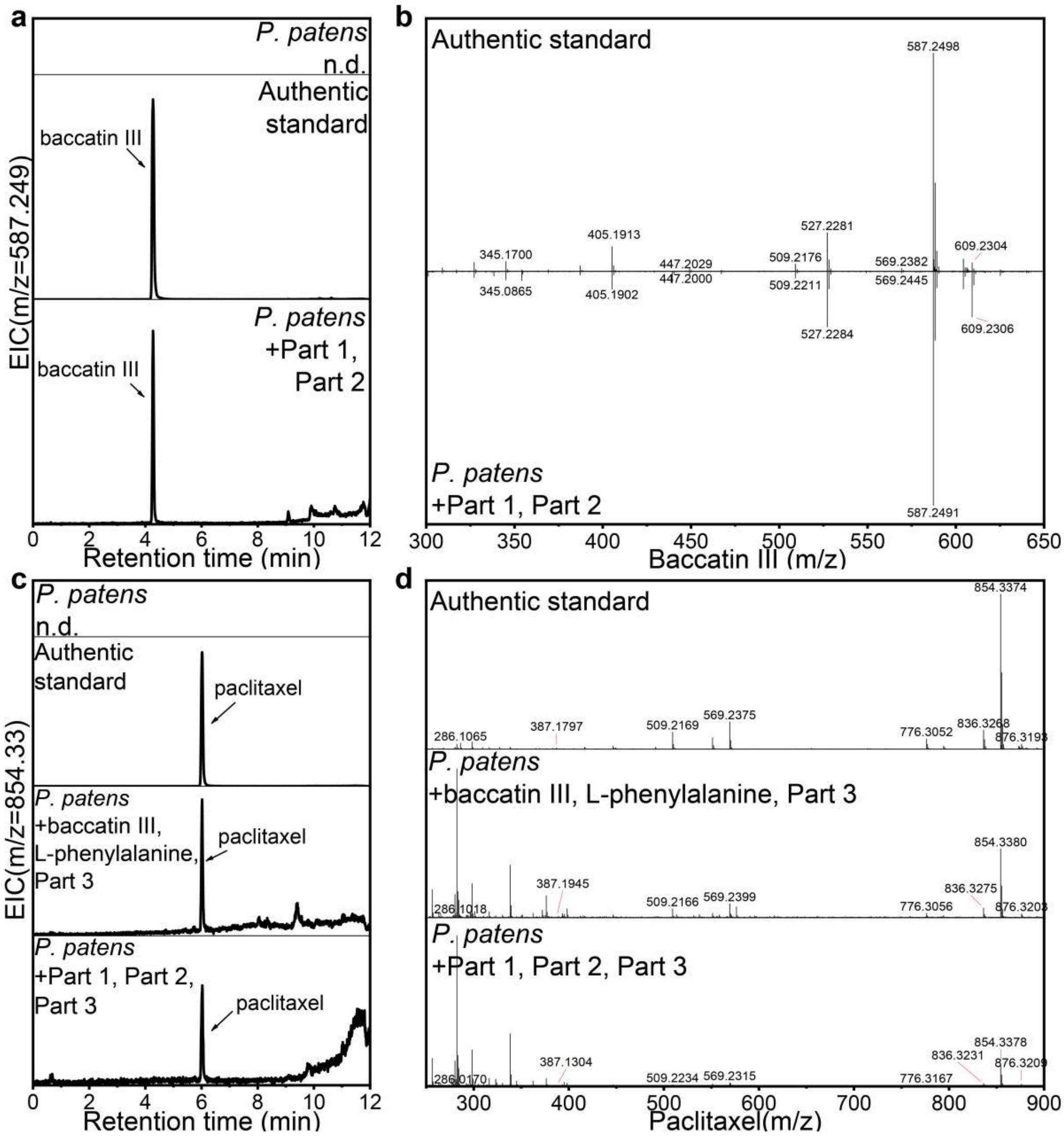


**Figure R8. Metabolite analysis of stable transgenic *P. patens* strains by UPLC-qTOF-MS. (Local mapping)**

(a)Chromatogram and (b) mass spectrum of baccatin III detected in Part 1/2 stable transgenic lines, compared to WT and authentic standard. (c) Chromatogram and (d) mass spectrum of paclitaxel detected in Part 3 and Part 1/2/3 stable transgenic lines, compared to WT and authentic standard. n.d., not detected.

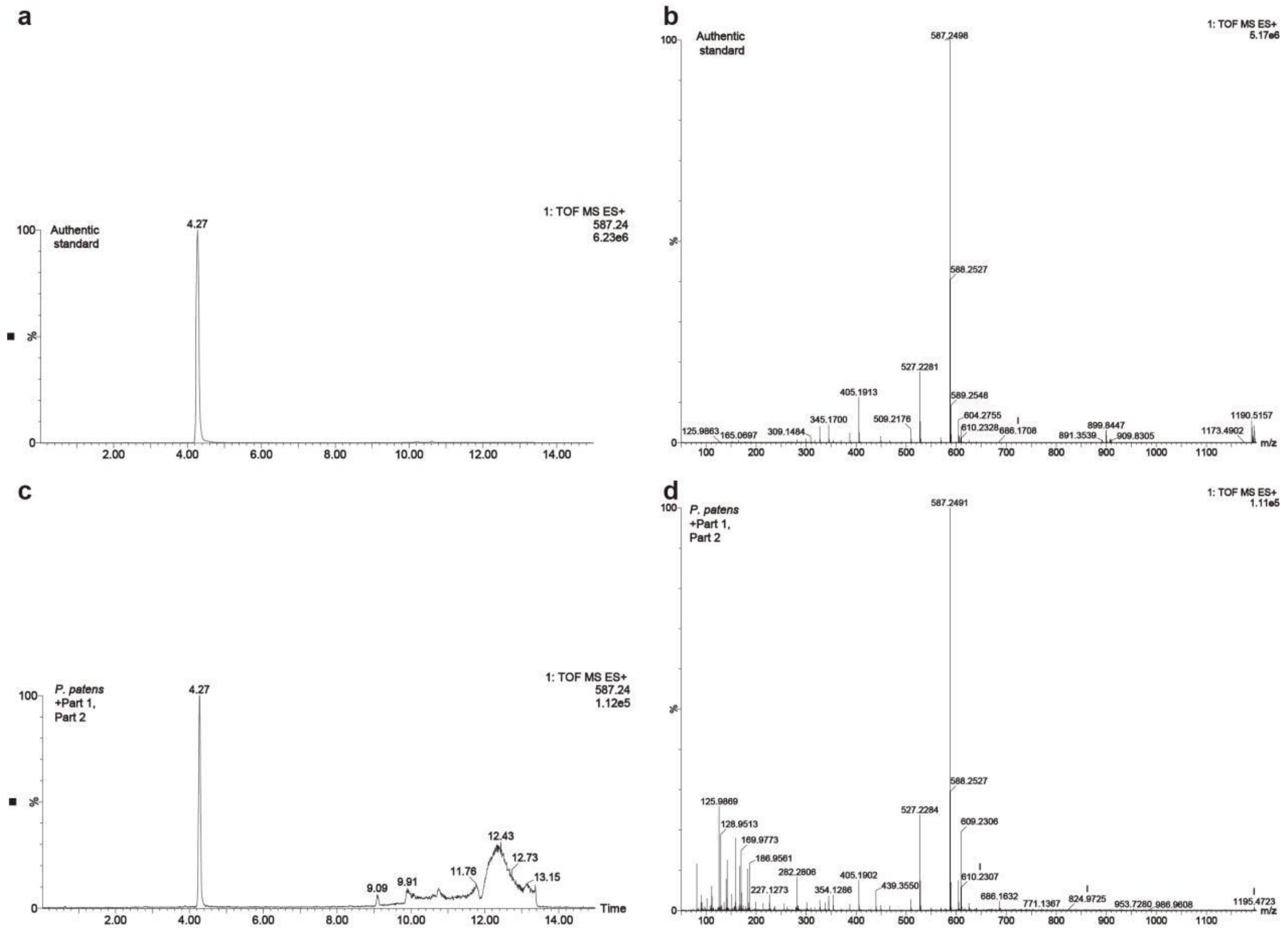


**Figure R9. The full images corresponding to Figure R8a and R8b.**

(a, c) Chromatogram and (b, d) mass spectrum of baccatin III detected in Part 1/2 stable transgenic lines, compared to authentic standard. (a, b) Baccatin III standard. (c, d) Part 1/2 stable transgenic lines.

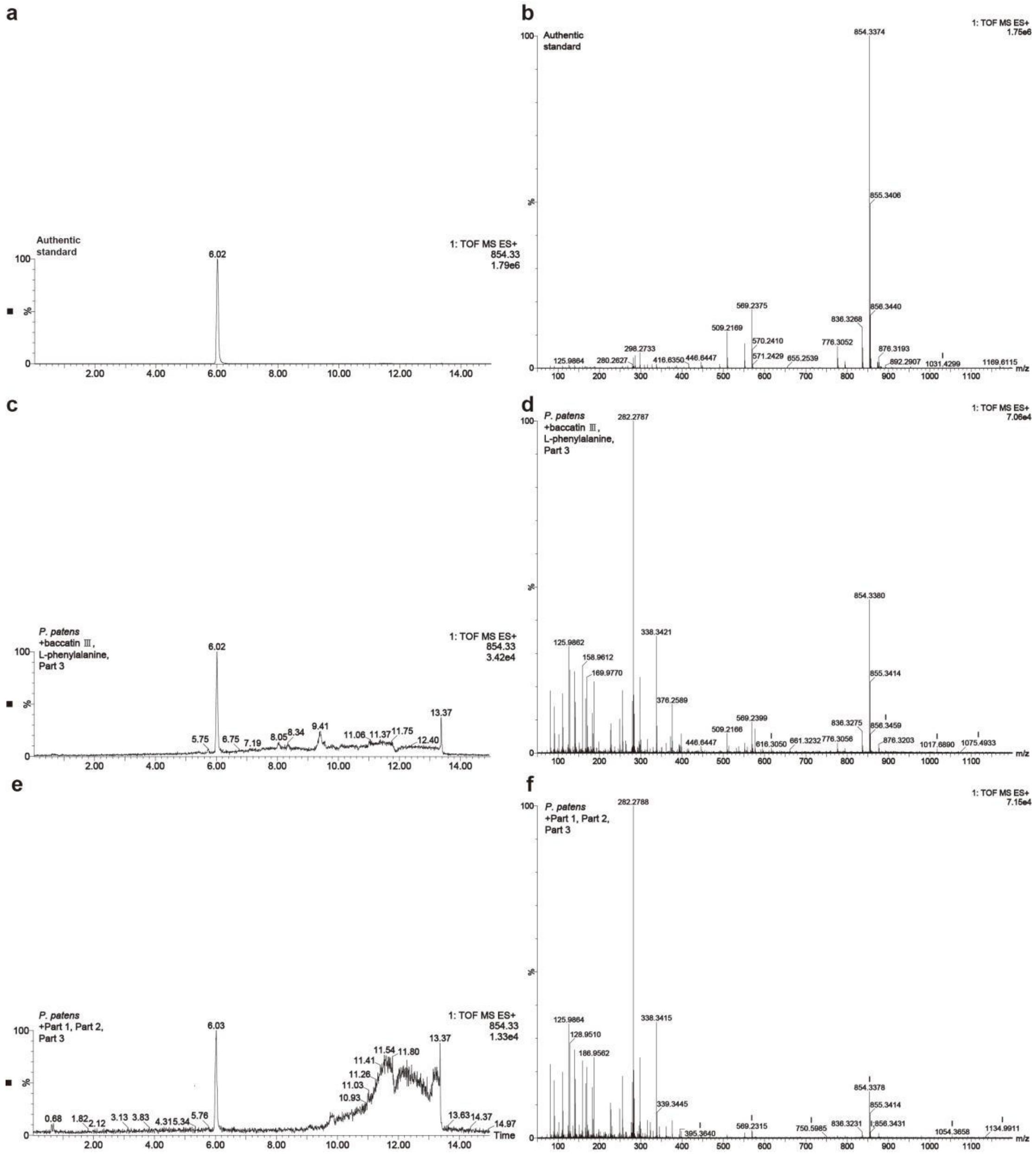


**Figure R10. The full images corresponding to Figure R8c and R8d.**

(a, c, e) Chromatogram and (b, d, f ) mass spectrum of paclitaxel detected in Part 3, Part1/2/3 stable transgenic lines, compared to authentic standard. (a, b) Paclitaxel standard. (c, d) Part 3 stable transgenic lines cultured with baccatin III and L-phenylalanine. (e, f) Part1/2/3 stable transgenic lines.

5. The substrate feeding experiments are critically undermined by the fact that baccatin III preparations are not chemically pure and are well known to contain additional taxanes, including paclitaxel itself. Consequently, the detected paclitaxel signal could arise from trace paclitaxel contamination in the baccatin III preparation, or from the activity of one or more introduced enzymes on other taxanes or pathway intermediates present in the fed substrate, rather than from de novo paclitaxel biosynthesis.

Importantly, in the revised manuscript, paclitaxel is detected even when baccatin III is supplied to cells expressing an incomplete set of pathway enzymes (Supplementary Fig. 10, second and third samples from the top). Under these conditions, paclitaxel biosynthesis should not be possible, as essential enzymatic steps are absent. The detection of paclitaxel in these samples therefore strongly suggests that the observed signal originates either from paclitaxel already present in the supplied substrate, from enzymatic modification of contaminating intermediates, or that the compound assigned as paclitaxel has been misidentified.

**Response:**

We appreciate the reviewer's interpretation.

First, we verified the standard sample of baccatin III by HPLC and Q-TOF LC/MS analyses, and did not detect paclitaxel (Figures R11 and R12).

Furthermore, we have engineered strains capable of the complete de novo biosynthesis of paclitaxel, with no exogenous substrates added, yet paclitaxel was still successfully detected Figure R8c and 8d, which are Fig. 4d, 4e and Supplementary Fig. 10 in the newly submitted manuscript.

Thirdly, in Supplementary Fig. 10 (Figure R13, which is Supplementary Fig. 11 in the newly submitted manuscript), the second and third samples from the top correspond to the combination without DBTNBT (BAPT, PAM, PCL, TB506; the second sample from the top) and the combination without TB506 (BAPT, PAM, PCL, DBTNBT; the third sample from the top), respectively. Since the quantitative detection result of paclitaxel in the combination without TB506 did not fall within the confidence interval, we further performed qualitative detection of the transient expression products from the combination without DBTNBT and the combination without TB506 using Q-TOF LC/MS. The results showed that paclitaxel could be detected in

the combination without DBTNBT, whereas it could not be detected in the combination without TB506. Using *P. patens* as the chassis cell, the above results indicate that TB506 is essential for the biosynthesis of paclitaxel, while also suggesting that *P. patens* may contain an enzyme with a function similar to that of DBTNBT.

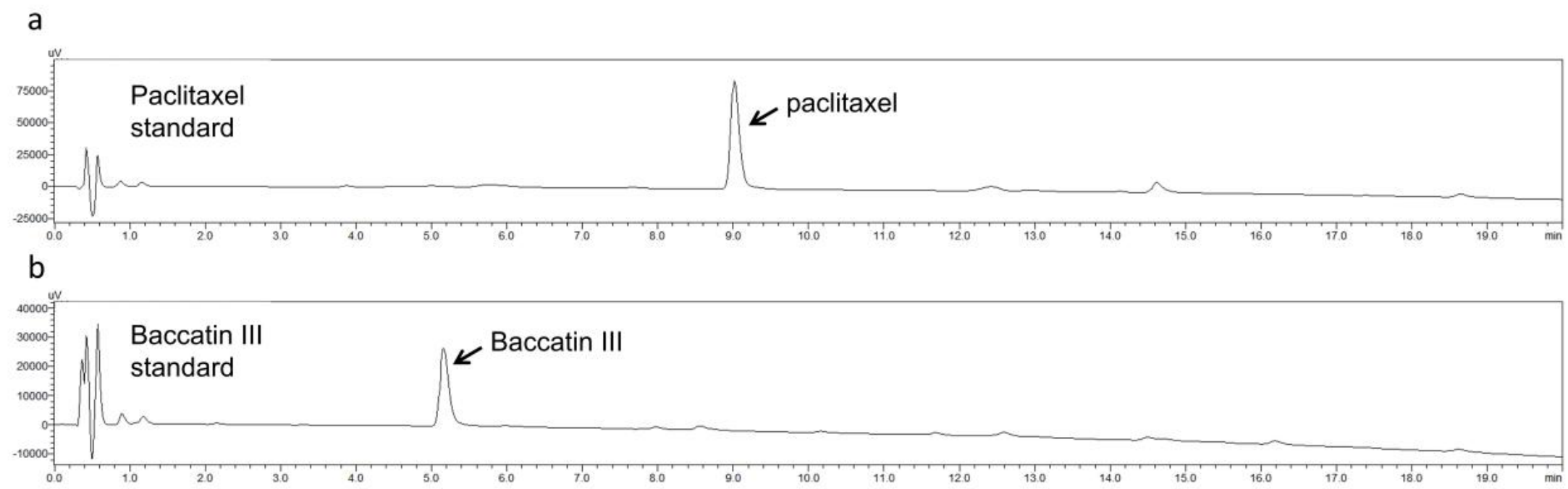


**Figure R11. Detection of paclitaxel in the standard of baccatin III via HPLC.**

(a) Baccatin III standard. (b) Paclitaxel standard.

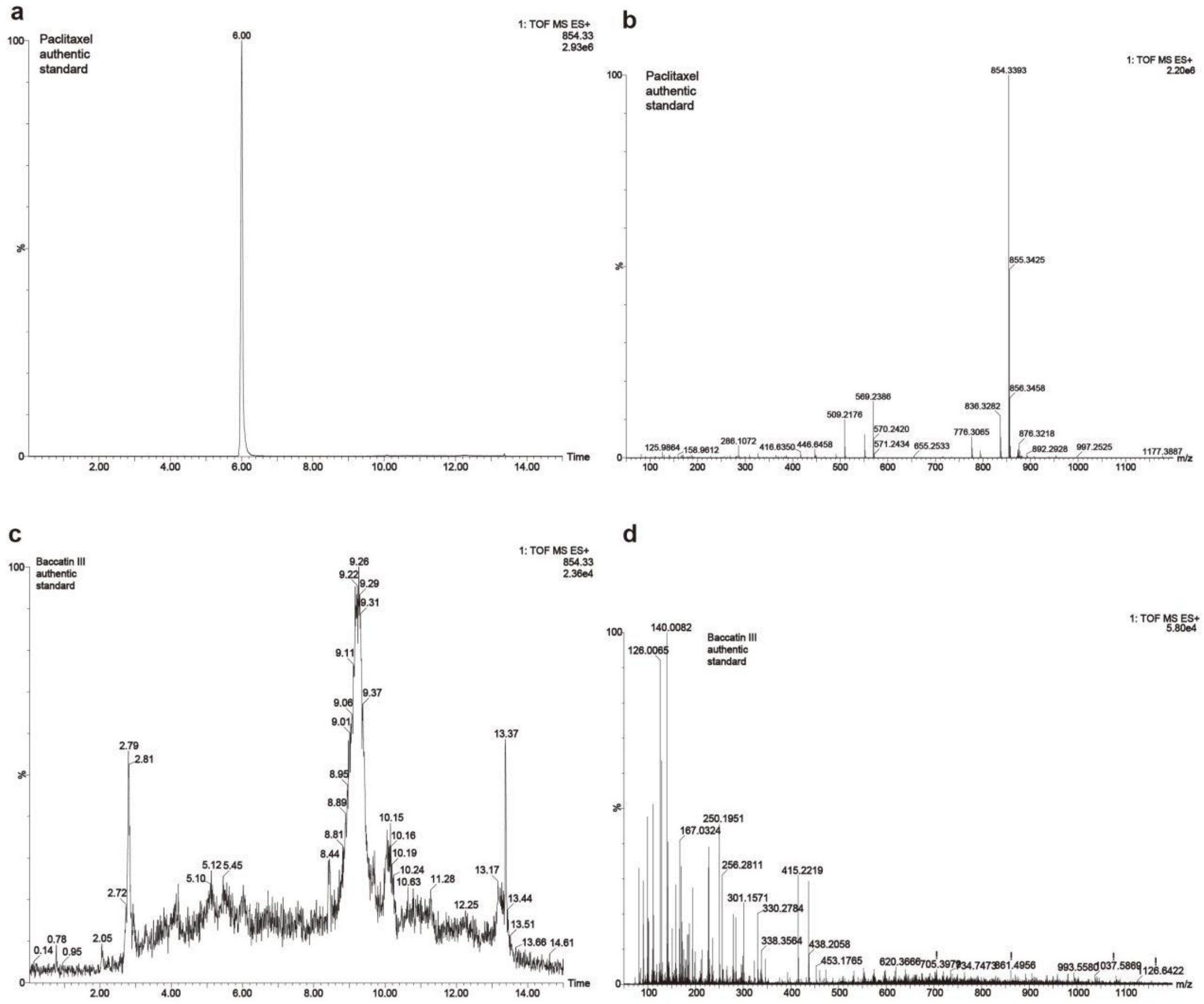


**Figure R12. Detection of paclitaxel in the standard of baccatin III via Q-TOF LC/MS.**

(a, c) Chromatogram and (b, d) mass spectrum of the standard of baccatin III, compared to paclitaxel standard.

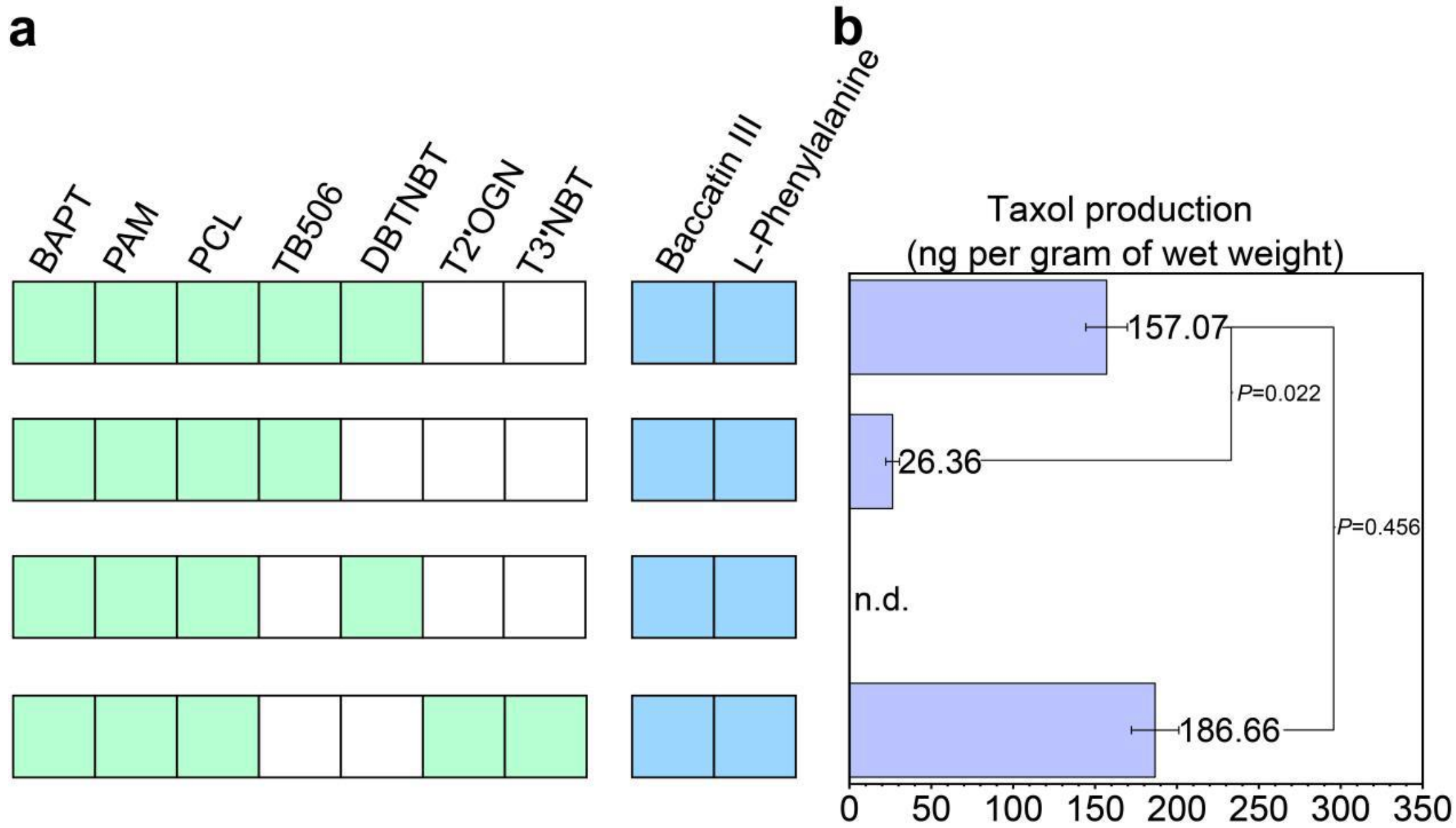


**Figure R13. Functional validation of TB506 and DBTNBT.**

(a) Schematic of enzyme combinations transiently expressed in *P. patens* protoplasts. Filled green squares denote overexpressed enzymes; filled blue squares indicate supplementation with the corresponding exogenous substrate (0.1 mM baccatin III and 1 mg/ml L-Phenylalanine). Blank squares represent the absence of the component. (b) Quantification of paclitaxel produced by the transient expression of corresponding enzyme combinations in (a). The one-way ANOVA p-values were used for significance difference analysis (*p*-values of 0.05 or less indicated statistical significance). Data are mean ± SD (n = 3). n.d., not detected.

6. The study also fails to address key discrepancies between the results and the literature. For example, for their reconstruction, the authors utilize the 9a-hydroxylase CYP725A22-1/CYP725A37. It is well established that this CYP requires the presence of an acetyl group at the C-13 of the taxadiene skeleton to functon(McClune et al., Nature 643, 582–592 (2025). https://doi.org/10.1038/s41586-025-09090-z; Yang et al., Nat Commun 15, 2339 (2024). https://doi.org/10.1038/). There is no C-13 acetyltransferase in the utilized enzyme set. How are then baccatin III or paclitaxel produced? Even if a moss enzyme could non-specifically catalyze this step, the added C-13 acetyl group would have to be subsequently removed to produce baccatin II and to allow the attachment of the C-13 side chain to eventually synthesize paclitaxel. Neither acetylation nor deacetylation at C-13 is accounted for or discussed. This omission represents a major gap in the pathway the authors are aiming to reconstruct.

**Response:**

We appreciate the reviewer for raising this point.

The 9α-hydroxylase employed in this study is CYP725A22-1 (Zhang et al., *Mol. Plant*. 2023 Dec 4;16(12):1951-1961), which is distinct from CYP725A37 used in the studies by McClune et al. and Yang et al. (McClune et al., *Nature*. 2025 Jul;643(8071):582-592). Furthermore, the reaction sequence involving CYP725A22-1 reported by Zhang et al. differs from that described by McClune et al. Zhang et al. proposed that C9 hydroxylation occurs at an earlier stage of synthesis (Figure R14), contrary to the sequence reported by McClune et al. This discrepancy may imply differences in the catalytic mechanisms.

Finally, based on the transient expression system of tobacco, Zhang et al. successfully synthesized baccatin III using a reshuffled gene set. Similarly, using the gene combination, we achieved de novo biosynthesis of both baccatin III and paclitaxel via transient or stable expression. These results collectively provide robust evidence that this gene set exhibits complete catalytic functionality and pathway compatibility within our research system.

REDACTED

**Figure R14. The pathway of paclitaxel biosynthesis.** (Zhang et al., *Mol. Plant*. 2023

Dec 4;16(12):1951-1961)

The catalytic sequence of seven hydrogenation steps is unclear. The black dotted line and the red enzyme represent newly identified steps. A black color represents previously identified steps. Module 1 represents baccatin III biosynthesis. Module 2 represents the second part of the pathway. TXS, taxadiene synthase; T5αOH, taxane 5α-hydroxylase; TAT, taxadiene-5α-ol-O-acetyl transferase; T10βOH, taxane 10β-hydroxylase; T13αOH, taxane 13α-hydroxylase; T2αOH, taxane 2α-hydroxylase; T9αOH, taxane 9α-hydroxylase; T7βOH, taxane 7β-hydroxylase; T1βOH, taxane 1β-hydroxylase; TBT, taxane-2α-O-benzoyltransferase; DBAT, 10-deacetyl baccatin III-10-O-acetyltransferase; BAPT, baccatin III-3-amino, 13-phenylpropanoyl -transferase; T20αOH, taxane 20α-hydroxylase; DBTNBT, 30-N-debenzoyl-20-deoxytaxol-N-benzoyltransferase; PAM, phenylalanine aminomutase; PCL, β-phenylalanine coenzyme A ligase.

7. In addition to the above, my earlier concerns about essential pathway components, like FoTO1, TOT1, T7dA, and T9dA, missing from the reconstruction, were not adequately addressed. Mentions to the relevant publications were added in the text, but their functional significance for the pathway was neither discussed nor experimentally validated.
Taken together, the key concerns raised above propagate from the earliest steps to the full pathway and are fundamental for the study. In my view, the data far from support the manuscript's central conclusion, and resolving these problems would require a fundamentally different experimental and analytical approach rather than revision of the current study.

**Response:**

We appreciate the reviewer for raising this point.

In studies on heterologous synthesis systems of paclitaxel, the essentiality of key enzyme genes is constrained by specific pathway design and host background. Current research indicates that the paclitaxel biosynthetic pathway exhibits dynamic and network-like characteristics rather than a single linear route (Liu et al., *Synth. Biol. J*. 2024, Apr 24, 5, 527-547; Shi et al., *Plant Commun*. 2025, Oct 13;6(10):101460; Yang et al., *Nat. Commun*. 2024, Mar 15;15(1):2339). Consequently, certain genes are

not indispensable during pathway reconstruction.

The specifics are outlined as follows:

1. FoTO1 functions is a nuclear transport factor 2 (NTF2)-like protein, rather than a catalytic enzyme. Its absence does not affect the production of taxadiene-5α-ol, and although it contribute to increased yield, its precise mechanism remains unclear (McClune et al., *Nature*. 2025 Jul;643(8071):582-592). Therefore, it is not essential in the reconstruction of enzymatic catalytic pathways.

2. TOT1 has not been utilized in multiple biosynthetic systems for paclitaxel precursors. For instance, Zhang et al. (2023) successfully achieved the de novo synthesis of baccatin III without introducing TOT1 in a tobacco transient expression system (Zhang et al., *Mol. Plant*. 2023 Dec 4;16(12):1951-1961). Similarly, Yang et al. did not employ TOT1 in their study, yet accomplished the biosynthetic pathway from taxa-4(20),11(12)-diene-5α-ol to 1β-dehydroxybaccatin VI (Yang et al., *Nat. Commun*. 2024, Mar 15;15(1):2339).

3. Similarly, in the transient expression platform in tobacco published by Jiang et al. (2024) in Science, T7dA and T9dA were also excluded from the system, yet the de novo synthesis of baccatin III was still successfully accomplished (Jiang et al., *Science*. 2024 Feb 9;383(6683):622-629). Even they only used nine genes to co-express to produce baccatin III (Jiang et al., *Science*. 2024 Feb 9;383(6683):622-629).

In summary, FoTO1, TOT1, T7dA, and T9dA are non-essential genes in existing successful synthesis cases. Our research results indicate that these genes are non-essential for the production of paclitaxel in our chassis cell. This phenomenon further supports the redundancy and functional substitutability of the paclitaxel biosynthetic pathway.

We have been working on the construction of the paclitaxel engineered strain since 2023. This gene combination has been verified through multiple repeated experiments with consistent and reliable results. During this process, if we find that the gene set we are using fails to achieve the biosynthesis of paclitaxel in chassis cell *P. patens*, we will certainly proceed with stepwise validation and analyze the underlying reasons.

Meanwhile, we fully acknowledge the contribution of the gene combination reported by McClune et al. in improving paclitaxel production efficiency. In the Abstract, Introduction, Results, and Discussion (lines 11-14, 127-131, 253-271, 304-308, 328-330, 699-701, 742-749 in the newly submitted manuscript), we have acknowledged their contributions to the elucidation of the paclitaxel biosynthetic pathway of McClune et al., as well as the roles played by the relevant genes in the optimization of the engineered paclitaxel strain in this study.

After successfully constructing the paclitaxel engineering strain, we promptly carried out systematic optimization efforts and incorporated genes beneficial for improving paclitaxel yield, such as those reported by researchers McClune and Liang into subsequent optimization studies, which led to a significant increase in the yield of paclitaxel (Figure R15), which are Fig. 5 in the newly submitted manuscript (lines 253-271, 742-749).

Furthermore, we will subsequently use more gene sets reported by McClune et al to optimize the paclitaxel engineering strain and enhance the yield of paclitaxel.

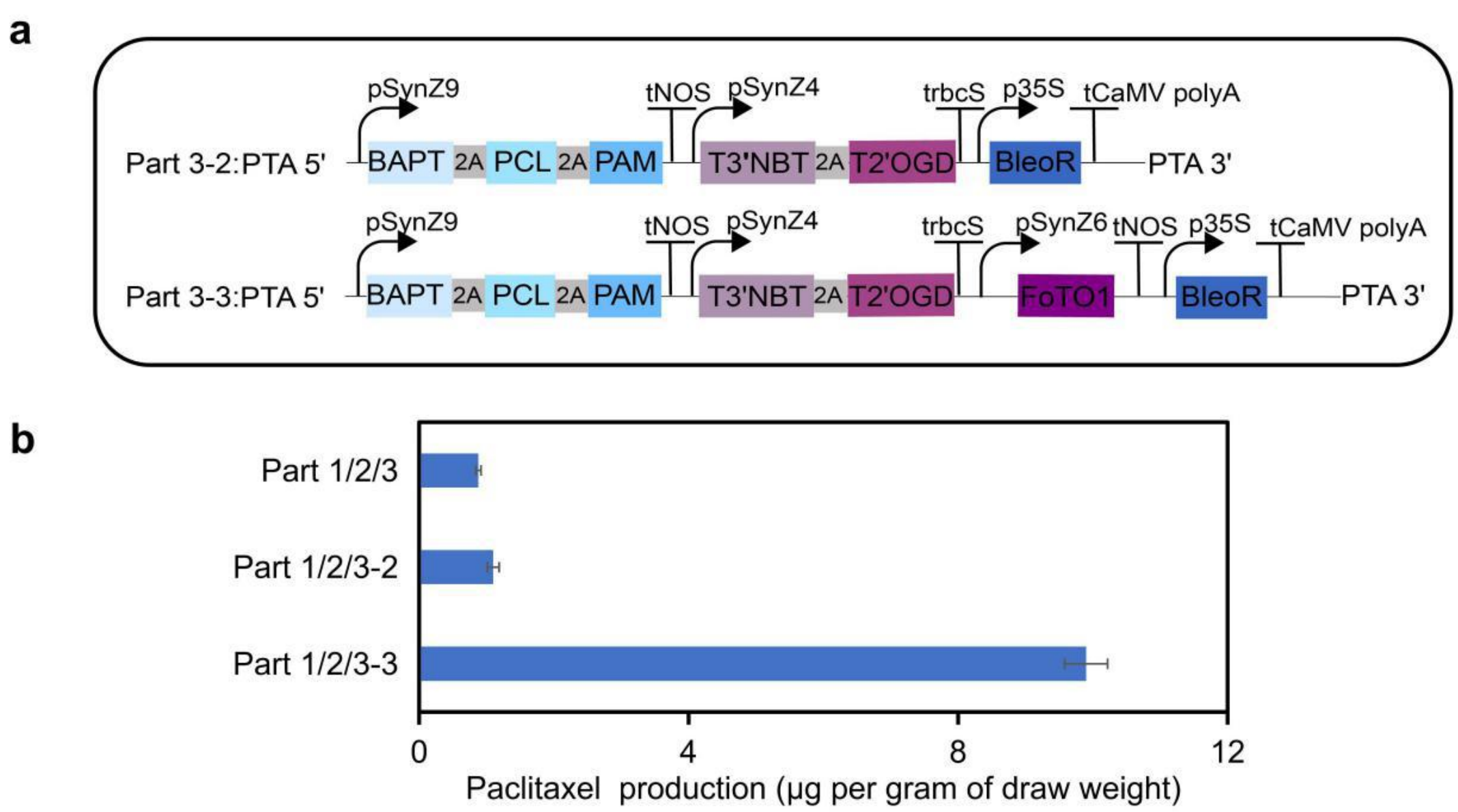


**Figure R15. Optimization and Detection of the paclitaxel Engineered Strain.**

(a) Schematic diagram of the modification of the third part of the heterologous gene module. Part 3-2 (BAPT, PAM, PCL, T2'OGD and T3'NBT). Part 3-3 (BAPT, PAM,

PCL, T2'OGD, T3'NBT, FoTO1). 2A: 2A self-cleaving peptides. Selection markers: BleoR (zeocin resistance). (b) Detection of paclitaxel production in stable transgenic lines harboring the corresponding gene modules using QTRAP 6500+ low mass mode.

We sincerely appreciate the time and effort invested by the reviewers and the editorial team in evaluating our work. Below we address each point raised.

## Response to Reviewer #3

The revised manuscript and rebuttal do not resolve my central concerns. On the contrary, several of the experiments provided in the revision reinforce the concerns raised in the previous rounds of review. The manuscript claims "de novo reconstruction of the paclitaxel biosynthetic pathway in moss." However, the revised data instead suggest that paclitaxel formation in these experiments depends on a fortuitous combination of introduced taxane biosynthetic genes and unidentified endogenous P. patens activities. This is a fundamentally different conclusion: paclitaxel production in this system is a fortuitous outcome, not the result of legitimate pathway reconstruction.

**Response:**

We are thankful for the reviewer's question. Our manuscript claims "de novo reconstruction of the paclitaxel biosynthetic pathway in moss". The key point is that this pathway can synthesize paclitaxel de novo in moss, and it does not mean that this pathway cannot utilize any of the host's gene functions. For the sake of rigor, we have revised the title in the newly submitted manuscript to "Engineering the Paclitaxel Biosynthetic Pathway in *Physcomitrium patens* for De Novo and Stable Biosynthesis"

The most important evidence comes from two key experiments:

1. First, in the late-stage enzyme-omission experiment, the authors report that paclitaxel can still be detected when DBTNBT is omitted, and they interpret this result as evidence that P. patens may contain an endogenous enzyme with DBTNBT-like activity. This observation is highly significant because it directly supports the concern that the reported paclitaxel signal arises through fortuitous background metabolism in moss. If a terminal activity required for paclitaxel formation is supplied by an unidentified moss enzyme, then the pathway has not been reconstructed from the introduced gene set.

**Response:**

We are thankful for the reviewer's question. Here we provide a detailed explanation for the observation that paclitaxel can still be detected in the absence of DBTNBT: this phenomenon merely indicates the presence of a functional analog of DBTNBT in *P. patens*, and does not negate the function of DNTNBT itself. Four lines of evidence

support this:

(i) Overexpression of DBTNBT in *Taxus baccata* also markedly enhances the yield of paclitaxel (Perez-Matas, E. et al. *Plant Biotechnol. J.* 2023 Sep 29; 22(1):233-247);

(ii) Multiple independent studies have already demonstrated the essential role of DBTNBT in the biosynthesis of paclitaxel (Walker K et al., *Proc. Natl Acad. Sci*. 2002 June 27; 99:9166-9171; Zhang et al., *Mol. Plant*. 2023 Dec 4;16(12):1951-1961).

(iii) Exogenous supplementation of DBTNBT significantly increases the yield of paclitaxel (Figure R1);

(iv) Similarly, the production of paclitaxel was still detectable upon T3'NBT deletion (Figure R2) (Liang et al., Nat. Synth. 2025 April 30). By the same logic, the presence of an endogenous alternative enzyme should not be used to negate the function of T3'NBT.

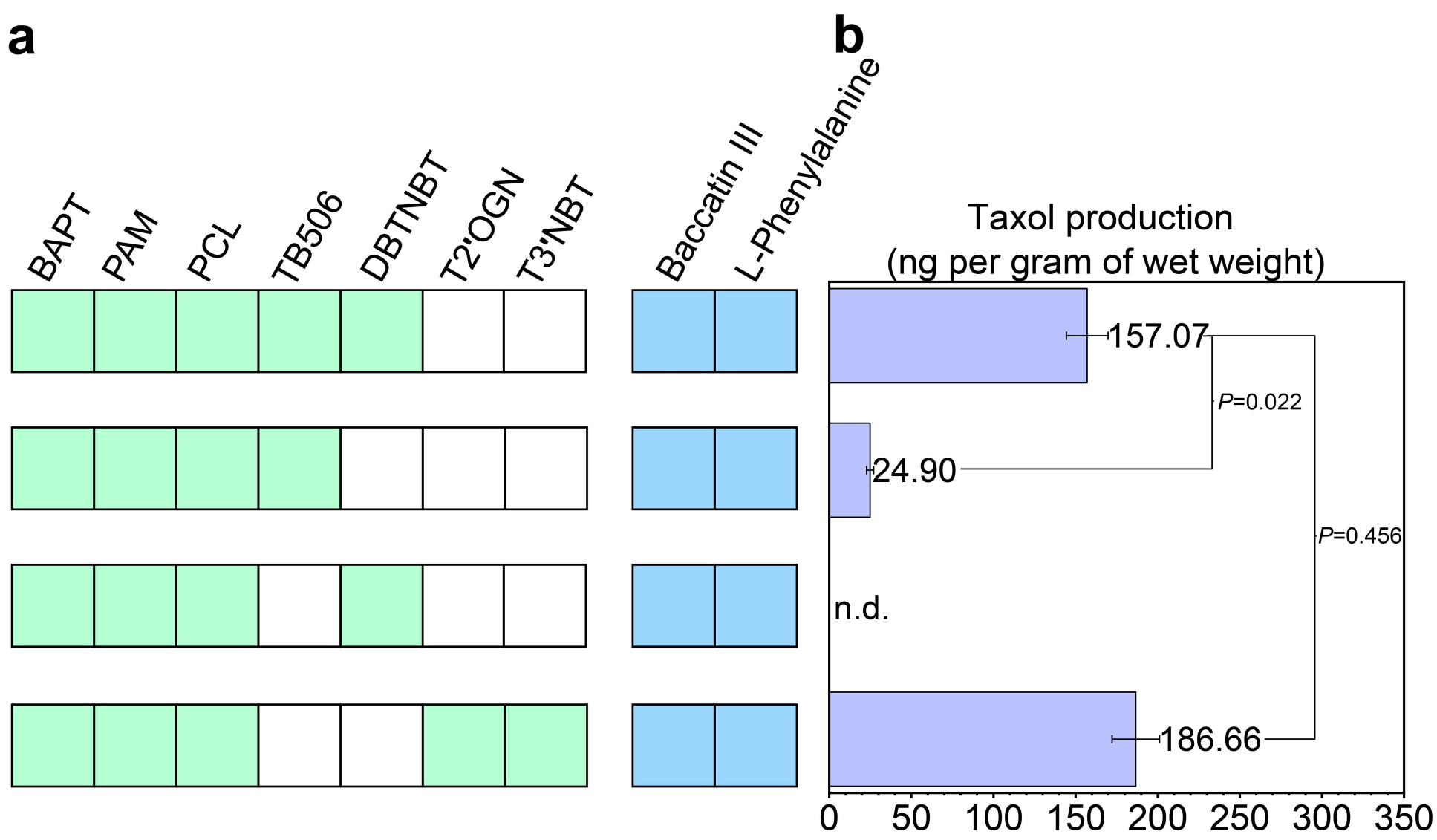


**Figure R1. Functional validation of TB506 and DBTNBT.**

(a) Schematic of enzyme combinations transiently expressed in *P. patens* protoplasts. Filled green squares denote overexpressed enzymes; filled blue squares indicate supplementation with the corresponding exogenous substrate (0.1 mM baccatin III and 1 mg/ml L-Phenylalanine). Blank squares represent the absence of the component.

(b) Quantification of paclitaxel produced by the transient expression of corresponding

enzyme combinations in (a). The one-way ANOVA p-values were used for significance difference analysis (*p*-values of 0.05 or less indicated statistical significance). Data are mean ± SD (n = 3). n.d., not detected.

REDACTED

Figure R2. T3'NBT catalyses the biosynthesis of Taxol from N-debenzoyl-taxol (3) (Liang et al., Nat. Synth. 2025 April 30).

2. Second, the same concern is reinforced by the experiment in which the authors report a marked increase in paclitaxel production after introducing FoTO1 and replacing the originally used terminal enzymes with T2′OGD and T3′NBT. These introduce key enzymes from more recent pathway models that were absent from the original reconstruction. The large effect of these additions strongly supports the point raised repeatedly in the previous rounds of review: the original gene set was incomplete, and the reported paclitaxel production depends on functions not included in the initial reconstruction.

**Response:**

We appreciate the reviewer for raising this point. Regarding this issue, our clarification is as follows:

The main conclusion of this study is that *P. patens* can be engineered into a plant

chassis for stable production of paclitaxel, and that production of paclitaxel using *P. patens* as a substrate can be achieved based on the initial reconstructed pathway and the optimized pathway.

We acknowledge that the original wording may have led the reviewer to mistakenly believe that this gene set includes all genes required for paclitaxel biosynthesis in *Taxus*. In fact, the biosynthetic pathway of paclitaxel is extremely complex, and multiple international teams are actively working to elucidate its mechanisms. More functional genes—including enzyme genes, auxiliary factors, regulatory factors, as well as the catalytic order and network architecture—will inevitably be identified in the future, all of which will further enhance the yield of paclitaxel.

Scientific rigor requires us to make it clear that this study does not claim to have used the "complete biosynthetic pathway of paclitaxel". Accordingly, we have revised the Title/Abstract/Introduction/Discussion sections to avoid any ambiguity. All revisions have been marked in red of the revised manuscript (lines 1-2, 8, 14-16, 81-86, 126-131, 137-139).

It is important to emphasize that the pathway we initially constructed was already sufficient to achieve the biosynthesis of paclitaxel base on *P. patens*, the addition of FoTO1 and the replacement of T2'OGD/T3'NBT with DBTNBT/TB506 led to a significant increase in paclitaxel yield. The results verify that the original constructed pathway and optimized pathway work well even without introducing the complete set of genes for compound paclitaxel biosynthesis.

3. These findings also reinforce another central concern raised, which has not been addressed in the revision: the pathway has not been validated step by step. The authors argue that endpoint detection of baccatin III or paclitaxel is sufficient to demonstrate pathway function. However, their own data show that this reasoning is flawed: paclitaxel is apparently detected even when an expected enzyme is omitted. Thus, endpoint detection cannot be used to infer that the introduced pathway functions as proposed. Each step must be validated with enzyme-addition and enzyme-omission controls, appropriate negative controls, and rigorous intermediate identification. Without this, the contribution of the introduced genes cannot be

distinguished from endogenous moss metabolism.

Regarding this issue, I would like to clarify the following:

First, it should be noted that the catalytic function of the genes used in this study has not been validated solely by us, but has received extensive support from published data by multiple research teams worldwide. Our core innovation lies in the integration of these paclitaxel biosynthesis-related genes, enabling the production of paclitaxel within chassis *P. patens*. From the perspective of synthetic biology, as long as the functional components are supported by existing literature, pathway integration does not require re-validation of each individual gene. This design strategy is widely accepted in the synthetic biology community.

Second, the main goal of this study was to obtain an engineered strain capable of stably producing paclitaxel, and to validate *P. patens* as an effective chassis for supporting paclitaxel biosynthesis. We aim to provide insights and technical references for future researchers to further optimize paclitaxel-engineered strains.

We fully understand the reviewer's concern regarding the rigor of experimental validation. However, in this study, the necessity of the gene set is supported by the literature. Moreover, the specific gene the reviewer have questioned has been sufficiently demonstrated at two levels: literature evidence and functional deletion experiments. Requiring step-by-step validation of all steps would entail a substantial investment of time, labor, and financial resources. Given that the current conclusions are already based on sufficient evidence and that re-validation would not materially affect the validity of the findings, we believe that conducting such comprehensive revalidation not be necessary.

4. This point is particularly important because the field of paclitaxel biosynthesis has advanced substantially beyond the pathway model on which this manuscript is based. The authors use the Zhang et al. pathway model. However, more recent pathway reconstructions, including those by McClune et al. and Liang et al., include several additional enzymatic and auxiliary functions that are absent from present manuscript. In addition to FoTO1 and T3NBT which were proven to be critical in the revised manuscript, omissions include the pathway-specific T9αH-750C rather than the

CYP725A22-1/CYP725A37 route used in the Zhang model; T7AT, which stabilizes the C7β-hydroxylated intermediate and facilitates productive 2α-benzoylation; TOT, which is required for oxetane formation; T1βH, which acts after oxetane formation; the deacetylases T9dA and T7dA; and T9ox, which completes the C9 oxidation state needed for baccatin III. Recent work also suggests that additional cryptic deacetylation chemistry, including C13α-O-deacetylation, may be part of the pathway logic (https://www.biorxiv.org/content/10.64898/2026.04.28.721278v1). Thus, the differences are not limited to one optional enzyme or one yield-enhancing factor; they affect the architecture of the pathway from the early oxidation step through baccatin III formation and the final conversion to paclitaxel.

**Response:**

We thank the reviewer for raising this point. Regarding the issue of the completeness of the gene set in this study, we would like to clarify as follows:

1. We acknowledge the reviewer's point "the field of paclitaxel biosynthesis has advanced substantially beyond the pathway model on which this manuscript is based". We have claimed this point in the revised manuscript (lines 81-86, 133-135, 374-376). However, multiple studies have demonstrated that the biosynthetic pathway of paclitaxel is a complex and dynamic network, rather than a single linear pathway (Liu et al., *Synth. Biol. J*. 2024, Apr 24, 5, 527-547; Shi et al., *Plant Commun*. 2025, Oct 13;6(10):101460; Yang et al., *Nat. Commun*. 2024, Mar 15;15(1):2339). The main conclusion of this study is that the gene set we used is capable of producing paclitaxel base on *P. patens*. This conclusion does not conflict with the reviewer's argument.

2. Regarding CYP725A22-1 raised by the reviewer, I have provided a detailed explanation in my response to question 7 and will not repeat it here. Similarly, the genes employed in this study include T1βOH, T9α oxidase and epoxidas, which are responsible for C1-hydroxylation, the C9-oxidation state, and oxetane ring formation, respectively. In contrast, T7AT, T9dA and T7dA are functional genes required for the intermediates in the reaction sequence of paclitaxel biosynthesis followed by McClune et al., which differs from the reaction sequence reported by Zhang et al. Therefore, they cannot be directly compared.

3. We recognize that the reviewer approaches the issue from the perspective of in-depth mechanistic elucidation of the paclitaxel biosynthetic pathway, pursuing

completeness and optimality. In contrast, our study is grounded in synthetic biology applications. Although the two perspectives differ, they are not contradictory.

Within the synthetic biology framework, multiple strategies exist for achieving the biosynthesis of a target product, for example:

(i) using a minimal gene set in a chassis cell;

(ii) employing artificially designed non-natural enzymes to construct a novel pathway;

or, (iii) as demonstrated in this study, constructing a stably heritable engineering strain for paclitaxel production based on the known gene set.

The core conclusion of our study is that we provide a feasible strategy for constructing a genetically stable paclitaxel-producing engineering strain using the known gene set. Although this pathway may not be the most complete or optimal, it offers a referenceable concept and methodology for subsequent researchers, thereby laying a foundation for the continuous improvement and optimization of paclitaxel-producing strains. It should be noted that numerous research teams worldwide have long been dedicated to elucidating the biosynthetic pathway of paclitaxel, with the ultimate goal of achieving efficient biosynthesis of paclitaxel—an undertaking of significant societal benefit. While the complete elucidation of the paclitaxel biosynthetic pathway remains a long and challenging journey, this should not halt the progress toward constructing paclitaxel-producing engineering strains. The value of our study lies precisely in taking this step forward, providing a viable starting point for the eventual large-scale production of paclitaxel.

5.FoTO1 is a clear example. The authors describe FoTO1 as non-essential because it is not a canonical catalytic enzyme. However, recent work shows that FoTO1 is not merely a minor yield-improvement factor as the authors claim. It changes the outcome of the first oxidation step, suppresses non-productive OCT/iso-OCT formation, and promotes formation of the productive taxadien-5α-ol-derived pathway (https://www.biorxiv.org/content/10.64898/2026.03.21.713420v1; https://www.biorxiv.org/content/10.64898/2026.03.30.715439v1). In the early-pathway data presented in this manuscript, taxadien-5α-ol appears to be only a small fraction of the oxygenated products, while OCT/iso-OCT dominate. Therefore, including FoTO1 is not optional. It is essential for proper pathway reconstruction.

**Response:**

We thank the reviewer for raising this point.

Regarding this point, I would like to clarify that we may be approaching this from different angles. We acknowledge that FoTO1 plays an important role in enhancing the yield of paclitaxel. However, even in the absence of FoTO1, the formation of taxadien-5α-ol can still occur in the presence of T5αOH alone, which subsequently leads to the generation of baccatin III and paclitaxel. Therefore, from our perspective, whether a gene is considered "essential" should be assessed based on the currently known repertoire of functional genes involved in paclitaxel biosynthesis. Specifically, we consider a gene essential only if its deletion completely abolishes the synthesis of paclitaxel and its function cannot be substituted by any other gene.

6. The same applies to oxetane formation. The authors dismiss the absence of TOT by referring to earlier pathway models in which baccatin III or related intermediates were reported without this enzyme. However, the formation of the oxetane-containing core is a defining step in paclitaxel biosynthesis. Multiple recent studies have identified specific oxetane-forming enzymes and have shown that this chemistry is not a trivial spontaneous process that can simply be assumed to occur in any host. If the authors propose that moss produces baccatin III and paclitaxel without a validated oxetanase, they must show the corresponding oxetane-containing intermediates and identify the activity responsible. Endpoint LC-MS detection of baccatin III or paclitaxel does not resolve this mechanistic gap.

**Response:**

We thank the reviewer for raising this point.

In our study, we employed epoxidase, which functions as epoxidation enzyme of C4-20, as demonstrated by Zhang et al. Moreover, we also validated the function of epoxidase in the first round of revision (Fig. R2). This function may require the support of endogenous enzymes from the plant chassis, and its specific mechanism awaits detailed elucidation in future studies.

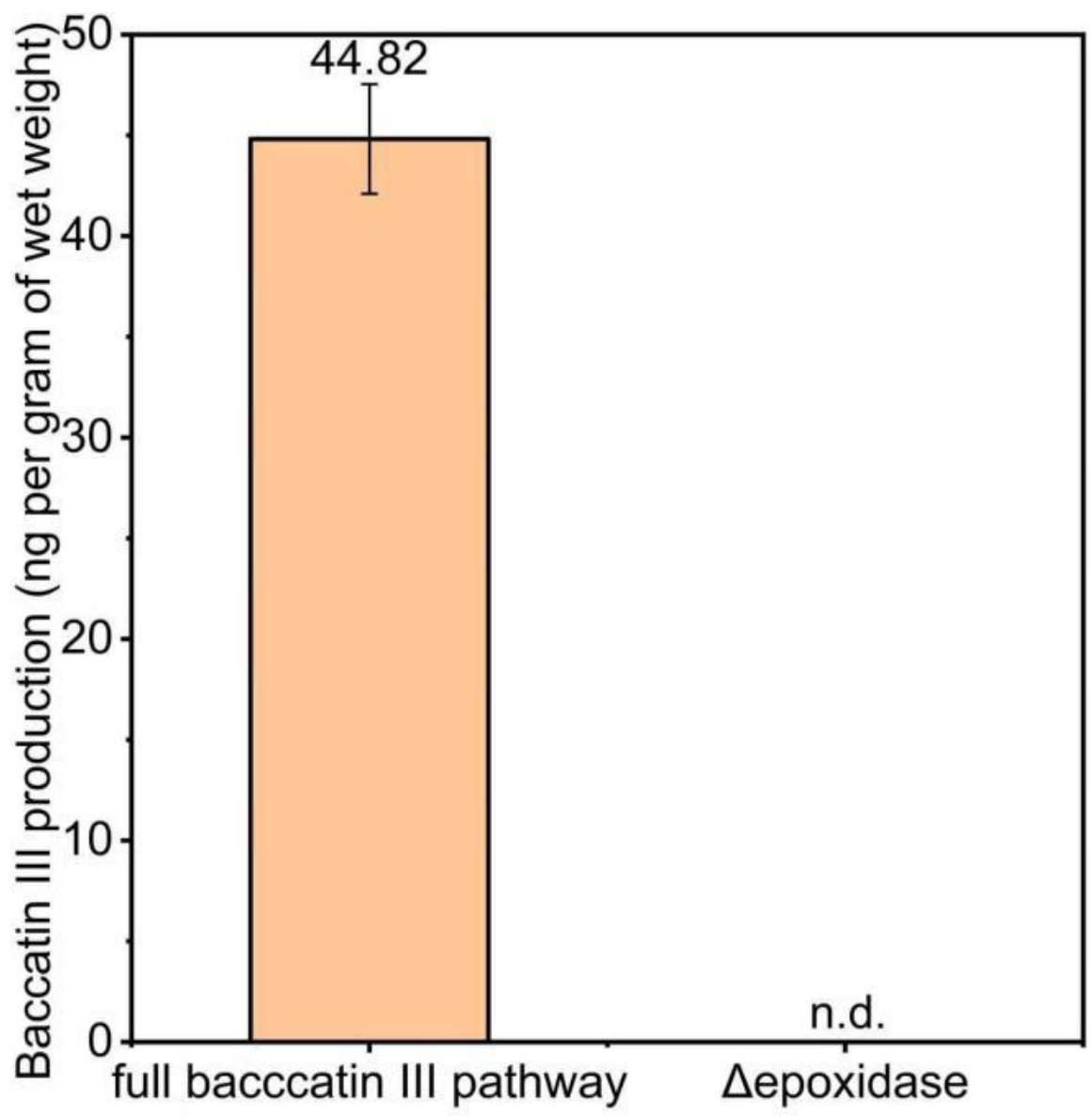


**Figure R2. Epoxidase is essential for baccatin III biosynthesis in the heterologous pathway.**

The "full baccatin III pathway" comprises genes encoding GGPPS-ΔCTP, TXS-ΔCTP, T5αOH, HMGR, CPR, epoxidase, TAT, T10βOH, T13αOH, T2αOH, T9αOH, T7βOH, T1βOH, TBT, and DBAT. The "Δepoxidase" pathway is identical but lacks the epoxidase gene. Data are presented as the mean ± SD of three biological replicates.

7. The authors' response also fails to resolve the additional mechanistic concerns about the utilized enzyme set around C13 acetylation. This is a critical flaw. The original point was not simply whether CYP725A22-1 and CYP725A37 are identical enzymes, or whether the Zhang et al. pathway model differs from the McClune et al. model. The concern is that the pathway proposed by the authors does not account for the substrate requirements and functional-group changes required to reach baccatin III and paclitaxel.

The authors respond that the 9α-hydroxylase used in their study is CYP725A22-1 from Zhang et al., and that this enzyme is distinct from the CYP725A37 discussed in other studies. I believe that the authors should confirm and compare the sequences of these enzymes more carefully. More importantly, they argue that Zhang et al. proposed a different reaction sequence and that their successful detection of baccatin III and paclitaxel demonstrates that this gene set is functional in moss. This argument is not sufficient. The authors still do not show that CYP725A22-1 acts on the actual intermediate generated in their moss system, nor do they identify the intermediates that would demonstrate this proposed reaction sequence.

This is important because the most recent pathway reconstructions raise a specific

mechanistic problem for the Zhang-type C9α-hydroxylase route. McClune et al. report that replacing T9αH-750C with T9αH-725A gives negligible baccatin III production, statistically indistinguishable from a ΔTDS negative control, and state that this is expected because T9αH-725A appears to require 13α-O-acetylation, which is absent from their baccatin III pathway and from Taxol. This directly relates to the concern raised in the review: if the authors use a C9α-hydroxylase route that requires a C13-acetylated substrate, then the pathway must also include the enzyme that installs the C13 acetyl group and the enzyme that later removes it to regenerate the C13 hydroxyl required for side-chain attachment. Neither step is accounted for in the manuscript.

**Response:**

We thank the reviewer for raising this point.

Response to the reviewer's concerns regarding CYP725A:

First, the catalytic function of CYP725A were well supported by published literatures (Jiang et al., Science. 2024 Feb 9;383(6683):622-629; Zhang et al., *Mol. Plant*. 2023 Dec 4;16(12):1951-1961). Multiple studies have demonstrated that members of the CYP725A family possess clear catalytic activity at position C9 in the biosynthesis of compound paclitaxel, indicating that CYP725A are not an uncharacterized or novel genes.

Second, the reviewer's comment that "replacing T9αH-750C with T9αH-725A gives negligible baccatin III production, statistically indistinguishable from a ΔTDS negative control," is not contradictory to our experimental system or conclusions, as the two situations are fundamentally different.

The replacement experiment mentioned by the reviewer (substituting T9αH-750C for T9αH-725A) represents a functional swap between distinct genes. The observed "negligible" result just indicates differences in substrate specificity or catalytic mechanism between T9αH-750C and T9αH-725A, and does not negate the function of T9αH-725A in its native or cognate pathway.

In contrast, we designed a transient co-expression assay based on the principle of a single variable. The complete group contained all genes required for producing Baccatin III (including T9αOH: CYP725A22-1), while the deletion group was identical except for the removal of T9αOH: CYP725A22-1. The results showed that

without T9αOH, Baccatin III was completely undetectable, whereas the complete group yielded significant amounts of Baccatin III (Figure R3). This demonstrates that CYP725A22-1 plays a decisive role in the "from-nothing-to-something" biosynthesis of Baccatin III, rather than serving a negligible auxiliary function. Therefore, the necessity of CYP725A22-1 in our study has been rigorously validated by a gene deletion assay, which addresses a different scientific question from the "negligible substitution result" cited by the reviewer and does not constitute a refutation of our conclusions.

Third, the conclusions from the study by McClune et al. cited by the reviewer contain four critical issues, and thus cannot be directly compared with the present work.

(i) Ambiguous gene identity: The gene CYP725A mentioned by McClune et al. was not specified with its exact variant. For the sake of rational discussion, we tentatively assume that all putative homologs of gene CYP725A were tested in their work. Even so, their findings do not contradict the results of our study.

(ii) Catalytic dependence on specific sequential assumptions: McClune et al. claimed that CYP750C directly catalyzes the conversion of 3O2A to 4O2A, whereas CYP725A only acts on 3O2A after its acetylation (Fig. S9). This statement relies on an unproven prerequisite: they presupposed the reaction sequence for hydroxylation at site C9, namely that hydroxylation at C10 and C13 and acetylation at C5 and C10 occur prior to the catalytic reaction mediated by T9aOH. To date, there is no scientific consensus on the chronological order of these hydroxylation and acetylation reactions (Liu et al., *Synth. Biol. J*. 2024, Apr 24, 5, 527-547). Different reaction sequences can lead to distinct substrate affinities and catalytic activities.

(iii) Additionally, the reviewer claims that C-13α acetylation is indispensable for the function of CYP725A, and thus denies the activity of CYP725A on the grounds that neither paclitaxel nor its precursor baccatin III contains a C-13α acetoxy group. As shown in Figures 4b and Fig. S9 in the work by McClune et al., CYP725A is capable of directly catalyzing the conversion of 3O2A to 4O2A, albeit with a relatively low yield.

(iv) There is also a contradiction here. According to the view of McClune et al. that

the function of CYP725A requires a C13-acetylated substrate, different have reported that paclitaxel (which lacks a C1 acetyl group) can be produced from CYP725A. This would imply that the plant chassis contains a C13-deacetylase. Consequently, in the plant chassis, when 3O2A, TAX19, and CYP725A are co-expressed, 4O2A should be detectable. That is, 3O2A would first be acetylated at C13 by TAX19 to generate the C13-acetylated intermediate, which would then be converted by CYP725A to taxusin, followed by deacetylation at C13 by the endogenous plant C13-deacetylase to yield 4O2A. However, the results shown in Figure 4b and Fig. S9 indicate that 4O2A was not detected. Therefore, it is likely that such a C13-deacetylase does not exist in the plant chassis. Even if such an endogenous enzyme does exist in plants, it does not affect the conclusion of this study: using the gene set described herein, paclitaxel can be biosynthesized in *P. patens* chassis cell.

Provided that McClune et al. did test all known homologs of CYP725A, combined with the proven catalytic activity of CYP725A reported in our study and other published literature, reasonable inference would be: (i) The catalytic activity of CYP725A is lower than that of CYP750C; (ii) The catalytic order of CYP725A is not the order presupposed by McClune et al., which is why no activity was detected. This just indicates that the function of CYP725A depends on a specific reaction context, rather than negating the necessity of CYP725A itself. Therefore, the results of McClune et al. do not conflict with those of the present study, and certainly cannot serve as a basis to negate the function of CYP725A22-1 in our work.

In summary, the results of McClune et al. are not inconsistent with our findings and cannot be used to refute the function of gene CYP725A22-1 identified in this study.

Recognition and revision regarding pathway complexity: We fully recognize that the biosynthetic pathway of paclitaxel is a complex and dynamic network, involving multiple isozymes, auxiliary factors, and regulatory factors. Therefore, although the initially constructed pathway contains the basic catalytic functions required, it cannot be claimed to encompass all elements of the complete biosynthetic pathway of paclitaxel. The reviewer’s concerns prompted us to re-examine the rigor of our wording.

In summary, the necessity of Gene CYP725A in this study has been rigorously validated, and the description of the synthetic pathway has been carefully revised for accuracy. These responses fully address the reviewer's concerns.

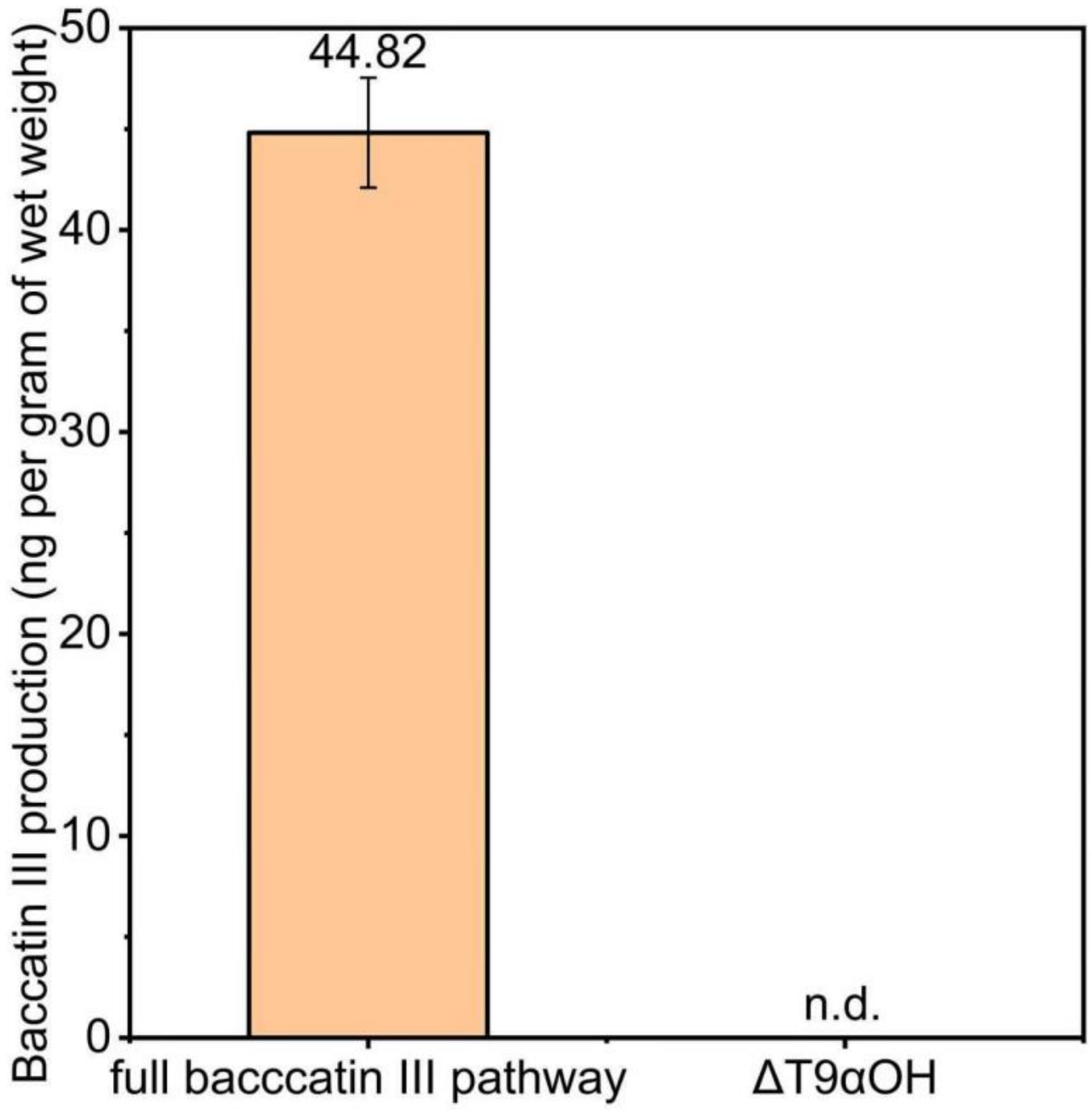


**Figure R3. T9αOH (CYP725A22-1) is essential for baccatin III biosynthesis in the heterologous pathway.**

The "full baccatin III pathway" comprises genes encoding GGPPS-ΔCTP, TXS-ΔCTP, T5αOH, HMGR, CPR, epoxidase, TAT, T10βOH, T13αOH, T2αOH, T9αOH, T7βOH, T1βOH, TBT, and DBAT. The "ΔT9αOH" pathway is identical but lacks the CYP725A22-1 gene. Data are presented as the mean ± SD of three biological replicates.

REDACTED

**Fig. S9. Replicate EIC for Figure 4b.** Extracted ion chromatogram (EIC) of indicated masses of extracts from three replicate *N. benthamiana* leaves expressing the indicated enzyme combinations. Blue shade highlights the biosynthetic pathway toward Taxol. **(McClune et al., Nature. 2025 Jul;643(8071):582-592)**

8. In addition to not clarifying the paclitaxel pathway, this distinction also affects the central biotechnology claim of this manuscript. If paclitaxel formation depends on unidentified moss activities, then the system cannot be rationally optimized, transferred, or engineered in the way implied by the manuscript. Without identifying the endogenous enzymes responsible for these missing steps, the authors cannot determine which reactions are limiting, which products are formed through the

intended route, or how the pathway should be improved. The reported fortuitous production therefore does not establish a validated and expandable platform for paclitaxel biosynthesis.

**Response:**

We thank the reviewer for raising this point.

First, we restate that the presence of an enzyme functionally similar to DBTNBT in *P. patens* does not negate the independent function of DBTNBT, as already elaborated in the first response.

Second, the use of plant chassis for the biosynthesis of natural products offers recognized advantages, including cellular compartmentalization, a diverse endogenous enzyme library, and abundant precursor supply. These advantages represent a rational starting point for plant chassis-based synthetic biology, rather than "confounding factors" to be avoided. We have claimed this point in the revised manuscript (lines 367-376). If the reviewer's requirement is to discard all advantages of the plant chassis and synthesize product paclitaxel solely via *in vitro* enzymatic reactions, that would essentially negate the research value of plant chassis itself.

Furthermore, by the same logic, studies elucidating the biosynthetic pathway of paclitaxel are also conducted using chassis cells. The use of any chassis inevitably introduces the possibility that endogenous enzymes from the chassis may influence the results. But if this were set as a standard, nearly all work utilizing plant chassis for natural product biosynthetic studies would be subject to the same doubt. This might not fully align with the common practice in the field. We therefore kindly ask that the sufficiency of our study be acknowledged based on the available evidence.

All enzyme components used in this study have been functionally validated in multiple published studies, with well-defined catalytic activity. Meanwhile, the initial reconstructed pathway and subsequent optimization strategies in this work are consistent with the biosynthetic rules of natural products and fully comply with the design principles and scientific logic for pathway reconstruction in synthetic biology.

Reviewer #4 (Remarks to the Author):

In my opinion, the manuscript reports an important and technically impressive advance. The authors have transferred a large paclitaxel biosynthetic gene set into *Physcomitrium patens* and demonstrate production of taxadiene-5α-ol, baccatin III, and paclitaxel through a modular engineering strategy. The establishment of a genetically stable moss line producing paclitaxel is a significant achievement and, in my view, is suitable for publication in Nature Communications, provided that the biochemical claims are phrased carefully.

We sincerely appreciate the reviewer's encouragement, affirmation, as well as the valuable comments and thoughtful guidance. The reviewer's suggestions have greatly benefited us and deepened our understanding of the importance of describing research findings in precise and scientifically accurate language.

First, DBTNBT has been functionally characterized in previous studies as a taxoid side-chain N-benzoyltransferase, including the original work by Walker, Long and Croteau and more recent work showing that overexpression of BAPT and DBTNBT can increase paclitaxel accumulation in Taxus baccata cultures. Therefore, the inclusion of DBTNBT in the pathway is biochemically justified. Nevertheless, because paclitaxel production is substantially reduced upon DBTNBT omission, the data still support a functional contribution of DBTNBT to the reconstructed pathway.

**Response:**

We sincerely thank the reviewer for the detailed explanations.

Second, the addition of FoTO1 and the replacement of TB506/DBTNBT with T2′OGD/T3′NBT should not be interpreted as disproving the original reconstruction. In metabolic engineering, it is common that an initial pathway is functional but later improved by newly identified enzymes or accessory factors. FoTO1 is now known to improve early taxane oxidation, and T2′OGD/T3′NBT provide an alternative and apparently more efficient terminal route. Their use strengthens the engineering result rather than negating the original pathway. However, the manuscript should clearly distinguish between the "initial reconstructed pathway" and the "optimized pathway." The strongest claim should be that moss can serve as a stable plant chassis for paclitaxel biosynthesis, not that the initially introduced gene set represents the uniquely complete or definitive Taxus pathway.

**Response:**

Many thanks to the reviewer for the thorough clarification. We have revised the manuscript to clearly distinguish between the "initial reconstituted pathway" and the "optimized pathway". This distinction indicates that *P. patens* can serve as a chassis for the stable biosynthesis of paclitaxel, and that the initially introduced gene set does not constitute the uniquely complete or definitive Taxus pathway. The revisions are marked in red in the revised manuscript (lines 81-86, 126-131, 133-135, 137-139 ).

Third, I partly agree that the pathway has not been validated step by step in the strict biochemical sense in this story while it has been validated by other research in Zhang et al 2023 Molecular plant. The authors validate major modules: taxadiene/taxadiene-5α-ol, baccatin III, and paclitaxel. This is acceptable for a synthetic biology reconstruction paper, particularly because many individual enzyme functions have already been reported in prior publications. However, the authors should avoid claiming full mechanistic resolution of every enzymatic step in moss unless all intermediates are directly detected.

**Response:**

Thank you very much for your valuable explanations and suggestion. We have revised the manuscript to avoid stating full mechanistic resolution of every enzymatic step in moss. The revisions are marked in red in the revised manuscript (lines 133-135, 375-377).

Fourth, the concern about unresolved C13-side-chain/late-stage biochemistry is fair. The current data do not fully establish the exact late-stage reaction sequence or all relevant intermediates in the moss chassis. In particular, if the pathway proceeds differently from the proposed linear sequence, or if moss endogenous enzymes participate, this should be acknowledged explicitly. However, this mechanistic uncertainty is not unusual in paclitaxel biosynthesis, which is increasingly understood as a branched and flexible biosynthetic network rather than a single invariant linear route. Therefore, I do not think this issue should preclude publication, but it should lead to more cautious wording.

**Response:**

We greatly appreciate the reviewer's comments. We have stated in the Discussion section that the DBTNBT/TB506 pathway different from the proposed linear sequence, and we also acknowledge the involvement of endogenous plant enzymes. The description has been corrected in the revised manuscript (lines 353-356, 358, 362-366).

Overall, I recommend publication after revision. The authors should be asked to temper the title/abstract/discussion claims so that the work is presented as a stable moss-based reconstruction and optimization of previous paclitaxel biosynthesis, rather than as definitive proof that every catalytic activity required for paclitaxel formation is supplied solely by the originally introduced gene set. They should also explicitly discuss the residual paclitaxel signal in the DBTNBT-omission experiment as evidence for possible endogenous moss acyltransferase activity or pathway flexibility. With these clarifications, the main conclusion—that P. patens can be engineered as a stable plant chassis for de novo paclitaxel production—is sufficiently supported.

**Response:**

We sincerely thank the reviewer for the valuable and constructive comments. We have revised all relevant statements throughout the manuscript (title, abstract, introduction, and discussion) to present this work as a stable plant - based reconstruction and optimization of previously established paclitaxel biosynthesis, not as definitive evidence that the originally introduced gene set alone provides every catalytic activity necessary for paclitaxel formation. Furthermore, we also discussed in detail the possible causes of the residual paclitaxel signal in the absence of DBTNBT, and elaborated on the flexibility of paclitaxel biosynthetic pathway.

We have revised the title to “Engineering the Paclitaxel Biosynthetic Pathway in *Physcomitrium patens* for De Novo and Stable Biosynthesis”. The revisions are marked in red in the revised manuscript (lines 1-2, 8, 14-16, 81-86, 126-131, 133-135, 137-139, 353-356, 358, 362-366, 374-376).

# Responses to the reviewers' comments (fourth round)

REVIEWERS' COMMENTS

Reviewer #4 (Remarks to the Author):

I think the authors have addressed all of my concerns. Given that the pathway reported by Zhang et al. in 2023 was first submitted to Nature, although it was not successful, I am pleased to see that our results can now be independently reproduced by another group. I believe this represents an important contribution to the field.

**Response:**

We sincerely thank the reviewer for the valuable time and effort, and we also express our genuine appreciation for the pioneering work of the reviewer's team in this field. These contributions have provided a solid theoretical foundation and important research insights for our study.
