# Supplementary material for: Engineering the paclitaxel biosynthetic pathway in Physcomitrium patens for de novo and stable biosynthesis
Source: Nat Commun. 2026 Oct 21;17:10648. doi: 10.1038/s41467-026-76625-x (PMC13687077; doi:10.1038/s41467-026-76625-x)
Supplement: Supplementary file 3 [file 41467_2026_76625_MOESM3_ESM.pdf]

Description of Additional Supplementary Files

Supplementary Data 1: List of genes used in this study.

Supplementary Data 2: List of primers used in this study.
