# Supplementary material for: Engineering the paclitaxel biosynthetic pathway in Physcomitrium patens for de novo and stable biosynthesis
Source: Nat Commun. 2026 Oct 21;17:10648. doi: 10.1038/s41467-026-76625-x (PMC13687077; doi:10.1038/s41467-026-76625-x)
Supplement: Supplementary file 4 [file 41467_2026_76625_MOESM6_ESM.pdf]

nature portfolio

Corresponding author(s): Zhangli Hu; Bin Jia

Last updated by author(s): 2026-07-07

Reporting Summary

Nature Portfolio wishes to improve the reproducibility of the work that we publish. This form provides structure for consistency and transparency in reporting. For further information on Nature Portfolio policies, see our Editorial Policies and the Editorial Policy Checklist.

Statistics

For all statistical analyses, confirm that the following items are present in the figure legend, table legend, main text, or Methods section.

n/a	Confirmed	
☐	☒	The exact sample size (n) for each experimental group/condition, given as a discrete number and unit of measurement
☐	☒	A statement on whether measurements were taken from distinct samples or whether the same sample was measured repeatedly
☐	☒	The statistical test(s) used AND whether they are one- or two-sided<br>*Only common tests should be described solely by name; describe more complex techniques in the Methods section.*
☒	☐	A description of all covariates tested
☐	☒	A description of any assumptions or corrections, such as tests of normality and adjustment for multiple comparisons
☐	☒	A full description of the statistical parameters including central tendency (e.g. means) or other basic estimates (e.g. regression coefficient) AND variation (e.g. standard deviation) or associated estimates of uncertainty (e.g. confidence intervals)
☐	☒	For null hypothesis testing, the test statistic (e.g. F, t, r) with confidence intervals, effect sizes, degrees of freedom and P value noted<br>*Give P values as exact values whenever suitable.*
☒	☐	For Bayesian analysis, information on the choice of priors and Markov chain Monte Carlo settings
☒	☐	For hierarchical and complex designs, identification of the appropriate level for tests and full reporting of outcomes
☒	☐	Estimates of effect sizes (e.g. Cohen's d, Pearson's r), indicating how they were calculated

Our web collection on statistics for biologists contains articles on many of the points above.

Software and code

Policy information about availability of computer code

Data collection	GC-MS data was collected with MSD ChemStation/MS Acquisition B.07.05.2479. LC-MS data was collected with MASSLynx V4.2 SCN997 and SCIEX Analyst software (V1.7) , respectively.
Data analysis	GC-MS data analysis was performed with MassHunter Qualitative Acquisition B.07.00. LC-MS data analysis was performed with MASSLynx V4.2 SCN997 and SCIEX MultiQuant(V3.0.3). Primers were designed using Primer 5 software. Fluorescence microscopy images were processed and analyzed using ZEN 3.1(blue edition) software.Statistical significance of LC-MS yields was assessed using SPSS (V27.0.1).

For manuscripts utilizing custom algorithms or software that are central to the research but not yet described in published literature, software must be made available to editors and reviewers. We strongly encourage code deposition in a community repository (e.g. GitHub). See the Nature Portfolio guidelines for submitting code & software for further information.

Data

Policy information about availability of data

All manuscripts must include a data availability statement. This statement should provide the following information, where applicable:

- Accession codes, unique identifiers, or web links for publicly available datasets
- A description of any restrictions on data availability
- For clinical datasets or third party data, please ensure that the statement adheres to our policy

All data essential for this study has been fully provided within the main text, supplementary materials, and source data files.

Research involving human participants, their data, or biological material

Policy information about studies with human participants or human data. See also policy information about sex, gender (identity/presentation), and sexual orientation and race, ethnicity and racism.

Reporting on sex and gender	N/A
Reporting on race, ethnicity, or other socially relevant groupings	N/A
Population characteristics	N/A
Recruitment	N/A
Ethics oversight	N/A

Note that full information on the approval of the study protocol must also be provided in the manuscript.

Field-specific reporting

Please select the one below that is the best fit for your research. If you are not sure, read the appropriate sections before making your selection.

☒ Life sciences ☐ Behavioural & social sciences ☐ Ecological, evolutionary & environmental sciences

For a reference copy of the document with all sections, see nature.com/documents/nr-reporting-summary-flat.pdf

Life sciences study design

All studies must disclose on these points even when the disclosure is negative.

Sample size	For both transient and stable transformation assays, three independent biological replicates were analyzed. For transient assays, each replicate consisted of one independent transformation batch. For stable transgenic lines, three independently generated lines were analyzed. For each line, tissues from multiple Petri dishes were pooled per assay to obtain sufficient biomass, and each pooled sample served as one biological replicate.
Data exclusions	No data exclusions.
Replication	We conducted three biological replicates for each individual experiment, and all of them were successful.
Randomization	The different experimental groups in this study correspond to distinct transgenic lines (i.e., different genotypes). Therefore, group allocation was based on the inherent biological characteristics of each line rather than on random assignment.
Blinding	Blinding was not applicable because all data acquisition and quantification were based on objective instrumental outputs with predefined parameters.

Reporting for specific materials, systems and methods

We require information from authors about some types of materials, experimental systems and methods used in many studies. Here, indicate whether each material, system or method listed is relevant to your study. If you are not sure if a list item applies to your research, read the appropriate section before selecting a response.

Materials & experimental systems

n/a	Involved in the study	
☒	☐	Antibodies
☒	☐	Eukaryotic cell lines
☒	☐	Palaeontology and archaeology
☒	☐	Animals and other organisms
☒	☐	Clinical data
☒	☐	Dual use research of concern
☐	☒	Plants

Methods

n/a	Involved in the study	
☒	☐	ChIP-seq
☒	☐	Flow cytometry
☒	☐	MRI-based neuroimaging

Plants

Seed stocks	In this study, the background of all strains was The Gransden 2004 wild-type of Physcomitrium patens subsp. Patens.
Novel plant genotypes	Part 1, Part 2, Part 3, Part 1/2, Part 1/2/3, Part 1/2/3-2, Part 1/2/3-3 stable transgenic lines were generated through homologous recombination.
Authentication	All stable transgenic lines can be distinguished through GC-MS or LC-MS.
